# Supplementary material for: A Qualitative evaluation in community settings in England exploring the experiences of coaches delivering the NHS Low Calorie Diet programme pilot to ethnically diverse participants
Source: BMJ Open. 2024 May 15;14(5):e085200. doi: 10.1136/bmjopen-2024-085200 (PMC11097852; doi:10.1136/bmjopen-2024-085200)
Supplement: Supplementary data [file bmjopen-2024-085200supp001.pdf]

Topic 1: Closed Background questions

What programme provider are they part of and what is your role with the provider ?

About their ethnicity?

Topic 2

1. Can you tell me how well you think the programme works for minoritised populations?
    i. Can you describe your role on the programme?
    ii. Can you give any examples of what you think works well?
    iii. Is there anything that doesn't work well?
    iv. What are your thoughts on the uptake and adherence of individuals from these groups on the programme?
    v. Do people from ethnic minority populations attend the sessions you have delivered?
    vi. If not what do they perceive are the barriers to being in the programme?
2. What training have you received to help you deliver to ethnic populations?
    i. How did you find the support that was provided?
    vii. Can you tell me about any training you have had to work with people from ethnic populations?
3. How is the programme tailored to support people from ethnic groups to access, engage and adhere?
    i. What impacts have you seen for individuals from the specific tailoring?
    ii. Does the overall programme work for ethnic populations why / why not?
4. Can you describe what have been your experiences of delivering the programme to individuals from ethnic backgrounds?
    i. Can you describe what has worked well?
    ii. Can you describe any challenges?
    iii. Can you describe what changes were made to support this population?
    iv. How has the programme or the delivery been tailored for ethnic populations?
    v. Were you able to resolve the barriers, and if so how?
    vi. How have the challenges you faced impacted your delivery of the programme?
5. How do you think the programme and delivery could be improved for ethnic populations?
    i. Can you describe your experiences of being a coach and delivering to ethnic populations - what works well / challenges / additional support needs?
6. What considerations, if any do you think have been made to support ethnic populations in completing the programme?

i. Can you describe what further considerations you think need to be made?
ii. Can you describe what you think of the considerations that have already been made?
iii. Can you tell me what improvements you think are needed to the programme to support those from ethnic populations?

Any questions?
