# Supplementary material for: A Qualitative evaluation in community settings in England exploring the experiences of coaches delivering the NHS Low Calorie Diet programme pilot to ethnically diverse participants
Source: BMJ Open. 2024 May 15;14(5):e085200. doi: 10.1136/bmjopen-2024-085200 (PMC11097852; doi:10.1136/bmjopen-2024-085200)
Supplement: Supplementary data [file bmjopen-2024-085200supp002.pdf]

Population living with type 2 diabetes.

In this section demographic information on the population living with diabetes is provided, following an estimated population that meet the eligibility criteria of the Low Calorie Diet programme. Figures in Table 1 are taken from the pilot site prospectuses compiled in mid-2019. Birmingham and Solihull, and North London Partners in Health and Care referenced the national Diabetes Audit (NDA) 17-18 as the source of their figures. Gloucestershire reference a Local Diabetes Audit 17-18, while Derbyshire referenced RAIDR 2019. The other six pilot sites do not provide a reference for where this information has been taken from.

Table 1. Population living with type 2 diabetes

Pilot Sites	People with Type 2 Diabetes	% Of Total Population
South Yorkshire and Bassetlaw	93,090	6.2%
Frimley Health and Care	29,950	3.7%
Greater Manchester HSCP	156,625	5.6%
Joined Up Care Derbyshire	50,273	5.0%
Birmingham and Solihull	74,250	5.7%
East London Health and Care Partnership	116,096	5.3%
Humber Coast and Vale	82,426	5.9%
Gloucestershire	27,309	4.2%
Bedfordshire, Luton and Milton Keynes	48,445	4.8%
North London Partners in Health and Care	69,530	4.3%
**Total:**	**747,994**	

Table 2: Overview of the LCD programme

Time	Three Phases of the Low Calorie Diet Programme
1-12 Weeks	**LCD:** with total diet replacement products (TDR) up to 900 calories per day. During this time patients will replace all normal meals with these products.
12-18 Weeks	**Food re-introduction:** during this time patients will be supported to re-introduce food, with a stepped phasing out of TDR products.
18-52 Weeks	**Maintenance**, during this time patients will be supported to maintain their initial weight loss.

During the TDR phase patients are expected to replace all normal meals with TDR products, which are provided by the provider for the duration of the service. Each service provider provides different TDR products such as soups, shakes, bars, and meal replacements such as porridge and daal, which all come in different flavours.

Ethnicity

The distribution of type 2 diabetes amongst different ethnic groups varies. The data in Table 3 are taken from the NDA, and so are based on the registers of patients collected and held at GP practices rather than directly from census data. Percentages may not total 100 because ethnicity has not been recorded in all cases.

Table 3: Ethnicity at pilot site level

Pilot sites	CCGs (2019/2020)	% with Type 2 diabetes by:	
		White	***Minority Ethnic***
South Yorkshire and Bassetlaw	NHS Barnsley	97.0	1.7
	NHS Bassetlaw	80.9	2.8
	NHS Doncaster	88.8	5.9
	NHS Rotherham	83.9	8.0
	NHS Sheffield	73.3	18.8
Frimley Health and Care	NHS East Berkshire	50.6	43.4
	NHS NE Hampshire and Farnham	67.6	17.6
	NHS Surrey Heath	67.5	16.2

Pilot sites	CCGs (2019/2020)	% with Type 2 diabetes by:	
		White	*Minority Ethnic*
Greater Manchester	NHS Bolton	62.9	29.4
	NHS Bury	56.5	12.7
	NHS Heywood, Middleton, Rochdale	66.3	30.6
	NHS Manchester	49.8	44.1
	NHS Oldham	60.7	35.1
	NHS Salford	75.4	10.0
	NHS Stockport	66.0	10.9
	NHS Tameside and Glossop	78.2	13.8
	NHS Trafford	64.8	20.7
	NHS Wigan Borough	86.2	4.1
Joined Up Care Derbyshire	NHS Derby and Derbyshire	79.5	9.9
Birmingham and Solihull	NHS Birmingham and Solihull	50.8	41.2
East London Health and Care Partnership	NHS Barking and Dagenham	41.8	54.9
	NHS City and Hackney	25.2	74.4
	NHS Havering	70.0	22.0
	NHS Newham	16.2	83.1
	NHS Redbridge	25.1	72.9
	NHS Tower Hamlets	20.1	79.0
	NHS Waltham Forest	31.3	63.5
Humber Coast and Vale	NHS East Riding of Yorkshire	74.7	3.9
	NHS Hull	83.5	5.5
	NHS North East Lincolnshire	84.6	11.1
	NHS North Lincolnshire	81.1	11.7
	NHS North Yorkshire	74.0	2.4
	NHS Vale of York	75.1	2.7
Gloucestershire	NHS Gloucestershire	75.5	5.6
Bedfordshire, Luton, Milton Keynes	NHS Bedfordshire	64.4	17.7
	NHS Luton	35.4	58.2
	NHS Milton Keynes	55.5	27.2
North London Partners in Health and Care	NHS North Central London	41.4	53.2

References

1. NHS Digital. (2020a) [MI] Ethnic Category Coverage. Available from <https://digital.nhs.uk/data-and-information/publications/statistical/mi-ethnic-category-coverage/current > [Accessed on 17th May 2021].
2. Office for National Statistics. (2011b). 2011 rural/urban classification. Available from <https://www.ons.gov.uk/peoplepopulationandcommunity/culturalidentity/ethnicity> [Accessed via SHAPE on 22nd June 2021].
