# Supplementary material for: A Qualitative evaluation in community settings in England exploring the experiences of coaches delivering the NHS Low Calorie Diet programme pilot to ethnically diverse participants
Source: BMJ Open. 2024 May 15;14(5):e085200. doi: 10.1136/bmjopen-2024-085200 (PMC11097852; doi:10.1136/bmjopen-2024-085200)

Theme: Experiences of cultural tailoring.

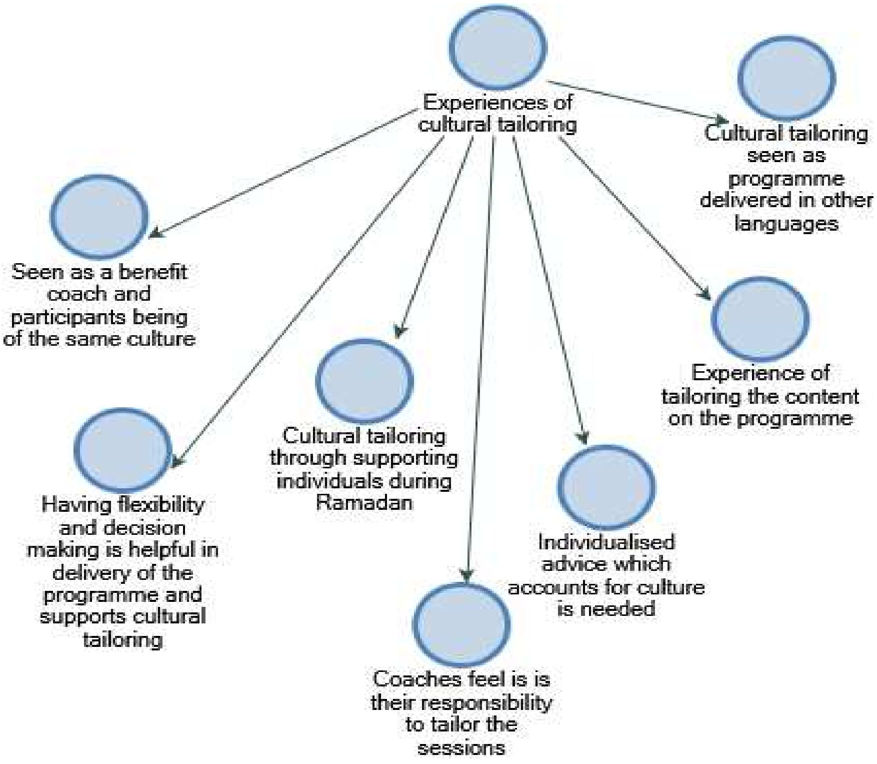

Theme: Needing to understand the impact of culture and ethnicity.

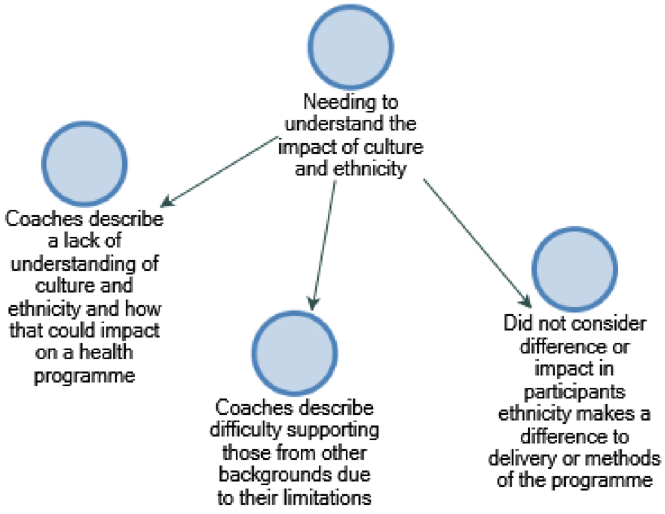

Theme: The impact of language.

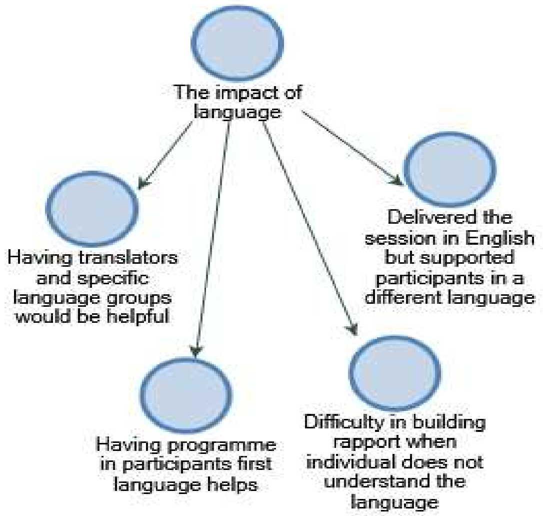

Theme: The use of resources in providing dietary advice.

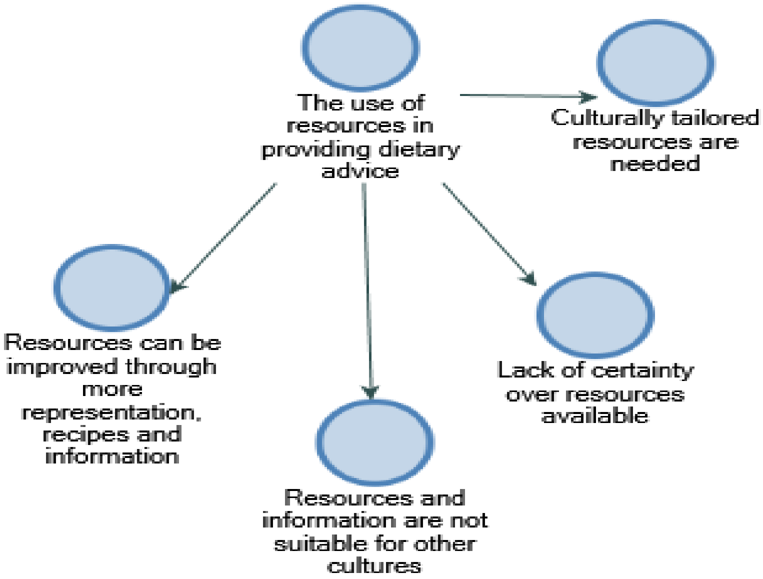

Theme: Training and support needs.

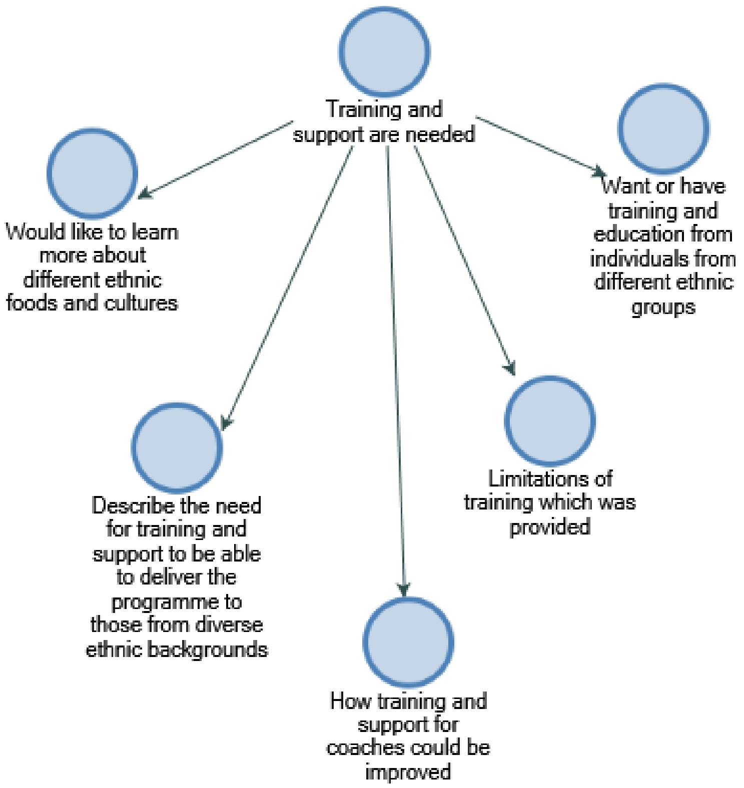

Supplement: Supplementary data [file bmjopen-2024-085200supp003.pdf]
