# Supplementary material for: A Qualitative evaluation in community settings in England exploring the experiences of coaches delivering the NHS Low Calorie Diet programme pilot to ethnically diverse participants
Source: BMJ Open. 2024 May 15;14(5):e085200. doi: 10.1136/bmjopen-2024-085200 (PMC11097852; doi:10.1136/bmjopen-2024-085200)

BMJ Open

BMJ Open is committed to open peer review. As part of this commitment we make the peer review history of every article we publish publicly available.

When an article is published we post the peer reviewers' comments and the authors' responses online. We also post the versions of the paper that were used during peer review. These are the versions that the peer review comments apply to.

The versions of the paper that follow are the versions that were submitted during the peer review process. They are not the versions of record or the final published versions. They should not be cited or distributed as the published version of this manuscript.

BMJ Open is an open access journal and the full, final, typeset and author-corrected version of record of the manuscript is available on our site with no access controls, subscription charges or pay-per-view fees (http://bmjopen.bmj.com).

If you have any questions on BMJ Open's open peer review process please email info.bmjopen@bmj.com

BMJ Open

Supporting ethnically diverse populations within a national diabetes intervention; a qualitative evaluation of the experiences of coaches delivering the NHS Low Calorie Diet Programme Pilot.

Journal:	*BMJ Open*
Manuscript ID	bmjopen-2024-085200
Article Type:	Original research
Date Submitted by the Author:	08-Feb-2024

Complete List of Authors:	Dhir, Pooja; Leeds Beckett University<br>Maynard, Maria; Leeds Beckett University, School of Health; Leeds Beckett University, Obesity Institute<br>Drew, Kevin J; Leeds Beckett University<br>Homer, Catherine; Sheffield Hallam University, Centre for HEalth and Social Care Research<br>Bakhai, Chirag; NHS Bedfordshire Luton and Milton Keynes STP<br>Ells, Louisa; Leeds Beckett University, Obesity Institute

Keywords:	Obesity, PUBLIC HEALTH, DIABETES & ENDOCRINOLOGY, NUTRITION & DIETETICS, General diabetes < DIABETES & ENDOCRINOLOGY, Health Education

SCHOLARONE™
Manuscripts

I, the Submitting Author has the right to grant and does grant on behalf of all authors of the Work (as defined in the below author licence), an exclusive licence and/or a non-exclusive licence for contributions from authors who are: i) UK Crown employees; ii) where BMJ has agreed a CC-BY licence shall apply, and/or iii) in accordance with the terms applicable for US Federal Government officers or employees acting as part of their official duties; on a worldwide, perpetual, irrevocable, royalty-free basis to BMJ Publishing Group Ltd ("BMJ") its licensees and where the relevant Journal is co-owned by BMJ to the co-owners of the Journal, to publish the Work in this journal and any other BMJ products and to exploit all rights, as set out in our licence.

The Submitting Author accepts and understands that any supply made under these terms is made by BMJ to the Submitting Author unless you are acting as an employee on behalf of your employer or a postgraduate student of an affiliated institution which is paying any applicable article publishing charge ("APC") for Open Access articles. Where the Submitting Author wishes to make the Work available on an Open Access basis (and intends to pay the relevant APC), the terms of reuse of such Open Access shall be governed by a Creative Commons licence – details of these licences and which Creative Commons licence will apply to this Work are set out in our licence referred to above.

Other than as permitted in any relevant BMJ Author's Self Archiving Policies, I confirm this Work has not been accepted for publication elsewhere, is not being considered for publication elsewhere and does not duplicate material already published. I confirm all authors consent to publication of this Work and authorise the granting of this licence.

For peer review only

Supporting ethnically diverse populations within a national diabetes intervention; a qualitative evaluation of the experiences of coaches delivering the NHS Low Calorie Diet Programme Pilot.

Authors: Pooja Dhir[1] (0000-0001-9225-0442), Maria Maynard[1] (0000-0002-0011-752X), Kevin J Drew[1] (0000-0003-0149-2521), Catherine Homer[2] (0000-0003-2571-6008), Chirag Bakhai[3] (0009-0008-4708-3305), Louisa J. Ells[1] (0000-0003-0559-4832)

Affiliations:

1 Obesity Institute, School of Health, Leeds Beckett University, Leeds, LS1 3HB, UK

2 Sport and Physical Activity Research Centre, Sheffield Hallam University, Olympic Legacy Park, 2 Old Hall Road, Sheffield S9 3TU, UK

3 NHS Bedfordshire, Luton and Milton Keynes Integrated Care Board, Arndale House, Luton, LU1 2LJ

Corresponding author: Pooja Dhir, Obesity Institute, School of Health, Leeds Beckett University CL10, Calverley Building, City Campus, Leeds, LS1 3HE.

p.dhir2675@student.leedsbeckett.ac.uk

Abstract 269 words. Word count: 4999

Conflicts of Interest: All authors confirm that they have no conflicts of interest to declare. LE has received funding from NIHR, MRC, Leeds Council and OHID/PHE in the last 3 years and has had an honorary contract with OHID.

CB is a primary care advisor to the national diabetes programme for NHS England.

Funding information: This work was supported by the National Institute for Health Research, Health Services and Delivery Research [NIHR 132075]. The NHS England Low Calorie Diet Pilot is funded by NHS England.

Contributor and guarantor information

"The corresponding author attests that all listed authors meet authorship criteria and that no others meeting the criteria have been omitted."

For peer review only - http://bmjopen.bmj.com/site/about/guidelines.xhtml

For peer review only

Transparency statement: The lead author affirms that this manuscript is an honest, accurate, and transparent account of the study being reported; that no important aspects of the study have been omitted; and that any discrepancies from the study as planned (and, if relevant, registered) have been explained.

Abstract

Background: The management of type 2 diabetes within diverse ethnic populations requires a culturally tailored approach. However, little is known about the experiences of coaches delivering interventions for type 2 diabetes (T2D), such as the NHS Low Calorie Diet (LCD) programme, to people from diverse ethnic backgrounds.

Objective: To explore the experiences of coaches delivering an NHS programme using total diet replacement approaches to individuals from diverse ethnic backgrounds, to inform the effective tailoring and equitable delivery of future interventions.

Design: Qualitative study.

Setting: Individuals delivering the NHS LCD programme.

Participants: One-to-one semi-structured interviews were conducted with seven health coaches delivering the NHS LCD programme. Inclusion criteria included participants delivering the NHS LCD programme being either from a minoritised ethnic background or delivering the programme to those from ethnic minority and White British backgrounds.

Main outcome measures: Qualitative semi-structured interviews conducted through different stages of the programme. Reflexive thematic analysis was used to analyse the transcripts.

Results: Key themes highlighted the following experiences of delivering the LCD programme: 1. Training and support needs; 2. Needing to understand culture and ethnicity; 3. The impact of language; 4. The use of resources in providing dietary advice; and 5. Experiences of cultural tailoring. The themes highlight the need to prioritise person-centered care, to integrate culturally tailored approaches and for provision of education and training to those delivering health programmes.

For peer review only - http://bmjopen.bmj.com/site/about/guidelines.xhtml

Conclusion: These findings describe the experiences of health coaches in tailoring delivery and emphasise the role of cultural competence in ensuring equitable and effective healthcare interventions for diverse populations. This learning can inform future programmes and policies aimed at promoting inclusive healthcare practices.

Strengths and limitations of the study

- This study provides insights into the challenges faced by health coaches, particularly concerning language barriers, resource utilisation, and cultural understanding when delivering the NHS Low Calorie Diet programme to ethnically diverse populations.
- Despite intending to do so, not all service providers of the NHS LCD are represented in the sample as there was a lack of uptake by service users and thus a lack of representation of service providers.
- A limitation of this study is the small sample size of participants, consisting of seven health coaches. While qualitative research often focuses on depth rather than breadth, a larger sample size could have enhanced the generalisability of the findings and provided a broader range of perspectives.
- A strength of this study is its focus on addressing an important gap in the literature regarding the delivery of healthcare interventions, specifically total diet replacement approaches, to individuals from diverse ethnic backgrounds.

What is already known about this subject

Prior research has highlighted the pressing need for culturally tailored interventions in managing type 2 diabetes within diverse ethnic populations. Studies have shown that type 2 diabetes disproportionately affects certain ethnic groups, including South Asian and Black ethnicities, emphasising the need for equitable healthcare strategies. While the importance of cultural competence in healthcare delivery has been recognised, there is limited knowledge about the specific experiences of health coaches delivering interventions like England's National Health Service (NHS) low calorie diet programme to ethnically diverse individuals.

For peer review only - http://bmjopen.bmj.com/site/about/guidelines.xhtml

Keywords: Type 2 diabetes, obesity, South Asian, qualitative, public health, thematic analysis, ethnicity, health care professionals, coaches

Abbreviations

T2D- Type 2 diabetes

UK- United Kingdom

HCPs- Health Care Professionals

US- United States

NHS- National Health Service

LCD- low calorie diet

NHSE- NHS England

Introduction

The prevalence of type 2 diabetes (T2D) remains a significant public health concern in many Western and industrialised societies[1,2], while its management requires a multifaceted lifestyle approach that encompasses dietary modifications.[1-3] However, food is culturally significant, especially among minority ethnic groups in the United Kingdom (UK), where it often serves as a cornerstone of traditions, identity, and social cohesion.[3] Thus, lifestyle change programmes are advised to consider the impact of culture and ethnicity, among other social factors when attempting to modify the dietary habits of individuals. With an increasingly diverse population in the UK, issues of culture and ethnicity are central to concerns over the equitable provision of healthcare services. [1, 4]

Health equity has been defined as the fair and just opportunity, irrespective of social position, to attain full health and well-being from social conditions that seek to promote and support good health.[4] The issue of equity is important because it is healthcare inequities that create, perpetuate, and exacerbate health inequalities.[5] Indeed, inequalities in health outcomes and access to healthcare are commonplace and addressing them is a public health priority.[5] For example, in response to the recognition that patients from different ethnic backgrounds may experience disparities in healthcare access and quality[6], the notion of

For peer review only - http://bmjopen.bmj.com/site/about/guidelines.xhtml

culturally competent, person-centredness and standardised practices in the delivery of services has been developed to try and meet the need for equitable delivery of healthcare services.[7,8,9]

Cultural competence in healthcare is described as the ability of healthcare systems to deliver interventions that encompass diversity in cultures, beliefs and behaviours while tailoring the service to individuals environmental, cultural and social needs.[10] Initially seen as a set of skills and knowledge that healthcare professionals (HCPs) possess to effectively engage with patients from diverse backgrounds[11], cultural competence has evolved to encompass HCPs' ability to deliver interventions. This delivery should not only accommodate diversity in beliefs and behaviours, but also the ability to tailor services to individuals' environmental, cultural, and social needs, including different food preferences.[11] Healthcare professionals need to possess cultural competences in order to address the needs of the diverse patient populations they deliver to, and thus, deliver healthcare services in a more equitable way.

Existing research from the perspectives of patients often underscores the need for culturally relevant support, such as through cultural competency of HCPs and culturally tailored resources such as dietary resources.[12] For example, In the United States (US), health coaches have been reported to value shared characteristics with their clients which has helped to build a trusting relationship and support with decision making.[13] More generally, previous research has explored the experiences of HCP-participant interactions and highlighted the significance of coach-participant relationships in enhancing health behaviours and outcomes.[14,15] Similarities between coaches and patients, coupled with the time spent together, facilitated the establishment of a strong coach-patient relationship.[14] Within this relationship, four key coaching activities emerged: education, personal support, practical assistance, and serving as a liaison between patients and clinicians.[14] However, limited research is available on the experiences of the healthcare providers in the context of supporting minoritised ethnic participants within T2D management, or within low calorie diet, total diet replacement interventions in the United Kingdom (UK).[9,14,15] Furthermore, there is a dearth of evidence relating to the practical challenges, insights, and experiences of HCPs or coaches who are working to an agenda that advocates for culturally relevant support.[16] In this

For peer review only - http://bmjopen.bmj.com/site/about/guidelines.xhtml

context, in England, the National Health Service (NHS) recently piloted a low calorie diet programme to promote remission of T2D.

The NHS Low Calorie Diet Programme

The NHS Low Calorie Diet (LCD) programme pilot was launched in 2020 (renamed the Type 2 Diabetes Path to Remission programme when rolled out nationally in June 2023). Available to adults (18-65 years) living with a BMI ≥27kg/m² (adjusted to ≥25kg/m² for Black, Asian and other ethnic groups) and T2D diagnosis within the last 6 years, the NHS LCD programme pilot was initially mobilised in 10 integrated care systems[1] (referred to hereon in as localities)[17,18] and expanded to a further 11 localities in 2022. The 52-week programme

Time	Three Phases of the Low Calorie Diet Programme
1-12 Weeks	**LCD:** with total diet replacement products (TDR)c up to 900 calories per day. During this time patients will replace all normal meals with these products.
12-18 Weeks	**Food re-introduction:** during this time patients will be supported to re-introduce food, with a stepped phasing out of TDR products.
18-52 Weeks	**Maintenance**, during this time patients will be supported to maintain their initial weight loss.

piloted across a total of 21 socio-demographically diverse localities in England, included a 12-week TDR phase which consisted of micronutrient-complete foods such as bars, shakes and soups of various flavours (estimated 800- 900kcals/day) for the first 12 weeks, followed by a 6-week stepped food reintroduction phase and weight maintenance support until programme end[19,20] with behaviour support provided through one of the three models: 1:1, group or digital. Commissioned by NHS England (NHSE), the programme was delivered by six commercial providers, utilising a predominantly non-medical workforce such as health coaches to deliver the sessions to service users.

Table 1: Overview of the LCD programme

[1] Integrated care systems are partnerships between NHS bodies, local authorities, and local organisations which work together on health and care services to improve the lives of people locally.

For peer review only - http://bmjopen.bmj.com/site/about/guidelines.xhtml

The service specification mandates the promotion of equal access and the tailoring of support through the application of equity based policy (further description of the programme has been provided elsewhere).[21,22] Preliminary (unpublished) data from the NHS LCD Programme highlights that 18% out of estimated 21% eligible people with Asian ethnicity, 8% out of 8% of those with Black ethnicity, vs 66% of those with White ethnicity were referred to the programme.[23] The preliminary data shows that individuals from a South Asian and Black ethnicity have lower programme uptake than those of White ethnicity (61% and 67% vs 72%) and lower 12-month weight loss (6.8% vs 10.3%).[23] The overall dropout rate at 12 months was 55% for those from Asian ethnicity, there was no significant differences by ethnicity in the univariate analyses and on the multivariable logistic regression.[24]

This paper, therefore, aims to explore the experiences of health coaches who deliver the NHS LCD programme to ethnically diverse populations, while identifying the presence or not, of equitable and culturally tailored care. Given the disproportionate impact of T2D on certain ethnic groups, the study bridges the significant knowledge gap regarding the challenges, successes, and nuances faced by coaches in culturally tailored diabetes management interventions, in order to inform future service development.

Methods

This study formed part of the large Re:Mission study evaluation of the NHS LCD programme[25]. The study received ethical approval from Leeds Beckett University (LBU 106559) and is reported using COREQ guidelines (see supplementary file 1). Employing a qualitative research design, this study centres on the subjective views of the coaches whilst recognising that these experiences will be shaped by underlying structural, cultural, and contextual factors.[26] The full research process was conducted over a period of 12 months which included recruitment, interviews, analysis and write up, with interviews being conducted in March 2023, in order to allow engagement with participants across the three stages of the programme. Data collection and data analysis were carried out by PD who identifies as a female of Indian ethnicity (details on co-authors are reported in supplementary file 1).

Materials and procedures

For peer review only - http://bmjopen.bmj.com/site/about/guidelines.xhtml

The interview questions broadly covered participants' experience of the programme, such as their training, challenges faced, successful strategies employed, and suggestions for programme improvement and cultural tailoring. (See Supplementary file 2 for the interview guide). Written informed consent through a completed consent form and socio-demographic information were obtained prior to the interviews, consent was re confirmed verbally at the beginning of the interview. All of the semi-structured interviews were conducted by PD via Microsoft Teams which lasted 30-60 minutes. All the interviews were audio-recorded (with permission); the interviews were transcribed verbatim by an external transcriber. There was no Patient and Public Involvement in this study.

Participants

The recruitment process involved engaging four service providers authorised by the National Health Service England (NHSE) to deliver the programme. Subsequently, service providers disseminated an email containing the participation information sheet and details about the research to health coaches. These coaches then extended invitations to potential participants, resulting in the purposive recruitment of seven health coaches through two NHSE-authorised service providers. No financial incentive was provided. Ten coaches consented to be interviewed however three had to withdraw due to time constraints, the remaining were all interviewed.

The coaches worked in diverse geographical areas including Frimley, Greater Manchester, Derbyshire, Birmingham and East and North London and delivered the programme to those from ethnic minority backgrounds.[27] One of these recruited participants conducted the programme in a language spoken by South Asian minority groups. Supplementary file 3 provides background information on the programme and demographic data.

Inclusion criteria included participants delivering the NHS LCD programme being either from a minoritised ethnic background or delivering the programme to those from ethnic minority and White British backgrounds. The study proceeded with interviewing all remaining coaches who contacted us and were available to partake in the interview process. Among those recruited, self-reported ethnicity included Pakistani (n=2), Indian (n=1) and White British (n=4). Supplementary file 3 provides data on how many individuals with T2D were eligible for

For peer review only - http://bmjopen.bmj.com/site/about/guidelines.xhtml

the programme from different demographic areas in which the pilot programme was based and also the percentage of individuals with T2D from White and minority ethnic backgrounds.

Table 2: Participant Characteristics

Coach number	Programme delivery	Programme provider	Self-reported ethnicity	Gender
1	Online group	1	Asian Pakistani	Female
4	Online group	2	Pakistani	Female
6	Online 1:1	2	British Indian	Male
2	Online 1:1	2	White British	Female
3	Online group	2	White British	Female
5	Online 1:1	2	White British	Male
7	Online group	2	White British	Male

For peer review only

Data analysis

Transcripts were transcribed by an external transcriber and checked for accuracy by one researcher (PD). NVivo (version 12) software was used to facilitate data management.

A reflexive thematic analysis, as proposed by Braun and Clarke[28], was conducted on anonymised interview data used to explore the challenges, successes and personal reflections of coaches in delivering the programme. PD re-read interview transcripts multiple times to gain familiarity with the data, whilst annotating relevant extracts and noting broader ideas that could aid the coding in subsequent stages. Themes were then generated and revised iteratively and discussed with MM who as suggested by Sparkes and Smith acted as a critical friend after each of the stages of analysis to enhance the reflexive self- awareness of interpretations. Field notes were used to enhance this reflective process, at each of the six stages of analysis. Supplementary file 4 outlines the thematic map.

For peer review only - http://bmjopen.bmj.com/site/about/guidelines.xhtml

This methodology enables a flexible process to look at contextually embedded phenomena, such as cultural competence, communication barriers and the impact of coaches' backgrounds on interactions. Further detail on the reflexive process is highlighted in the COREQ checklist (see supplementary file 1). Malterud, Siersma, and Guassora's (2016) concept of information power can be used within reflexive thematic analysis as an alternative to data saturation as described by Braun and Clarke (2021).[29,30] This approach allows an interpretive judgement regarding study size related to the purpose and goals of the analysis.

Results

Five themes were identified, which had a focus on the effectiveness and inclusivity of the programme, these are: training and support needs, the impact of language, the use of resources in providing dietary advice, needing to understand the impact of culture and ethnicity and experiences of cultural tailoring.

1. Training and support needs.

Coaches highlighted the challenges experienced when supporting participants with different ethnicities to their own, in relation to cultural food preferences and considerations associated with their ethnic backgrounds. They discussed limitations in the training they received, including a lack of specific training for working with service users from different ethnic backgrounds and cultures to theirs, and a lack of education about different cultures, multi ethnic foods and their impacts on health:

'So I wouldn't say that I had training for ethnic populations.' (Asian-Pakistani, female, C1)

'*there's not, there's not been much training on the diverse cultures.*' (White- British, female, C2)

Coaches reported several areas where training and support could be improved, which included a deeper understanding of culturally specific foods, understanding the cultural influences that affect dietary choices, and any additional considerations linked to ethnicity such as festivities and social support. Practical suggestions for training included learning

For peer review only - http://bmjopen.bmj.com/site/about/guidelines.xhtml

about food groups and common foods within cultures, training days for cultural competencies, education on barriers and influences within specific cultures, and guidance on adapting the programme content to suit participants' cultures.

'*the challenges will still be there unless I get some training on foods or how cultures might be affected by the programme.'* (White-British, female, C2)

They also wanted education and external training from dietitians and health professionals from diverse ethnic backgrounds with an understanding and experience of relevant cultures and lifestyles, to support appreciation and understanding of the nuances within ethnicities. Furthermore, coaches highlighted wanting support from a workforce that was also ethnically diverse, which would enable peer support between colleagues from different backgrounds to respond to the needs of participants.

'*I think yeah just having good training is a good thing. I would say training from people of that background. So maybe dieticians who work, you know, are from that ethnicity or that background I think is better…*' (Asian-Pakistani, female, C1)

2. Needing to understand the impact of culture and ethnicity.

Coaches described a limited understanding of culture and ethnicity and how these factors could influence the effectiveness of health programmes. They described their knowledge gaps, with one participant reporting uncertainty about what is meant by ethnicity. Furthermore, there was discussion on a lack of awareness of different religions and ethnicities with one participant reporting confusion on what was meant by 'South Asian ethnicity'. Some coaches from a White British background reported difficulty in supporting participants from backgrounds different from their own, due to their limited knowledge of dietary practices and cultural nuances.

'*…it is affecting my participants if I'm not fully understanding food, culture, or anything about their background really.*' (White- British, Female, C2)

'*African backgrounds because they tend to have a lot of flour-based foods in their diet and the advice is not really sort of geared to give them practical examples of how they could adjust their diet to make that work. And I, you know I would, I would have to look that up myself, you know, if I was asked that.*' (White -British, Female, C3)

For peer review only - http://bmjopen.bmj.com/site/about/guidelines.xhtml

The gap in their understanding led to some coaches resorting to using a generic approach, with one coach believing that treating every participant the same was better and more ethical, rather than providing tailored advice regarding culture and ethnicity. However, this was regarded at times as being due to the lack of understanding on how to tailor advice and a lack of understanding of the importance of tailoring. Furthermore, some coaches highlighted that before being asked about ethnicity as part of the current study, they had not considered how participants' ethnicity could impact the delivery and methods of the programme.

'…you know I'm still ignorant to a good portion of cultures from around the world.' (White- British, male, C7)

'*I've never until I was invited for this interview thought about making a distinction between people's backgrounds and their results. So, I'll find that really difficult to answer because I never sort of looked at it in that way…*' (White- British, Female, C3)

Other coaches acknowledged that a deeper understanding of how food and culture intersect could enhance the quality of the service they provide, with some coaches reporting they consider the difference in cultures when delivering the programme and adapt the delivery according to this.

'*it is affecting my participants if I'm not fully understanding food, culture, or anything about their background really*.' (White British, female, C2)

3. The impact of language.

Coaches from a White British ethnicity described difficulty in communicating with participants due to language differences, creating a challenge in building rapport. It was mentioned that, at times, family members were involved as translators to overcome language barriers, and without this the participants would have faced challenges in understanding the programme's content. In general, coaches' views were that incorporating interpreters and specific language groups within the programme could increase inclusivity and improve adherence. In turn this would support an environment where individuals with a different preferred language could fully engage and benefit from the programme.

'We need to consider other language groups so that we make it inclusive for everybody.' (White British, female, C2)

For peer review only - http://bmjopen.bmj.com/site/about/guidelines.xhtml

'…an Urdu specific group to help tailor, to help sort of support those, so I feel like that is something that that, that's good, that helps and can help sort of like reach those that might not be able to join the programme, if because they won't, might not be able to follow it or understand what's going on.' (White-British, male, C5)

4. The use of resources in providing dietary advice.

Experiences of inadequate resources and uncertainty of resources available such as culturally tailored resources were described, particularly from coaches of White British ethnicity. This was described as leading to difficulty in providing culturally tailored guidance. Coaches of both White British and South Asian ethnicity described the resources and information as being centred around a Western diet, which created a barrier when supporting those from other cultural backgrounds.

'a lot of the information is based on a typical Western diet.' (White British, female, C3)

'having like maybe different resources so you could go into a conversation with a specific resource open to know what to be talking about in certain areas might be useful.' (Pakistani, female, C4)

Coaches highlighted that for resources to be effective, they needed to reflect the cultural background of the programme's participants. They suggested resources can be improved through incorporating culture-specific recipes and further representation in the information and guidance.

'The programme could benefit from a lot more diverse and wide range of recipes' (White-British, female, C2)

'Maybe making some recipes that would be just tailored towards them. That could be something that we could look at, you know, do. Maybe even and digging into how we can actually break that barrier of you know getting these service users onto the programme without having them to think, oh, these are not the foods that we regularly have so how am I going to have that while I'm sitting with my family?' (Pakistani, female, C4)

5. Experiences of cultural tailoring.

Coaches described a sense of responsibility for adapting programme sessions to align with the cultural backgrounds of participants. Commonly, cultural tailoring was perceived as

For peer review only - http://bmjopen.bmj.com/site/about/guidelines.xhtml

For peer review only

delivering the content in other languages and providing support and specific resources during Ramadan. Therefore, there was a limited view of cultural tailoring. Cultural tailoring was at times perceived as providing advice to reduce the quantities of cultural foods such as chapatis.

'…it's cutting down on rice and cutting down on chapattis and things.' (White-British, male, C7)

Coaches of South Asian ethnicity reported further cultural tailoring through individualised advice and customising the content of the session, and also reported the positive impact of sharing the same ethnicity as the participants. These coaches highlighted the benefit of speaking the same language as participants and that their personal experiences and cultural understanding enabled them to relate to participants and build rapport. Furthermore, coaches discussed the importance of flexibility around decision-making as it allowed them to effectively tailor the programme content to the cultural preferences and needs of their participants. An example of this is a coach who reported removing a session about alcohol because it was not relevant to her group of Muslim women.

'…explaining things in a language that other people understood potentially it was quite helpful…' (British-Indian, female, C6)

Table 2: Participant quotes and themes

Quote	Theme	Participant details
So maybe just having another coach who you can kind of talk to every month whenever you have a session to discuss your group so that they kind of have an insight as well, and someone just to come and watch your group as well.	Training and support needs	C1 (Asian Pakistani, Female)
I think even just maybe having some more exposure on the food groups, I think that would be something that we could look into, you know which would help the service users.	Training and support needs	C4 (Pakistani, Female)

For peer review only - http://bmjopen.bmj.com/site/about/guidelines.xhtml

We took, we've got topics on Ramadan that so we send out like leaflets and information on that, but it's not a topic that I would say I'm fully competent in when you ask me the questions on that. So I don't think we've had enough training, to be honest.	Needing to understand the impact of culture and ethnicity.	C2 (White British, Female)
I'm very experienced in giving people suggestions, meal suggestions that could be, you know, better choices for diabetes management than the diet that they were having, and not so familiar with, you know, looking for alternatives in, in Asian food.	Needing to understand the impact of culture and ethnicity.	C3 (White British, Female)
What would be better is having, if people are not fluent in English, having more language, language options on the app or the recipes or the, the use of certain tools in the app.	The impact of language.	C2 (White British, Female)
providing them with that language, you know, language content, you know, a coach that could speak that language or, you know, somebody that could check in with them even if the coach, if they don't have a coach that's available for their language, just to see how things are. If there is anyone in the office that could, you know speak Urdu, Hindi, Punjabi, what other ethnic, you know Asian ethnic languages are, just to you know see the service user is in comfort when they are joining the session, they don't have any questions, they're not lost.	The impact of language.	C4 (Pakistani, Female)
Because then they might, they will feel rather than it just all being around maybe focused around sort of like a Western diet, they can then feel like it's more directed for them and they're sort of getting that knowledge that it's going to be more beneficial to them rather than, rather than something that they're not gonna really have much interest in and there only being like, slight, slight add-ons about their own diet.	The use of resources in providing dietary advice.	C5 (White British, Male)

For peer review only - http://bmjopen.bmj.com/site/about/guidelines.xhtml

So we have specific ideas for different cuisines and it wouldn't just be for the South Asian, but it would also have to be for African, Afro Caribbean and those are sort of the main groups that we, yeah that we are aware of.	The use of resources in providing dietary advice.	C3 (White British, Female)
So you have to convey all of the concepts and ideas and kind of all of that, of course, but it's just how you do it. So, but even things like you know, recipe ideas, we had a lot that were relevant to my group. And also kind of Indian populations as well.	Experiences of cultural tailoring	C1 (Asian Pakistani, Female)
I think people do appreciate it when you just put the time in to sort of take on what they're saying and take on, like especially cultural things because people are quite proud of, of their culture a lot of the times. So just, you know, knowing that it's being appreciated, it's being considered, you know, it's not just being brushed off and going OK, well we have this way of doing things so you're gonna abandon your culture basically to eat how we wanna tell you to eat, and that's just not gonna work for anyone.	Experiences of cultural tailoring	C7 (White British, Male)

For peer review only

Discussion

The themes highlight the varying cultural competency of coaches in their understanding of cultures and ethnicities and the potential impact of knowledge gaps such as tailoring on programme delivery. The study provides insight into delivering the LCD programme, emphasising the need for comprehensive training, addressing language barriers, utilising culturally tailored resources, understanding diverse cultures, and implementing effective cultural tailoring strategies.

Culturally relevant support

The multifaceted concept of cultural tailoring encompasses linguistic adaptations, cultural understanding, and the development of tailored resources, through the use of guiding

principles for cultural tailoring and cultural competency to support this.[31] Whilst this research reveals the presence of important 'surface-level' adaptations within the NHS LCD programme such as the Urdu language group and some ethnic-specific resources such as for Ramadan, it also underscores the need for 'deeper' level adaptation, to cultivate a thorough comprehension of the cultural contexts, beliefs, and values that influence individuals' health behaviours.[32(p2-3)] 'Deep structure' adaptations are described as those which go beyond superficial adjustments and incorporate understandings of cultural underpinnings, and are more likely to result in behaviour change through aligning with frameworks for cultural tailoring such as through creating culturally relevant educational materials and incorporating traditional dietary practices which incorporate understanding of core cultural values and how a range of social, ethnic, environmental and historical beliefs can shape health beliefs and behaviours.[31-33]

The coaches' uncertainty about available resources and the cultural misalignment of existing materials highlights areas where improvements are required. Through enhancing representation, diversifying recipes, and providing culturally tailored information and resources, programmes can better meet the needs of ethnically diverse populations. The coaches described the potential benefits of incorporating professional interpreters and establishing specific language-based support groups, ultimately working toward a more inclusive and effective programme for participants from diverse backgrounds. Similarly previous research has highlighted challenges of cultural competency in healthcare and that education, training, interpreter services and diversification of staff would improve their ability to support ethnically diverse individuals.[9] Furthermore, studies exploring the impact of cultural tailoring on diabetes management among ethnic minority populations found that tailored interventions, including language-appropriate resources and culturally sensitive advice, are needed to promote uptake and adherence.[34] The lack of cultural competency in healthcare can exacerbate existing health inequalities within ethnically diverse populations, reflecting a fundamental inequity in access and quality of care.[35] Studies have demonstrated disparities in health outcomes among minority groups, partly due to cultural insensitivity in interventions and healthcare delivery.[36-39]

Addressing this issue necessitates enhancing the cultural competency of healthcare providers, especially coaches involved in delivering health programmes. Culturally

For peer review only - http://bmjopen.bmj.com/site/about/guidelines.xhtml

competent interventions have been shown to improve engagement, adherence, and outcomes, effectively reducing health disparities.[38,39] By tailoring the NHS LCD programme to be culturally sensitive and equipping coaches with cultural competence, healthcare interventions can become more equitable, ensuring that individuals from diverse ethnic backgrounds receive the same quality of care and just opportunities, as has been demonstrated in previous T2D interventions.[40,41] Theoretically, equity in programme delivery would positively impact outcomes, such as weight loss, promoting fairness and reducing the health disparities prevalent among ethnically diverse populations.[42]

The challenges articulated by the coaches in this research suggest the need for changes at the provider level. Addressing these challenges requires support and training, empowering coaches with the necessary skills and insights to effectively support diverse ethnic populations. Training is required for coaches to enhance their cultural competency to provide the skills to effectively engage with individuals from diverse cultural backgrounds through being able to tailor resources and delivery of the programme to provide culturally relevant support. This is supported by previous studies which emphasised the significance of cultural competence for healthcare professionals[33,43,44] and reported that culturally tailored dietary advice and resources were associated with increased participant satisfaction and greater adherence to dietary recommendations.[33,44]

<u>Impact on coach delivery</u>

Coaches described their lack of awareness regarding the impact of cultural competency and tailoring of delivery. Research suggests a variety of healthcare staff particularly in the US from a White ethnic background may hold unconscious biases towards racial and ethnic minorities,[45] and may have preconceptions in regard to a person's background. This can be shaped by their personal experiences and the institutional cultures within healthcare organisations, some of which may have an element of institutional racism.[46] There is a need for enhancing cultural competence which goes beyond typical judgements of ethnicities or cultures. Rather, practitioners need an understanding not only of their own perceptions and attributions of others but also a critical self-reflection of how these perceptions are created.[46,47] This would support coaches in exploring how factors impact patients whose values and priorities may differ from their own.[47,48]

For peer review only - http://bmjopen.bmj.com/site/about/guidelines.xhtml

Therefore, the pressing need for training becomes evident, as highlighted by coaches who have identified a lack of self-knowledge concerning cultural nuances and dietary preferences, yet find they need to tailor the programme to meet the needs of the diverse population group without relevant skills and knowledge of cultural differences. The need for cultural tailoring in healthcare interventions is underscored by previous research with diverse ethnic participants undertaking the NHS LCD which emphasised the necessity for individualised advice and tailored support to address the unique needs of diverse populations, ensuring that healthcare services are effective and equitable for all individuals.[49]

Research shows that health coaches' attitudes, communication styles and ability to understand and implement cultural nuances impact patient-provider relationships in healthcare programmes.[14] There is a need to support individuals with a person centred approach whilst considering the wider determinants of health and communicating with each individual to find out the best way to support them to provide equitable care. However, as shown from the results in this paper there are difficulties in communication at times, especially where English is not the first language.

South Asian coaches in this study described the benefit of cultural matching in which HCP and participant are matched based on ethnicity. Whilst cultural matching between HCPs and participants has previously been described as helpful to allow for shared understanding, it may not always be feasible given lack of funding in healthcare, the diverse range of cultural backgrounds amongst participants and the need for competent and experienced HCPs.[50] Therefore focusing on cultural competency through training and resource provision for those delivering the programme would support in providing tailored advice which is equitable. Such training can equip healthcare professionals with the skills and insights necessary to give culturally sensitive advice to support those from diverse ethnic backgrounds. However, it is important to note an exclusive focus on culture or ethnicity can lead potentially to assumed judgements and stereotypes. This was highlighted by coaches in the lack of understanding of ethnicities, cultural differences and the need for cultural considerations in health programmes on the basis of ethnicity.[51] Instead, a person centred approach which considers social, environmental and contextual factors is required.[10]

<u>Strengths and limitations</u>

For peer review only - http://bmjopen.bmj.com/site/about/guidelines.xhtml

To our knowledge this is the first study of this kind, providing insights into coaches' experience on an LCD programme an area of research which is underrepresented within T2D.

A limitation of this study is that despite intending to do so, there was a lack of uptake and thus a lack of representation of service providers with only two out of six being represented. A further limitation includes the collective analysis of coaches, overlooking their diverse characteristics and varying backgrounds, which could impact on their experiences of delivery as there are differences in heritage, cultural practices, generational and regional differences. Additionally, the duration of coaches' experience in delivering the programme was unspecified and could impact on their experiences thus the insight of the research, therefore collecting this information in future research would be useful. Furthermore, a limitation of this research study is the insufficient exploration of the substantial variations in ethnicities and cultures, the focus was on the coaches, however, is it a limitation of the research that it was not possible to consider the nuanced, diverse needs within and between ethnic and cultural groups. The absence of in-depth discussions or data collection regarding these diverse subcategories highlights the need for further research and considerations to ensure a comprehensive understanding of how cultural nuances may impact the implementation and effectiveness of the low calorie diet.

<u>Implications for policy and practice</u>

The findings from this study have implications for NHS LCD programme delivery and future policy and practice within the healthcare sector, particularly in the context of supporting service providers in providing culturally tailored and effective approaches to diverse ethnic populations. To support more equitable healthcare interventions, the following actions are recommended:

- Cultural competency training: Incorporate comprehensive cultural competency training into the education and professional development of coaches, supporting better understanding of cultural nuances, dietary preferences, and the influence of culture on health behaviours. Coaches need to able to deliver the programme to those from different background and ethnicities, to effectively do this an understanding of cultural nuances is required.

For peer review only

- Culturally-tailored resources: Development and utilisation of culturally-tailored resources and materials for healthcare interventions, aligning with the dietary preferences and cultural backgrounds of participants. For example, signposting to existing resources and literature such as the South Asian and African and Caribbean Eatwell guide.[52]
- Language-specific groups: Develop language-specific support groups and resources within healthcare programmes to cater to participants where English is not a first language.
- Diverse workforce: Within the contextual setting the programme is being delivered to promote diversity within the workforce. Having colleagues from diverse backgrounds facilitates mutual support, encouraging the exchange of knowledge and fostering interpersonal collaboration. This diversity can enable team members to ask questions, share insights, and collectively enhance their cultural competence, ultimately ensuring more effective support for the ethnically diverse participant population.
- Further research: Is needed to examine cultural and contextual influences, the long-term effects of cultural tailoring on patient outcomes, the most effective methods for cultural competency training, and the scalability of culturally sensitive interventions within broader healthcare systems. Through exploring this in future research, programmes and practices within healthcare can be improved and a more equitable service provided.

Conclusion

In healthcare programmes, care should be person-centred, with lifestyle interventions involving culturally tailored approaches, particular in relation to dietary and lifestyle advice. There is a need to develop an understanding of different cultures and ethnicities and the factors that may contribute to inequity for different populations to support the delivery of future health programmes. By acknowledging the current limitations in training, these findings advocate for a more culturally competent approach in the education and support for health coaches, to foster more inclusive healthcare practices. Among other recommendations including culturally tailored resources and language-specific groups, coaches delivering the NHS LCD Programme would benefit from enhanced training and

For peer review only - http://bmjopen.bmj.com/site/about/guidelines.xhtml

support to equip them with the knowledge and skills necessary to tailor delivery of the programme to individuals from diverse ethnic backgrounds: reducing barriers and optimising inclusivity and effectiveness of the programme across ethnic groups.

Author statement

PD, MM, CH, KD and LE provided substantial contributions to the design of the work. PD, MM and KD provided substantial contributions to the interpretation of data for the work. All authors PD, MM, KD, CH, LE and CB drafted the work and provided revisions. All authors provided final approval of the version to be published and provided agreement to be accountable for all aspects of the work in ensuring that questions related to the accuracy or integrity of any part of the work are appropriately investigated and resolved.

Declarations

The views expressed in this paper are those of the authors and not necessarily those of the NHS or the National Institute for Health Research.

Availability of data and materials

The datasets generated during this current study are not publicly available due to reasons of privacy and confidentiality, and because of the inability to de-identify the data. Additional knowledge of the data can be available from the corresponding author on reasonable request.

Competing interests

All authors confirm that they have no conflicts of interest to declare. LE has received funding from NIHR, MRC, Leeds Council and OHID/PHE in the last 3 years, and has had an honorary contract with OHID. CB is a primary care advisor to the national diabetes programme for NHS England.

Funding

For peer review only

For peer review only - http://bmjopen.bmj.com/site/about/guidelines.xhtml

This work was supported by the National Institute for Health Research, Health Services and Delivery Research [NIHR 132075]. The NHS England Low-Calorie Diet Pilot is funded by NHS England.

Ethics approval

Ethical approval was gained from Leeds Beckett University (LBU 102077), written informed consent was obtained from participants. The Re:Mission study was approved by Health Research Authority on 5 July 2021, REC ref: 21/WM/0136.

For peer review only

References

1. NHS. Long Term Plan. Updated 19/09/2023. Accessed 03rd July, 2023. https://www.longtermplan.nhs.uk/

2. Organisation WH. Obesity and overweight. World Health Organisation. Accessed 10th December, 2022. https://www.who.int/news-room/fact-sheets/detail/obesity-and-overweight

3. Leung G, Stanner S. Diets of minority ethnic groups in the UK: influence on chronic disease risk and implications for prevention. *Nutrition Bulletin*. 2011;36(2):161-198. doi:10.1111/j.1467-3010.2011.01889.x

4. Organisation WH. Health Equity. Updated 11/10/2023. Accessed 10th November, 2022. https://www.who.int/health-topics/health-equity#tab=tab_1

5. Bleich SN, Jarlenski MP, Bell CN, LaVeist TA. Health inequalities: trends, progress, and policy. *Annu Rev Public Health*. Apr 2012;33:7-40. doi:10.1146/annurev-publhealth-031811-124658

6. Dharmi Kapadia JZ, Sarah Salway, James Nazroo, Andrew Booth, Nazmy Villarroel-Williams, Laia Bécares & Aneez Esmail. *Ethnic Inequalities in Healthcare: A Rapid Evidence Review*. 2022. *NHS Race and Health Observatory*. https://www.nhsrho.org/wp-content/uploads/2022/02/RHO-Rapid-Review-Final-Report_v.7.pdf

7. Schouten BC, Cox A, Duran G, et al. Mitigating language and cultural barriers in healthcare communication: Toward a holistic approach. *Patient Educ Couns*. May 8 2020;doi:10.1016/j.pec.2020.05.001

8. McGregor B, Belton A, Henry TL, Wrenn G, Holden KB. Improving Behavioral Health Equity through Cultural Competence Training of Health Care Providers. *Ethn Dis*. 2019;29(Suppl 2):359-364. doi:10.18865/ed.29.S2.359

9. Shepherd SM, Willis-Esqueda C, Newton D, Sivasubramaniam D, Paradies Y. The challenge of cultural competence in the workplace: perspectives of healthcare providers. *BMC Health Services Research*. 2019;19(1)doi:10.1186/s12913-019-3959-7

10. Jongen C, McCalman J, Bainbridge R. Health workforce cultural competency interventions: a systematic scoping review. *BMC Health Services Research*. 2018;18(1)doi:10.1186/s12913-018-3001-5

11. Kaihlanen A-M, Hietapakka L, Heponiemi T. Increasing cultural awareness: qualitative study of nurses' perceptions about cultural competence training. *BMC Nursing*. 2019;18(1)doi:10.1186/s12912-019-0363-x

For peer review only - http://bmjopen.bmj.com/site/about/guidelines.xhtml

12. Ojo AS, Nnyanzi LA, Giles EL, et al. “I am not really into the government telling me what I need to eat”: exploring dietary beliefs, knowledge, and practices among ethnically diverse communities in England. *BMC Public Health*. 2023;23(1):800. doi:10.1186/s12889-023-15689-6

13. Ofosu NN, Luig T, Mumtaz N, et al. Health care providers’ perspectives on challenges and opportunities of intercultural health care in diabetes and obesity management: a qualitative study. *CMAJ Open*. 2023;11(4):E765-E773. doi:10.9778/cmajo.20220222

14. Thom DH, Wolf J, Gardner H, et al. A Qualitative Study of How Health Coaches Support Patients in Making Health-Related Decisions and Behavioral Changes. *The Annals of Family Medicine*. 2016;14(6):509-516. doi:10.1370/afm.1988

15. Debesay J, Kartzow AH, Fougner M. Healthcare professionals’ encounters with ethnic minority patients: The critical incident approach. *Nursing Inquiry*. 2022;29(1)doi:10.1111/nin.12421

16. Yasui M, Pottick KJ, Chen Y. Conceptualizing Culturally Infused Engagement and Its Measurement for Ethnic Minority and Immigrant Children and Families. *Clin Child Fam Psychol Rev*. Sep 2017;20(3):250-332. doi:10.1007/s10567-017-0229-2

17. NHS. Low calorie diets to treat obesity and Type 2 diabetes. Accessed 7th March 2022, https://www.england.nhs.uk/diabetes/treatment-care/low-calorie-diets/

18. Drew KJ HC, Radley D, Freeman C, Kinsella K, Maynard M, Bakhai, C and Ells, L. . Equity and Local Health Systems – a qualitative evaluation of the experiences of Local Health Service Leads during the first two years of the NHS Low Calorie Diet Programme pilot. . *The British Journal of Diabetes*. 2023;Forthcoming.

19. Lean ME, Leslie WS, Barnes AC, et al. Primary care-led weight management for remission of type 2 diabetes (DiRECT): an open-label, cluster-randomised trial. *The Lancet*. 2018;391(10120):541-551. doi:10.1016/s0140-6736(17)33102-1

20. Astbury NM, Aveyard P, Nickless A, et al. Doctor Referral of Overweight People to Low Energy total diet replacement Treatment (DROPLET): pragmatic randomised controlled trial. *BMJ*. 2018:k3760. doi:10.1136/bmj.k3760

21. England N. Service Specification No. 1: NHS Low Calorie Diet Programme [Version 01]. 2021

22. Evans TS, Dhir P, Radley D, et al. Does the design of the NHS Low-Calorie Diet Programme have fidelity to the programme specification? A documentary review of service parameters and behaviour change content in a type 2 diabetes intervention. *Diabetic Medicine*. 2023;40(4)doi:10.1111/dme.15022

23. C Bakhai EB, T Gorton, S Safazadeh, S Jebb, P Aveyard, J Wilding, E Robertson, R Taylor, J Valabhji. The ambition for remission – the NHS Type 2 Diabetes Path to Remission Programme. DPC 20232023.

24. C Bakhai EB, T Gorton, S Safazadeh, S Jebb, P Aveyard, J Wilding, E Robertson, R Taylor, J Valabhji. EARLY FINDINGS FROM THE NHS TYPE 2 DIABETES PATH TO REMISSION PROGRAME (formerly NHS Low Calorie Diet Programme) 2023.

25. NIHR. A coproduced mixed method evaluation of the NHS England low calorie diet implementation pilot. Accessed 16 January, 2022. https://fundingawards.nihr.ac.uk/award/NIHR132075

26. Fletcher AJ. Applying critical realism in qualitative research: Methodology meets method. *International Journal of Social Research Methodology: Theory & Practice*. 2017;20(2):181-194. doi:10.1080/13645579.2016.1144401

27. Statistics OfN. (2011a). 2011 rural/urban classification. Accessed 03/10/2023, https://www.ons.gov.uk/methodology/geography/geographicalproducts/ruralurbanclassifications/2011ruralurbanclassification

28. Clarke VBaV. *Thematic Analysis: A practical guide*. SAGE; 2022.

29. Malterud K, Siersma VD, Guassora AD. Sample Size in Qualitative Interview Studies: Guided by Information Power. *Qual Health Res*. Nov 2016;26(13):1753-1760. doi:10.1177/1049732315617444

For peer review only - http://bmjopen.bmj.com/site/about/guidelines.xhtml

30. Braun V, Clarke V. To saturate or not to saturate? Questioning data saturation as a useful concept for thematic analysis and sample-size rationales. *Qualitative Research in Sport, Exercise and Health*. 2021/03/04 2021;13(2):201-216. doi:10.1080/2159676X.2019.1704846

31. Netto G, Bhopal R, Lederle N, Khatoon J, Jackson A. How can health promotion interventions be adapted for minority ethnic communities? Five principles for guiding the development of behavioural interventions. *Health Promotion International*. 2010;25(2):248-257. doi:10.1093/heapro/daq012

32. Resnicow K, Baranowski, T., Ahluwalia, J.S. and Braithwaite, R.L. Cultural Sensitivity in Public Health: Defined and Demystified. *Ethnicity and Disease*. 1999;9:10-21.

33. Joo JY, Liu MF. Culturally tailored interventions for ethnic minorities: A scoping review. *Nursing Open*. 2021;8(5):2078-2090. doi:10.1002/nop2.733

34. Litchfield I, Barrett T, Hamilton-Shield J, et al. Current evidence for designing self-management support for underserved populations: an integrative review using the example of diabetes. *International Journal for Equity in Health*. 2023/09/11 2023;22(1):188. doi:10.1186/s12939-023-01976-6

35. Bentley P, Jovanovic A, Sharma P. Cultural diversity training for UK healthcare professionals: a comprehensive nationwide cross-sectional survey. *Clin Med (Lond)*. Oct 2008;8(5):493-7. doi:10.7861/clinmedicine.8-5-493

36. Brach C, Fraser I. Can cultural competency reduce racial and ethnic health disparities? A review and conceptual model. *Med Care Res Rev*. 2000;57 Suppl 1(Suppl 1):181-217. doi:10.1177/1077558700057001s09

37. Memon A, Taylor K, Mohebati LM, et al. Perceived barriers to accessing mental health services among black and minority ethnic (BME) communities: a qualitative study in Southeast England. *BMJ Open*. 2016;6(11):e012337. doi:10.1136/bmjopen-2016-012337

38. Goff LM. Ethnicity and Type 2 diabetes in the UK. *Diabetic Medicine*. 2019;36(8):927-938. doi:https://doi.org/10.1111/dme.13895

39. Creamer J, Attridge M, Ramsden M, Cannings-John R, Hawthorne K. Culturally appropriate health education for Type 2 diabetes in ethnic minority groups: an updated Cochrane Review of randomized controlled trials. *Diabet Med*. Feb 2016;33(2):169-83. doi:10.1111/dme.12865

40. Wallia S, Bhopal RS, Douglas A, et al. Culturally adapting the prevention of diabetes and obesity in South Asians (PODOSA) trial. *Health Promot Int*. Dec 2014;29(4):768-79. doi:10.1093/heapro/dat015

41. Goff LM, Moore AP, Harding S, Rivas C. Development of Healthy Eating and Active Lifestyles for Diabetes, a culturally tailored diabetes self-management education and support programme for Black-British adults: A participatory research approach. *Diabet Med*. Nov 2021;38(11):e14594. doi:10.1111/dme.14594

42. Betancourt JR, Green AR, Carrillo JE, Ananeh-Firempong O, 2nd. Defining cultural competence: a practical framework for addressing racial/ethnic disparities in health and health care. *Public Health Rep*. Jul-Aug 2003;118(4):293-302. doi:10.1093/phr/118.4.293

43. Dragomanovich HM, Shubrook JH. Improving Cultural Humility and Competency in Diabetes Care for Primary Care Providers. *Clin Diabetes*. Apr 2021;39(2):220-224. doi:10.2337/cd20-0063

44. Pettersson S, Holstein J, Jirwe M, Jaarsma T, Klompstra L. Cultural competence in healthcare professionals, specialised in diabetes, working in primary healthcare—A descriptive study. *Health & Social Care in the Community*. 2022;30(3)doi:10.1111/hsc.13442

45. Hamed S, Bradby H, Ahlberg BM, Thapar-Björkert S. Racism in healthcare: a scoping review. *BMC Public Health*. May 16 2022;22(1):988. doi:10.1186/s12889-022-13122-y

46. Danso A, Danso Y. The complexities of race and health. *Future Healthc J*. Mar 2021;8(1):22-27. doi:10.7861/fhj.2020-0225

47. Napier D, Depledge M, Knipper M, et al. *Culture matters: using a cultural contexts of health approach to enhance policy-making*. 2017.

For peer review only - http://bmjopen.bmj.com/site/about/guidelines.xhtml

48. Tee S, Üzar-Özçetin YS, Trenoweth S. Achieving culturally competent mental health care: A mixed-methods study drawing on the perspectives of UK nursing students. *Perspect Psychiatr Care*. Oct 2022;58(4):1267-1280. doi:10.1111/ppc.12926

49. Dhir P, Maynard M, Drew KJ, Homer CV, Bakhai C, Ells LJ. South Asian individuals' experiences on the NHS low-calorie diet programme: a qualitative study in community settings in England. *BMJ Open*. 2023;13(12):e079939. doi:10.1136/bmjopen-2023-079939

50. Handtke O, Schilgen B, Mösko M. Culturally competent healthcare - A scoping review of strategies implemented in healthcare organizations and a model of culturally competent healthcare provision. *PLoS One*. 2019;14(7):e0219971. doi:10.1371/journal.pone.0219971

51. FitzGerald C, Hurst S. Implicit bias in healthcare professionals: a systematic review. *BMC Med Ethics*. Mar 1 2017;18(1):19. doi:10.1186/s12910-017-0179-8

52. Jay F. The South Asian Eatwell Guide. Accessed 11 November, 2023. https://mynutriweb.com/the-south-asian-eatwell-guide/

For peer review only

For peer review only - http://bmjopen.bmj.com/site/about/guidelines.xhtml

Consolidated criteria for reporting qualitative studies (COREQ): 32-item checklist

No. Item	Guide questions/description	Reported on Page #
**Domain 1: Research team and reflexivity**		
Personal Characteristics		
1. Interviewer/facilitator	Which author/s conducted the interview or focus group?	Page 7
2. Credentials	What were the researcher's credentials? E.g. PhD, MD	Page 1
3. Occupation	What was their occupation at the time of the study?	Page 1
4. Gender	Was the researcher male or female?	Page 7
5. Experience and training	What experience or training did the researcher have?	Page 7
Relationship with participants		
6. Relationship established	Was a relationship established prior to study commencement?	Page 6-8
7. Participant knowledge of the interviewer	What did the participants know about the researcher? e.g. personal goals, reasons for doing the research	Page 7
8. Interviewer characteristics	What characteristics were reported about the interviewer/facilitator? e.g. Bias, assumptions, reasons and interests in the research topic	Page 7
**Domain 2: study design**		
Theoretical framework		
9. Methodological orientation and Theory	What methodological orientation was stated to underpin the study? e.g. grounded theory, discourse analysis, ethnography, phenomenology, content analysis	Page 6
Participant selection		
10. Sampling	How were participants selected? e.g. purposive, convenience, consecutive, snowball	Page 7

For peer review only

11. Method of approach	How were participants approached? E.g. face-to-face, telephone, mail, email	Page 7
12. Sample size	How many participants were in the study?	Page 7
13. Non-participation	How many people refused to participate or dropped out? Reasons?	None
Setting		
14. Setting of data collection	Where was the data collected? e.g. home, clinic, workplace	Page 7-8
15. Presence of non-participants	Was anyone else present besides the participants and researchers?	Page 7
16. Description of sample	What are the important characteristics of the sample? E.g. demographic data, date	Page 7-8.
Data collection		
17. Interview guide	Were questions, prompts, guides provided by the authors? Was it pilot tested?	Page 7 and supplementary file 2.
18. Repeat interviews	Were repeat interviews carried out? If yes, how many?	No
19. Audio/visual recording	Did the research use audio or visual recording to collect the data?	Page 8
20. Field notes	Were field notes made during and/or after the interview or focus group?	Page 8
21. Duration	What was the duration of the interviews or focus group?	Page 8
22. Data saturation	Was data saturation discussed?	Page 9
23. Transcripts returned	Were transcripts returned to participants for comment and/or correction?	No
**Domain 3: analysis and findings**		
Data analysis		
24. Number of data coders	How many data coders coded the data?	Page 9
25. Description of the coding tree	Did authors provide a description of the coding tree?	Page 9 and Supplementary file 4
26. Derivation of themes	Were themes identified in advance or derived from the data?	Derived from the data.
27. Software	What software, if applicable, was used to manage the data?	Page 9
28. Participant checking	Did participants provide feedback on the findings?	No
Reporting		
29. Quotations presented	Were participant quotations presented to	Page 10-15

For peer review only

	illustrate the themes/findings? Was each quotation identified? e.g. participant number	
30. Data and findings consistent	Was there consistency between the data presented and the findings?	Page 10-19
31. Clarity of major themes	Were major themes clearly presented in the findings?	Page 15-19
32. Clarity of minor themes	Is there a description of diverse cases or discussion of minor themes?	During completion of the reflexive thematic analysis led to the amalgamation of minor themes into overarching themes, particularly as interpretations diverge from participants' accounts. In this study, this led to minor themes not being explicitly delineated as themes; instead, becoming embedded within the descriptive text used to substantiate the major theme and to discuss the overarching results.

Developed from:
Tong A, Sainsbury P, Craig J. Consolidated criteria for reporting qualitative research (COREQ): a 32-item checklist for interviews and focus groups. *International Journal for Quality in Health Care*. 2007. Volume 19, Number 6: pp. 349 – 357

Personal Characteristics:

Pooja Dhir (Female, British Indian). PhD student of 2 years and registered dietitian for 7 years.

Professor Maria Maynard (Female, Black Caribbean). Professor of Health Inequalities with extensive experience of ethnicity and health research.

Dr Kevin J Drew PhD (Male, White British). Post-doctoral Research Fellow with 7 years' experience of conducting qualitative evaluations of health-based interventions.

Dr Catherine Homer PhD (Female, White British). Senior Research Fellow with experience working in academia and extensive experience working in public health.

For peer review only

Dr Chirag Bakhai (Male, Indian). General Practitioner in primary care with a special interest in diabetes management. Chirag is a clinical lead on the Oversight group for the Re:Mission Study and a primary care advisor to the national diabetes programme for NHS England and NHS Improvement.

Dr Louisa Ells (Female, White British). Professor of Obesity with a specialist interest in multi-disciplinary, cross-sector applied obesity research, with extensive experience of leading programme evaluations.

For peer review only

Interview guide

Topic 1: Closed Background questions

What programme provider are they part of and what is your role with the provider?

About their ethnicity?

Topic 2

1. Can you tell me how well you think the programme works for minoritised populations?
   - i. Can you describe your role on the programme?
   - ii. Can you give any examples of what you think works well?
   - iii. Is there anything that doesn't work well?
   - iv. What are your thoughts on the uptake and adherence of individuals from these groups on the programme?
   - v. Do people from ethnic minority populations attend the sessions you have delivered?
   - vi. If not what do they perceive are the barriers to being in the programme?
2. What training have you received to help you deliver to ethnic populations?
   - i. How did you find the support that was provided?
   - vii. Can you tell me about any training you have had to work with people from ethnic populations?
3. How is the programme tailored to support people from ethnic groups to access, engage and adhere?
   - i. What impacts have you seen for individuals from the specific tailoring?
   - ii. Does the overall programme work for ethnic populations why / why not?
4. Can you describe what have been your experiences of delivering the programme to individuals from ethnic backgrounds?
   - i. Can you describe what has worked well?
   - ii. Can you describe any challenges?
   - iii. Can you describe what changes were made to support this population?
   - iv. How has the programme or the delivery been tailored for ethnic populations?
   - v. Were you able to resolve the barriers, and if so how?
   - vi. How have the challenges you faced impacted your delivery of the programme?
5. How do you think the programme and delivery could be improved for ethnic populations?
   - i. Can you describe your experiences of being a coach and delivering to ethnic populations - what works well / challenges / additional support needs?
6. What considerations, if any do you think have been made to support ethnic populations in completing the programme?

i. Can you describe what further considerations you think need to be made?

ii. Can you describe what you think of the considerations that have already been made?

iii. Can you tell me what improvements you think are needed to the programme to support those from ethnic populations?

Any questions?

For peer review only - http://bmjopen.bmj.com/site/about/guidelines.xhtml

Population living with type 2 diabetes.

Time	Three Phases of the Low Calorie Diet Programme
1-12 Weeks	**LCD:** with total diet replacement products (TDR) up to 900 calories per day. During this time patients will replace all normal meals with these products.

In this section demographic information on the population living with diabetes is provided, following an estimated population that meets the eligibility criteria of the Low Calorie Diet programme. Figures in Table 1 are taken from the pilot site prospectuses compiled in mid-2019. Birmingham and Solihull, and North London Partners in Health and Care referenced the National Diabetes Audit (NDA) 17-18 as the source of their figures. Gloucestershire referenced a Local Diabetes Audit 17-18, while Derbyshire referenced RAIDR 2019. The other six pilot sites do not provide a reference for where this information has been taken from.

Table 1. Population living with type 2 diabetes

Pilot Sites	People with Type 2 Diabetes	% Of Total Population
South Yorkshire and Bassetlaw	93,090	6.2%
Frimley Health and Care	29,950	3.7%
Greater Manchester HSCP	156,625	5.6%
Joined Up Care Derbyshire	50,273	5.0%
Birmingham and Solihull	74,250	5.7%
East London Health and Care Partnership	116,096	5.3%
Humber Coast and Vale	82,426	5.9%
Gloucestershire	27,309	4.2%
Bedfordshire, Luton and Milton Keynes	48,445	4.8%
North London Partners in Health and Care	69,530	4.3%
**Total:**	**747,994**	

Table 2: Overview of the LCD programme

For peer review only

12-18 Weeks	**Food re-introduction:** during this time patients will be supported to re-introduce food, with a stepped phasing out of TDR products.
18-52 Weeks	**Maintenance**, during this time patients will be supported to maintain their initial weight loss.

During the TDR phase patients are expected to replace all normal meals with TDR products, which are provided by the provider for the duration of the service. Each service provider provides different TDR products such as soups, shakes, bars, and meal replacements such as porridge and daal, which all come in different flavours.

For peer review

<u>Ethnicity</u>

The distribution of type 2 diabetes amongst different ethnic groups varies. The data in Table 3 are taken from the NDA, and so are based on the registers of patients collected and held at GP practices rather than directly from census data. Percentages may not total 100 because ethnicity has not been recorded in all cases.

<u>Table 3: Ethnicity at pilot site level</u>

**Pilot sites**	**CCGs (2019/2020)**	**% with Type 2 diabetes by:**	
		White	***Minority Ethnic***
South Yorkshire and Bassetlaw	NHS Barnsley	97.0	1.7
	NHS Bassetlaw	80.9	2.8
	NHS Doncaster	88.8	5.9
	NHS Rotherham	83.9	8.0
	NHS Sheffield	73.3	18.8
Frimley Health and Care	NHS East Berkshire	50.6	43.4
	NHS NE Hampshire and Farnham	67.6	17.6
	NHS Surrey Heath	67.5	16.2
Greater Manchester	NHS Bolton	62.9	29.4
	NHS Bury	56.5	12.7
	NHS Heywood, Middleton, Rochdale	66.3	30.6
	NHS Manchester	49.8	44.1
	NHS Oldham	60.7	35.1
	NHS Salford	75.4	10.0

		% with Type 2 diabetes by:	
	NHS Stockport	66.0	10.9
	NHS Tameside and Glossop	78.2	13.8
	NHS Trafford	64.8	20.7
	NHS Wigan Borough	86.2	4.1
Joined Up Care Derbyshire	NHS Derby and Derbyshire	79.5	9.9
Birmingham and Solihull	NHS Birmingham and Solihull	50.8	41.2
East London Health and Care Partnership	NHS Barking and Dagenham	41.8	54.9
	NHS City and Hackney	25.2	74.4
	NHS Havering	70.0	22.0
	NHS Newham	16.2	83.1
	NHS Redbridge	25.1	72.9
	NHS Tower Hamlets	20.1	79.0
	NHS Waltham Forest	31.3	63.5
Humber Coast and Vale	NHS East Riding of Yorkshire	74.7	3.9
	NHS Hull	83.5	5.5
	NHS North East Lincolnshire	84.6	11.1
	NHS North Lincolnshire	81.1	11.7
	NHS North Yorkshire	74.0	2.4
	NHS Vale of York	75.1	2.7
Gloucestershire	NHS Gloucestershire	75.5	5.6
Bedfordshire, Luton, Milton Keynes	NHS Bedfordshire	64.4	17.7
	NHS Luton	35.4	58.2
	NHS Milton Keynes	55.5	27.2
North London Partners in Health and Care	NHS North Central London	41.4	53.2

References

1. NHS Digital. (2020a) [MI] Ethnic Category Coverage. Available from <https://digital.nhs.uk/data-and-information/publications/statistical/mi-ethnic-category-coverage/current > [Accessed on 17th May 2021].

2. Office for National Statistics. (2011b). 2011 rural/urban classification. Available from <https://www.ons.gov.uk/peoplepopulationandcommunity/culturalidentity/ethnicity> [Accessed via SHAPE on 22nd June 2021].

For peer review only

Theme: Experiences of cultural tailoring.

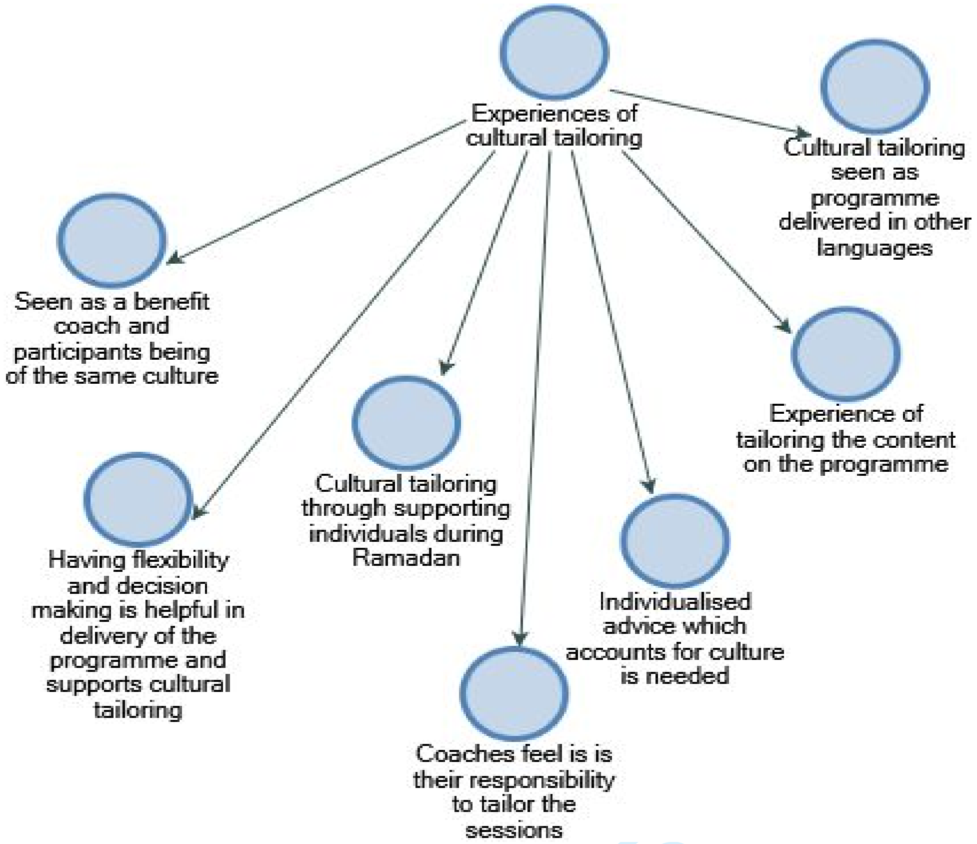

Theme: Needing to understand the impact of culture and ethnicity.

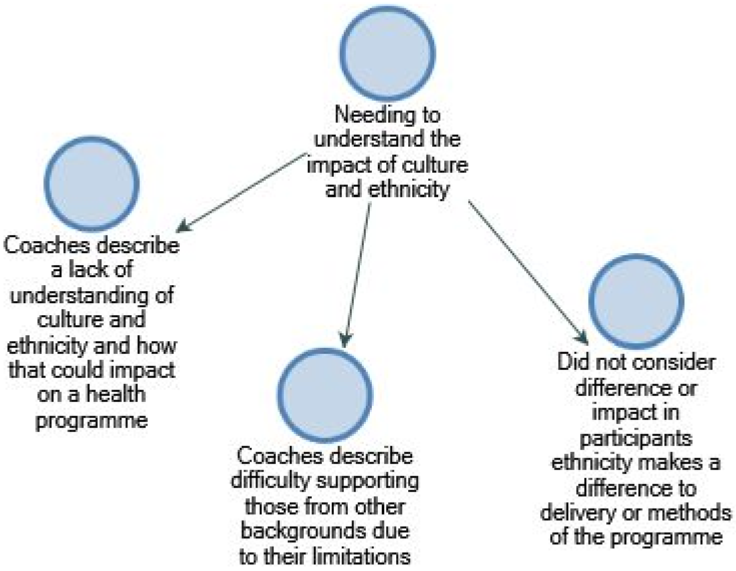

1

Theme: The impact of language.

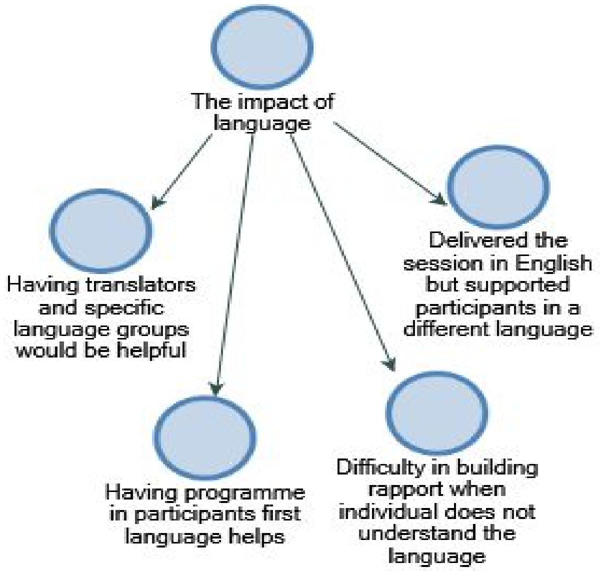

Theme: The use of resources in providing dietary advice.

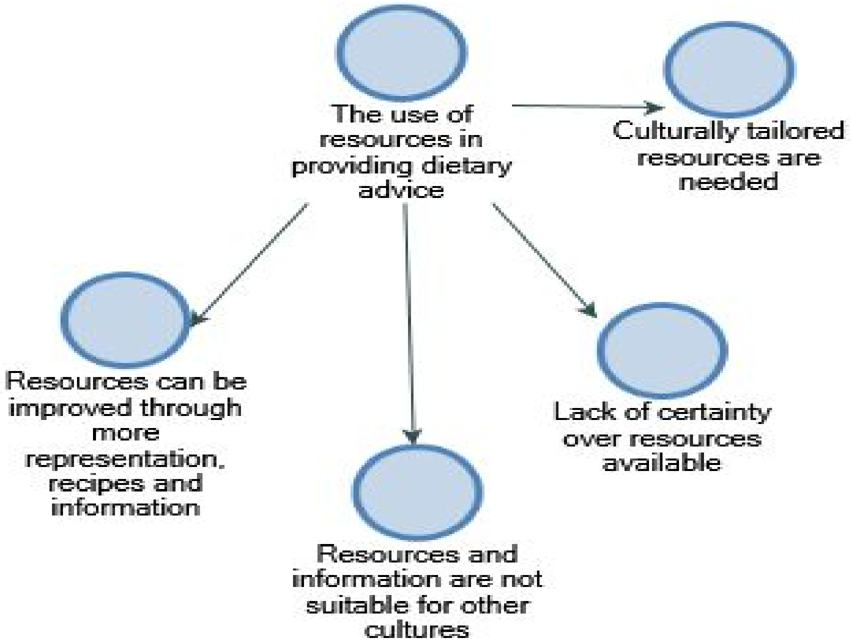

Theme: Training and support needs.

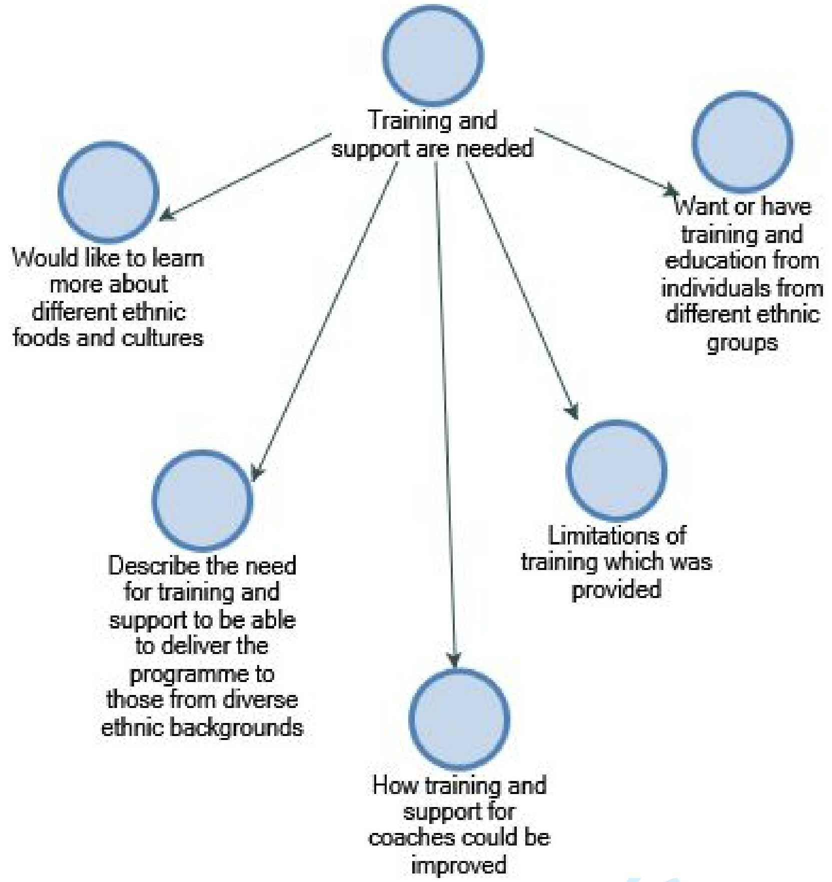

w only

For peer review only - http://bmjopen.bmj.com/site/about/guidelines.xhtml

Table to show phase 5- Refining, defining and naming themes.

Themes and Sub-themes	Interviews coded	Units of meaning coded
A mixed relationship with the coach	12	61
Coach support	11	44
Communication with coaches	5	17
More work needed in the programme for person centeredness	8	22
Describe experience of doing the programme during Ramadan	3	3
Felt religious considerations were made with TDR being halal	3	3
Found advice was geared towards a Western diet and not tailored	4	10
Lack of cultural understanding	1	1
Lack of recipes for different cultures	3	5
Culture related experiences	12	75
Experienced judgement by others in life due to weight	2	2
Family support helped in the programme	10	19
Social cultural challenges	5	9
Support	10	45
It's not the same taste	11	58
Challenge of not having traditional foods during the programme	5	5
Difficulty in TDR due to difference from having foods	2	3
Disliked the taste of the TDR products	1	3
Having similar cultural foods would have made things easier	2	2
Limited variety of TDR products	3	5
Needing to add flavour to TDR products due to lack of flavour	9	12
Programme could be improved through taste of TDR products	5	9
Taste of products was a barrier to TDR stage	11	19
Needing motivation to make changes and feeling better	11	66
Describe feeling better within themselves	2	4
Feeling more energy through the programme	5	6
Made dietary and lifestyle changes	9	38
Mental health	8	18
Social experiences	11	43
Avoided social situations during TDR phase	8	20
Challenge of managing mealtimes in eating with family and takeaways	1	2
Felt 'empty' during TDR as unable to eat with others and still cooking for others	1	3
How to manage meal times during TDR with family	4	6
Managed social situations of eating out by planning ahead in TDR	3	3
Socio-cultural experiences	4	9

For peer review only - http://bmjopen.bmj.com/site/about/guidelines.xhtml

BMJ Open

A qualitative evaluation in community settings in England exploring of the experiences of coaches delivering the NHS Low Calorie Diet Programme Pilot to ethnically diverse participants.

Journal:	*BMJ Open*
Manuscript ID	bmjopen-2024-085200.R1
Article Type:	Original research
Date Submitted by the Author:	11-Apr-2024

Complete List of Authors:	Dhir, Pooja; Leeds Beckett University<br>Maynard, Maria; Leeds Beckett University, School of Health; Leeds Beckett University, Obesity Institute<br>Drew, Kevin J; Leeds Beckett University<br>Homer, Catherine; Sheffield Hallam University, Centre for HEalth and Social Care Research<br>Bakhai, Chirag; NHS Bedfordshire Luton and Milton Keynes STP<br>Ells, Louisa; Leeds Beckett University, Obesity Institute

**Primary Subject Heading**:	Nutrition and metabolism
Secondary Subject Heading:	Diabetes and endocrinology, Public health, Health policy
Keywords:	Obesity, PUBLIC HEALTH, DIABETES & ENDOCRINOLOGY, NUTRITION & DIETETICS, General diabetes < DIABETES & ENDOCRINOLOGY, Health Education

SCHOLARONE™
Manuscripts

I, the Submitting Author has the right to grant and does grant on behalf of all authors of the Work (as defined in the below author licence), an exclusive licence and/or a non-exclusive licence for contributions from authors who are: i) UK Crown employees; ii) where BMJ has agreed a CC-BY licence shall apply, and/or iii) in accordance with the terms applicable for US Federal Government officers or employees acting as part of their official duties; on a worldwide, perpetual, irrevocable, royalty-free basis to BMJ Publishing Group Ltd ("BMJ") its licensees and where the relevant Journal is co-owned by BMJ to the co-owners of the Journal, to publish the Work in this journal and any other BMJ products and to exploit all rights, as set out in our licence.

The Submitting Author accepts and understands that any supply made under these terms is made by BMJ to the Submitting Author unless you are acting as an employee on behalf of your employer or a postgraduate student of an affiliated institution which is paying any applicable article publishing charge ("APC") for Open Access articles. Where the Submitting Author wishes to make the Work available on an Open Access basis (and intends to pay the relevant APC), the terms of reuse of such Open Access shall be governed by a Creative Commons licence – details of these licences and which Creative Commons licence will apply to this Work are set out in our licence referred to above.

Other than as permitted in any relevant BMJ Author's Self Archiving Policies, I confirm this Work has not been accepted for publication elsewhere, is not being considered for publication elsewhere and does not duplicate material already published. I confirm all authors consent to publication of this Work and authorise the granting of this licence.

For peer review only

A qualitative evaluation in community settings in England exploring the experiences of coaches delivering the NHS Low Calorie Diet Programme Pilot to ethnically diverse participants.

Authors: Pooja Dhir[1] (0000-0001-9225-0442), Maria Maynard[1] (0000-0002-0011-752X), Kevin J Drew[1] (0000-0003-0149-2521), Catherine Homer[2] (0000-0003-2571-6008), Chirag Bakhai[3] (0009-0008-4708-3305), Louisa J. Ells[1] (0000-0003-0559-4832)

Affiliations:

1 Obesity Institute, School of Health, Leeds Beckett University, Leeds, LS1 3HB, UK

2 Sport and Physical Activity Research Centre, Sheffield Hallam University, Olympic Legacy Park, 2 Old Hall Road, Sheffield S9 3TU, UK

3 NHS Bedfordshire, Luton and Milton Keynes Integrated Care Board, Arndale House, Luton, LU1 2LJ

Corresponding author: Pooja Dhir, Obesity Institute, School of Health, Leeds Beckett University CL10, Calverley Building, City Campus, Leeds, LS1 3HE.

p.dhir2675@student.leedsbeckett.ac.uk

Abstract 269 words. Word count: 5431

Conflicts of Interest: All authors confirm that they have no conflicts of interest to declare. LE has received funding from NIHR, MRC, Leeds Council and OHID/PHE in the last 3 years and has had an honorary contract with OHID.

CB is a primary care advisor to the national diabetes programme for NHS England.

Funding information: This work was supported by the National Institute for Health Research, Health Services and Delivery Research [NIHR 132075]. The NHS England Low Calorie Diet Pilot is funded by NHS England.

Contributor and guarantor information

"The corresponding author attests that all listed authors meet authorship criteria and that no others meeting the criteria have been omitted."

For peer review only

For peer review only - http://bmjopen.bmj.com/site/about/guidelines.xhtml

Transparency statement: The lead author affirms that this manuscript is an honest, accurate, and transparent account of the study being reported; that no important aspects of the study have been omitted; and that any discrepancies from the study as planned (and, if relevant, registered) have been explained.

Abstract

Background: The management of type 2 diabetes within diverse ethnic populations requires a culturally tailored approach. However, little is known about the experiences of coaches delivering interventions for type 2 diabetes (T2D), such as the NHS Low Calorie Diet (LCD) programme, to people from diverse ethnic backgrounds.

Objective: To explore the experiences of coaches delivering an NHS programme using total diet replacement approaches to individuals from diverse ethnic backgrounds, to inform the effective tailoring and equitable delivery of future interventions.

Design: Qualitative study.

Setting: Individuals delivering the NHS LCD programme.

Participants: One-to-one semi-structured interviews were conducted with seven health coaches delivering the NHS LCD programme. Inclusion criteria included participants delivering the NHS LCD programme being either from a minoritised ethnic background or delivering the programme to those from ethnic minority and White British backgrounds.

Main outcome measures: Qualitative semi-structured interviews conducted through different stages of the programme. Reflexive thematic analysis was used to analyse the transcripts.

Results: Key themes highlighted the following experiences of delivering the LCD programme: 1. Training and support needs; 2. Needing to understand culture and ethnicity; 3. The impact of language; 4. The use of resources in providing dietary advice; and 5. Experiences of cultural tailoring. The themes highlight the need to prioritise person-centered care, to integrate culturally tailored approaches and for provision of education and training to those delivering health programmes.

For peer review only - http://bmjopen.bmj.com/site/about/guidelines.xhtml

Conclusion: These findings describe the experiences of health coaches in tailoring delivery and emphasise the role of cultural competence in ensuring equitable and effective healthcare interventions for diverse populations. This learning can inform future programmes and policies aimed at promoting inclusive healthcare practices.

Strengths and limitations of the study

- The sample did not include representation from all of the service providers delivering the NHS LCD programme as some providers did not engage with the study.
- A potential limitation lies in the small sample size of seven participants; however, it is important to recognise that in qualitative research, the emphasis is placed on the depth and richness of the data rather than on statistical power.
- The study focused on addressing an important gap in the existing literature regarding the equitable delivery total diet replacement approaches to individuals from diverse ethnic backgrounds.

What is already known about this subject

Prior research has highlighted the pressing need for culturally tailored interventions in managing type 2 diabetes within diverse ethnic populations. Studies have shown that type 2 diabetes disproportionately affects certain ethnic groups, including South Asian and Black ethnicities, emphasising the need for equitable healthcare strategies. While the importance of cultural competence in healthcare delivery has been recognised, there is limited knowledge about the specific experiences of health coaches delivering interventions like England's National Health Service (NHS) low calorie diet programme to ethnically diverse individuals.

Keywords: Type 2 diabetes, obesity, South Asian, qualitative, public health, thematic analysis, ethnicity, health care professionals, coaches

Abbreviations

For peer review only - http://bmjopen.bmj.com/site/about/guidelines.xhtml

T2D- Type 2 diabetes

UK- United Kingdom

HCPs- Health Care Professionals

US- United States

NHS- National Health Service

LCD- low calorie diet

NHSE- NHS England

Introduction

The prevalence of type 2 diabetes (T2D) remains a significant global public health concern [1,2], and its management requires a multifaceted lifestyle approach that encompasses dietary modifications.[1-3] However, food is culturally significant, where it often serves as a cornerstone of traditions, identity, and social cohesion.[3] Thus, lifestyle change programmes are advised to consider the impact of culture and ethnicity, among other social factors when attempting to modify the dietary habits of individuals. With an increasingly diverse population in the UK, issues of culture and ethnicity are central to concerns over the equitable provision of healthcare services. [1, 4]

Health equity has been defined as the fair and just opportunity, irrespective of social position, to attain full health and well-being from social conditions that seek to promote and support good health.[4] The issue of equity is important because it is healthcare inequities that create, perpetuate, and exacerbate health inequalities.[5] Indeed, inequalities in health outcomes and access to healthcare are commonplace and addressing them is a public health priority.[5] For example, in response to the recognition that patients from different ethnic backgrounds may experience disparities in healthcare access and quality[6], the notion of culturally competent, person-centredness and standardised practices in the delivery of services has been developed to try and meet the need for equitable delivery of healthcare services.[7,8,9]

Cultural competence in healthcare is described as the ability of healthcare systems to deliver interventions that encompass diversity in cultures, beliefs and behaviours while tailoring the service to individuals environmental, cultural and social needs.[10] Initially seen as

For peer review only - http://bmjopen.bmj.com/site/about/guidelines.xhtml

a set of skills and knowledge that healthcare professionals (HCPs) possess to effectively engage with patients from diverse backgrounds[11], cultural competence has evolved to encompass HCPs' ability to deliver interventions. This delivery should not only accommodate diversity in beliefs and behaviours, but also the ability to tailor services to individuals' environmental, cultural, and social needs, including different food preferences.[11] Healthcare professionals need to possess cultural competences in order to address the needs of the diverse patient populations they deliver to, and thus, deliver healthcare services in a more equitable way.

Research underscores the significance of tailoring health programme advice not only in terms of language but also in culturally appropriate contexts, encompassing written support materials and verbal interactions.[12] Studies emphasise the role of culturally competent communication in healthcare settings, highlighting its positive impact on patient outcomes and satisfaction and indicate that culturally tailored interventions lead to improved health outcomes among diverse populations.[12,13] Therefore, incorporating culturally appropriate language and materials into health programmes is essential for promoting effective engagement and understanding, which can ultimately contribute to better health outcomes and patient experiences.[13]Existing research from the perspectives of patients often underscores the need for culturally relevant support, such as through cultural competency of HCPs and culturally tailored resources such as dietary resources.[14] For example, In the United States (US), health coaches have been reported to value shared characteristics with their clients which has helped to build a trusting relationship and support with decision making.[15] More generally, previous research has explored the experiences of HCP-participant interactions and highlighted the significance of coach-participant relationships in enhancing health behaviours and outcomes.[16,17] Similarities between coaches and patients, coupled with the time spent together, facilitated the establishment of a strong coach-patient relationship.[16] Within this relationship, four key coaching activities emerged: education, personal support, practical assistance, and serving as a liaison between patients and clinicians.[16] Furthermore, we have previously explored the experiences of participants from a South Asian background, who were undertaking the NHS Low Calorie Diet (LCD) programme.)[18]

This study highlighted the importance of adopting a person-centered approach in designing

For peer review only - http://bmjopen.bmj.com/site/about/guidelines.xhtml

interventions, recognising the influence of culture, motivation, and community engagement on health behaviours and outcomes and tailored and culturally appropriate interventions for managing T2D.[18]

However, limited research is available on the experiences of the healthcare service providers in the context of supporting minoritised ethnic participants within T2D management, or within low calorie diet, total diet replacement interventions in the United Kingdom (UK).[9,16,17] Furthermore, there is a dearth of evidence relating to the practical challenges, insights, and experiences of HCPs or coaches who are working to an agenda that advocates for culturally relevant support.[19] In this context, in England, the National Health Service (NHS) recently piloted a low calorie diet programme to promote remission of T2D.

This paper on coaches' perspectives accompanies our previous paper on participant experiences[18], building on previous findings from the wider re:mission study[20,21] to gain greater understanding of the ethnic inequalities identified.

The NHS Low Calorie Diet Programme

The NHS Low Calorie Diet (LCD) programme pilot was launched in 2020 (renamed the Type 2 Diabetes Path to Remission programme when rolled out nationally in June 2023). Available to adults (18-65 years) living with a BMI ≥27kg/m² (adjusted to ≥25kg/m² for Black, Asian and other ethnic groups) and T2D diagnosis within the last 6 years, the NHS LCD programme pilot was initially mobilised in 10 integrated care systems[1] (referred to hereon in as localities)[22,23] and expanded to a further 11 localities in 2022. The 52-week programme piloted across a total of 21 socio-demographically diverse localities in England, included a 12-week TDR phase which consisted of micronutrient-complete foods such as bars, shakes and soups of various flavours (estimated 800- 900kcals/day) for the first 12 weeks, followed by a 6-week stepped food reintroduction phase and weight maintenance support until programme end[24,25] with behaviour support provided through one of the three models: 1:1,

[1] Integrated care systems are partnerships between NHS bodies, local authorities, and local organisations which work together on health and care services to improve the lives of people locally.

For peer review only - http://bmjopen.bmj.com/site/about/guidelines.xhtml

group or digital. Commissioned by NHS England (NHSE), the programme was delivered by six commercial providers, utilising a predominantly non-medical workforce such as health coaches to deliver the sessions to service users.

Table 1: Overview of the LCD programme

Time	Three Phases of the Low Calorie Diet Programme
1-12 Weeks	**LCD:** with total diet replacement products (TDR)c up to 900 calories per day. During this time patients will replace all normal meals with these products.
12-18 Weeks	**Food re-introduction:** during this time patients will be supported to re-introduce food, with a stepped phasing out of TDR products.
18-52 Weeks	**Maintenance**, during this time patients will be supported to maintain their initial weight loss.

The service specification mandates the promotion of equal access and the tailoring of support through the application of equity based policy (further description of the programme has been provided elsewhere).[26,27] Preliminary (unpublished) data from the NHS LCD Programme highlights that 18% out of estimated 21% eligible people with Asian ethnicity, 8% out of 8% of those with Black ethnicity, vs 66% of those with White ethnicity were referred to the programme.[28] The preliminary data shows that individuals from a South Asian and Black ethnicity have lower programme uptake than those of White ethnicity (61% and 67% vs 72%) and lower 12-month weight loss (6.8% vs 10.3%).[28] The overall dropout rate at 12 months was 55% for those from Asian ethnicity, there was no significant differences by ethnicity in the univariate analyses and on the multivariable logistic regression.[29]

This paper, therefore, aims to explore the experiences of health coaches who deliver the NHS LCD programme to ethnically diverse populations, while identifying the presence or not, of equitable and culturally tailored care. Given the disproportionate impact of T2D on certain ethnic groups, the study bridges the significant knowledge gap regarding the challenges, successes, and nuances faced by coaches in culturally tailored diabetes management interventions, in order to inform future service development.

Methods

For peer review only - http://bmjopen.bmj.com/site/about/guidelines.xhtml

This study formed part of the large Re:Mission study evaluation of the NHS LCD programme[30]. The study received ethical approval from Leeds Beckett University (LBU 106559) and is reported using COREQ guidelines (see supplementary file 1). Employing a qualitative research design, this study centres on the subjective views of the coaches whilst recognising that these experiences will be shaped by underlying structural, cultural, and contextual factors.[31] The full research process was conducted over a period of 12 months which included recruitment, interviews, analysis and write up, with interviews being conducted in March 2023, in order to allow engagement with participants across the three stages of the programme. Data collection and data analysis were carried out by PD who identifies as a female of Indian ethnicity (details on co-authors are reported in supplementary file 1).

Materials and procedures

The interview questions broadly covered participants' experience of the programme, such as their training, challenges faced, successful strategies employed, and suggestions for programme improvement and cultural tailoring. (See Supplementary file 2 for the interview guide). Written informed consent through a completed consent form and socio-demographic information were obtained prior to the interviews, consent was re confirmed verbally at the beginning of the interview. All of the semi-structured interviews were conducted by PD via Microsoft Teams which lasted 30-60 minutes. All the interviews were audio-recorded (with permission); the interviews were transcribed verbatim by an external transcriber. There was no Patient and Public Involvement in this study.

Patient and Public involvement

There was no patient and public involvement in this study, however PPI were involved in the overall Re:Mission project.

Participants

The recruitment process involved engaging four service providers authorised by the National Health Service England (NHSE) to deliver the programme. Subsequently, service providers disseminated an email containing the participation information sheet and details about the research to health coaches. These coaches then extended invitations to potential participants, resulting in the purposive recruitment of seven health coaches through two NHSE-authorised service providers. No financial incentive was provided. Ten coaches

For peer review only - http://bmjopen.bmj.com/site/about/guidelines.xhtml

consented to be interviewed however three had to withdraw due to time constraints, the remaining were all interviewed.

The coaches worked in diverse geographical areas including Frimley, Greater Manchester, Derbyshire, Birmingham and East and North London and delivered the programme to those from ethnic minority backgrounds.[32] One of these recruited participants conducted the programme in a language spoken by South Asian minority groups. Supplementary file 3 provides background information on the programme and demographic data.

Inclusion criteria included participants delivering the NHS LCD programme being either from a minoritised ethnic background or delivering the programme to those from ethnic minority and White British backgrounds. The study proceeded with interviewing all remaining coaches who contacted us and were available to partake in the interview process. Among those recruited, self-reported ethnicity included Pakistani (n=2), Indian (n=1) and White British (n=4). Supplementary file 3 provides data on how many individuals with T2D were eligible for the programme from different demographic areas in which the pilot programme was based and also the percentage of individuals with T2D from White and minority ethnic backgrounds.

Table 2: Participant Characteristics

Coach number	Programme delivery	Programme provider	Self-reported ethnicity	Gender
1	Online group	1	Asian Pakistani	Female
4	Online group	2	Pakistani	Female
6	Online 1:1	2	British Indian	Male
2	Online 1:1	2	White British	Female
3	Online group	2	White British	Female
5	Online 1:1	2	White British	Male
7	Online group	2	White British	Male

For peer review only - http://bmjopen.bmj.com/site/about/guidelines.xhtml

Data analysis

Transcripts were transcribed by an external transcriber and checked for accuracy by one researcher (PD). NVivo (version 12) software was used to facilitate data management.

A reflexive thematic analysis, as proposed by Braun and Clarke[33], was conducted on anonymised interview data used to explore the challenges, successes and personal reflections of coaches in delivering the programme. PD re-read interview transcripts multiple times to gain familiarity with the data, whilst annotating relevant extracts and noting broader ideas that could aid the coding in subsequent stages. Themes were then generated and revised iteratively and discussed with MM who as suggested by Sparkes and Smith acted as a critical friend after each of the stages of analysis to enhance the reflexive self- awareness of interpretations. Field notes were used to enhance this reflective process, at each of the six stages of analysis. Supplementary file 4 outlines the thematic map.

This methodology enables a flexible process to look at contextually embedded phenomena, such as cultural competence, communication barriers and the impact of coaches' backgrounds on interactions. The study is grounded in a critical realist perspective, which recognises the existence of objective realities while emphasising the importance of centering the subjective experiences and perceptions of underserved populations. This approach acknowledges that while objective structures and conditions shape individuals' lived experiences, these experiences are also influenced by subjective interpretations and perspectives. Further detail on the reflexive process is highlighted in the COREQ checklist (see supplementary file 1). Malterud, Siersma, and Guassora's (2016) concept of information power can be used within reflexive thematic analysis as an alternative to data saturation as described by Braun and Clarke (2021).[34,35] This approach allows an interpretive judgement regarding study size related to the purpose and goals of the analysis.

Results

Five themes were identified, which had a focus on the effectiveness and inclusivity of the programme, these are: training and support needs, the impact of language, the use of resources in providing dietary advice, needing to understand the impact of culture and ethnicity and experiences of cultural tailoring.

For peer review only - http://bmjopen.bmj.com/site/about/guidelines.xhtml

1. Training and support needs.

Coaches highlighted the challenges experienced when supporting participants with different ethnicities to their own, in relation to cultural food preferences and considerations associated with their ethnic backgrounds. They discussed limitations in the training they received, including a lack of specific training for working with service users from different ethnic backgrounds and cultures to theirs, and a lack of education about different cultures, multi ethnic foods and their impacts on health:

'So I wouldn't say that I had training for ethnic populations.' (Asian-Pakistani, female, C1)

'*there's not, there's not been much training on the diverse cultures.*' (White- British, female, C2)

Coaches reported several areas where training and support could be improved, which included a deeper understanding of culturally specific foods, understanding the cultural influences that affect dietary choices, and any additional considerations linked to ethnicity such as festivities and social support. Practical suggestions for training included learning about food groups and common foods within cultures, training days for cultural competencies, education on barriers and influences within specific cultures, and guidance on adapting the programme content to suit participants' cultures.

'*the challenges will still be there unless I get some training on foods or how cultures might be affected by the programme.'* (White-British, female, C2)

They also wanted education and external training from dietitians and health professionals from diverse ethnic backgrounds with an understanding and experience of relevant cultures and lifestyles, to support appreciation and understanding of the nuances within ethnicities. Furthermore, coaches highlighted a desire for support from a workforce that was also ethnically diverse, which would enable peer support between colleagues from different backgrounds to respond to the needs of participants.

'*I think yeah just having good training is a good thing. I would say training from people of that background. So maybe dieticians who work, you know, are from that ethnicity or that background I think is better…*' (Asian-Pakistani, female, C1)

For peer review only - http://bmjopen.bmj.com/site/about/guidelines.xhtml

2. Needing to understand the impact of culture and ethnicity.

Coaches described a limited understanding of culture and ethnicity and how these factors could influence the effectiveness of health programmes. They described their knowledge gaps, with one participant reporting uncertainty about what is meant by ethnicity. Furthermore, there was discussion on a lack of awareness of different religions and ethnicities with one participant reporting confusion on what was meant by 'South Asian ethnicity'. Some coaches from a White British background reported difficulty in supporting participants from backgrounds different from their own, due to their limited knowledge of dietary practices and cultural nuances.

'…it is affecting my participants if I'm not fully understanding food, culture, or anything about their background really.' (White- British, Female, C2)

'*African backgrounds because they tend to have a lot of flour-based foods in their diet and the advice is not really sort of geared to give them practical examples of how they could adjust their diet to make that work. And I, you know I would, I would have to look that up myself, you know, if I was asked that*.' (White -British, Female, C3)

The gap in their understanding led to some coaches resorting to using a generic approach, with one coach believing that treating every participant the same was better and more ethical, rather than providing tailored advice regarding culture and ethnicity. However, this was regarded at times as being due to the lack of understanding on how to tailor advice and a lack of understanding of the importance of tailoring. Furthermore, some coaches highlighted that before being asked about ethnicity as part of the current study, they had not considered how participants' ethnicity could impact the delivery and methods of the programme.

'…you know I'm still ignorant to a good portion of cultures from around the world.' (White- British, male, C7)

'*I've never until I was invited for this interview thought about making a distinction between people's backgrounds and their results. So, I'll find that really difficult to answer because I never sort of looked at it in that way…*' (White- British, Female, C3)

Other coaches acknowledged that a deeper understanding of how food and culture intersect could enhance the quality of the service they provide, with some coaches reporting they consider the difference in cultures when delivering the programme and adapt the delivery according to this.

For peer review only - http://bmjopen.bmj.com/site/about/guidelines.xhtml

‘*it is affecting my participants if I'm not fully understanding food, culture, or anything about their background really*.’ (White British, female, C2)

3. The impact of language.

Coaches from a White British ethnicity described difficulty in communicating with participants due to language differences, creating a challenge in building rapport. It was mentioned that, at times, family members were involved as translators to overcome language barriers, and without this the participants would have faced challenges in understanding the programme’s content. In general, coaches’ views were that incorporating interpreters and specific language groups within the programme could increase inclusivity and improve adherence. In turn this would support an environment where individuals with a different preferred language could fully engage and benefit from the programme.

‘We need to consider other language groups so that we make it inclusive for everybody.’ (White British, female, C2)

‘…an Urdu specific group to help tailor, to help sort of support those, so I feel like that is something that that, that's good, that helps and can help sort of like reach those that might not be able to join the programme, if because they won't, might not be able to follow it or understand what's going on.’ (White-British, male, C5)

4. The use of resources in providing dietary advice.

Experiences of inadequate resources and uncertainty of resources available such as culturally tailored resources were described, particularly from coaches of White British ethnicity. This was described as leading to difficulty in providing culturally tailored guidance. Coaches of both White British and South Asian ethnicity described the resources and information as being centred around a Western diet, which created a barrier when supporting those from other cultural backgrounds.

‘a lot of the information is based on a typical Western diet.’ (White British, female, C3)

‘having like maybe different resources so you could go into a conversation with a specific resource open to know what to be talking about in certain areas might be useful.’ (Pakistani, female, C4)

For peer review only - http://bmjopen.bmj.com/site/about/guidelines.xhtml

Coaches highlighted that for resources to be effective, they needed to reflect the cultural background of the programme's participants. They suggested resources can be improved through incorporating culture-specific recipes and further representation in the information and guidance.

'The programme could benefit from a lot more diverse and wide range of recipes' (White-British, female, C2)

'Maybe making some recipes that would be just tailored towards them. That could be something that we could look at, you know, do. Maybe even and digging into how we can actually break that barrier of you know getting these service users onto the programme without having them to think, oh, these are not the foods that we regularly have so how am I going to have that while I'm sitting with my family?' (Pakistani, female, C4)

5. Experiences of cultural tailoring.

Coaches described a sense of responsibility for adapting programme sessions to align with the cultural backgrounds of participants. Commonly, cultural tailoring was perceived as delivering the content in other languages and providing support and specific resources during Ramadan. Therefore, there was a limited view of cultural tailoring and limited discussions on how different religions may affect the delivery of the NHS LCD programme. Cultural tailoring was at times perceived as providing advice to reduce the quantities of cultural foods such as chapatis.

'…it's cutting down on rice and cutting down on chapattis and things.' (White-British, male, C7)

Coaches of South Asian ethnicity reported further cultural tailoring through individualised advice and customising the content of the session, and also reported the positive impact of sharing the same ethnicity as the participants. These coaches highlighted the benefit of speaking the same language as participants and that their personal experiences and cultural understanding enabled them to relate to participants and build rapport. Furthermore, coaches discussed the importance of flexibility around decision-making as it allowed them to effectively tailor the programme content to the cultural preferences and needs of their participants. An example of this is a coach who reported removing a session about alcohol because it was not relevant to her group of Muslim women.

For peer review only - http://bmjopen.bmj.com/site/about/guidelines.xhtml

'...explaining things in a language that other people understood potentially it was quite helpful...' (British-Indian, female, C6)

Table 3: Participant quotes and themes

Quote	Theme	Participant details
So maybe just having another coach who you can kind of talk to every month whenever you have a session to discuss your group so that they kind of have an insight as well, and someone just to come and watch your group as well.	Training and support needs	C1 (Asian Pakistani, Female)
I think even just maybe having some more exposure on the food groups, I think that would be something that we could look into, you know which would help the service users.	Training and support needs	C4 (Pakistani, Female)
We took, we've got topics on Ramadan that so we send out like leaflets and information on that, but it's not a topic that I would say I'm fully competent in when you ask me the questions on that. So I don't think we've had enough training, to be honest.	Needing to understand the impact of culture and ethnicity.	C2 (White British, Female)
I'm very experienced in giving people suggestions, meal suggestions that could be, you know, better choices for diabetes management than the diet that they were having, and not so familiar with, you know, looking for alternatives in, in Asian food.	Needing to understand the impact of culture and ethnicity.	C3 (White British, Female)
What would be better is having, if people are not fluent in English, having more language, language options on the app or the recipes or the, the use of certain tools in the app.	The impact of language.	C2 (White British, Female)
providing them with that language, you know, language content, you know, a coach that could speak that language or, you know, somebody that could check in with them even if the coach, if they don't have a coach that's	The impact of language.	C4 (Pakistani, Female)

For peer review only

available for their language, just to see how things are. If there is anyone in the office that could, you know speak Urdu, Hindi, Punjabi, what other ethnic, you know Asian ethnic languages are, just to you know see the service user is in comfort when they are joining the session, they don't have any questions, they're not lost.		
Because then they might, they will feel rather than it just all being around maybe focused around sort of like a Western diet, they can then feel like it's more directed for them and they're sort of getting that knowledge that it's going to be more beneficial to them rather than, rather than something that they're not gonna really have much interest in and there only being like, slight, slight add-ons about their own diet.	The use of resources in providing dietary advice.	C5 (White British, Male)
So we have specific ideas for different cuisines and it wouldn't just be for the South Asian, but it would also have to be for African, Afro Caribbean and those are sort of the main groups that we, yeah that we are aware of.	The use of resources in providing dietary advice.	C3 (White British, Female)
So you have to convey all of the concepts and ideas and kind of all of that, of course, but it's just how you do it. So, but even things like you know, recipe ideas, we had a lot that were relevant to my group. And also kind of Indian populations as well.	Experiences of cultural tailoring	C1 (Asian Pakistani, Female)
I think people do appreciate it when you just put the time in to sort of take on what they're saying and take on, like especially cultural things because people are quite proud of, of their culture a lot of the times. So just, you know, knowing that it's being appreciated, it's being considered, you know, it's not just being brushed off and going OK, well we have this way of doing things so you're gonna abandon your culture basically to eat how we wanna tell	Experiences of cultural tailoring	C7 (White British, Male)

For peer review only - http://bmjopen.bmj.com/site/about/guidelines.xhtml

you to eat, and that's just not gonna work for anyone.		

397

398 Discussion

399 The themes highlight the varying cultural competency of coaches in their understanding of cultures and ethnicities and the potential impact of knowledge gaps such as tailoring on programme delivery. The study provides insight into delivering the LCD programme, emphasising the need for comprehensive training, addressing language barriers, utilising culturally tailored resources, understanding diverse cultures, and implementing effective cultural tailoring strategies. Despite the geographical diversity and two providers involved, consistent themes emerged across locations and among the various service providers interviewed.

407 Culturally relevant support

408 The multifaceted concept of cultural tailoring encompasses linguistic adaptations, cultural understanding, and the development of tailored resources, through the use of guiding principles for cultural tailoring and cultural competency to support this.[36] Whilst this research reveals the presence of important 'surface-level' adaptations within the NHS LCD programme such as the Urdu language group and some ethnic-specific resources such as for Ramadan, it also underscores the need for 'deeper' level adaptation, to cultivate a thorough comprehension of the cultural contexts, beliefs, and values that influence individuals' health behaviours.[37(p2-3)] 'Deep structure' adaptations are described as those which go beyond superficial adjustments and incorporate understandings of cultural underpinnings, and are more likely to result in behaviour change through aligning with frameworks for cultural tailoring such as through creating culturally relevant educational materials and incorporating traditional dietary practices which incorporate understanding of core cultural values and how a range of social, ethnic, environmental and historical beliefs can shape health beliefs and behaviours.[36-38]

422 The coaches' uncertainty about available resources and the cultural misalignment of existing materials highlights areas where improvements are required. Through enhancing representation, diversifying recipes, and providing culturally tailored information and

For peer review only - http://bmjopen.bmj.com/site/about/guidelines.xhtml

resources, programmes can better meet the needs of ethnically diverse populations. The coaches described the potential benefits of incorporating professional interpreters and establishing specific language-based support groups, ultimately working toward a more inclusive and effective programme for participants from diverse backgrounds. This finding aligns with the theme on the benefit of peer support from the participant paper.[18]

Similarly previous research has highlighted challenges of cultural competency in healthcare and that education, training, interpreter services and diversification of staff would improve their ability to support ethnically diverse individuals.[9] Furthermore, studies exploring the impact of cultural tailoring on diabetes management among ethnic minority populations found that tailored interventions, including language-appropriate resources and culturally sensitive advice, are needed to promote uptake and adherence.[39] The lack of cultural competency in healthcare can exacerbate existing health inequalities within ethnically diverse populations, reflecting a fundamental inequity in access and quality of care.[40] Studies have demonstrated disparities in health outcomes among minority groups, partly due to cultural insensitivity in interventions and healthcare delivery.[41-44]

Addressing this issue necessitates enhancing the cultural competency of healthcare providers, especially coaches involved in delivering health programmes. Culturally competent interventions have been shown to improve engagement, adherence, and outcomes, effectively reducing health disparities.[43,44] By tailoring the NHS LCD programme to be culturally sensitive and equipping coaches with cultural competence, healthcare interventions can become more equitable, ensuring that individuals from diverse ethnic backgrounds receive the same quality of care and just opportunities, as has been demonstrated in previous T2D interventions.[45,46] Theoretically, equity in programme delivery would positively impact outcomes, such as weight loss, promoting fairness and reducing the health disparities prevalent among ethnically diverse populations.[47]

Whilst the coaches did not extensively discuss the format of delivery in interviews, research suggests that despite the convenience and accessibility benefits of online healthcare programmes, ethnically diverse populations may face challenges with this delivery approach.[48] Therefore, providing culturally tailored telehealth services in patients' preferred languages or through bilingual health providers should be considered for more favourable outcomes.[48]

For peer review only - http://bmjopen.bmj.com/site/about/guidelines.xhtml

456 The challenges articulated by the coaches in this research suggest the need for changes at the provider level. Addressing these challenges requires support and training, empowering coaches with the necessary skills and insights to effectively support diverse ethnic populations. Training is required for coaches to enhance their cultural competency to provide the skills to effectively engage with individuals from diverse cultural backgrounds through being able to tailor resources and delivery of the programme to provide culturally relevant support. This is supported by previous studies which emphasised the significance of cultural competence for healthcare professionals[38,49,50] and reported that culturally tailored dietary advice and resources were associated with increased participant satisfaction and greater adherence to dietary recommendations.[38,50]

466 Impact on coach delivery

467 Coaches described their lack of awareness regarding the impact of cultural competency and tailoring of delivery. Research suggests a variety of healthcare staff particularly in the US from a White ethnic background may hold unconscious biases towards racial and ethnic minorities,[51] and may have preconceptions in regard to a person's background. This can be shaped by their personal experiences and the institutional cultures within healthcare organisations, some of which may have an element of institutional racism.[52] There is a need for enhancing cultural competence which goes beyond typical judgements of ethnicities or cultures. Rather, practitioners need an understanding not only of their own perceptions and attributions of others but also a critical self-reflection of how these perceptions are created.[52,53] This would support coaches in exploring how factors impact patients whose values and priorities may differ from their own.[53,54]

478 Therefore, the pressing need for training becomes evident, as highlighted by coaches who have identified a lack of self-knowledge concerning cultural nuances and dietary preferences, yet find they need to tailor the programme to meet the needs of the diverse population group without relevant skills and knowledge of cultural differences. The need for cultural tailoring in healthcare interventions is underscored by previous research with diverse ethnic participants undertaking the NHS LCD which emphasised the necessity for individualised advice and tailored support to address the unique needs of diverse populations, ensuring that healthcare services are effective and equitable for all individuals.[18] The study highlighted the important and complex role of the coach-participant

For peer review only - http://bmjopen.bmj.com/site/about/guidelines.xhtml

relationship: while many praised the supportive role of coaches, acknowledging their empathy, effective communication skills, and provision of resources, others encountered challenges stemming from a lack of cultural understanding.[18]

Research shows that health coaches' attitudes, communication styles and ability to understand and implement cultural nuances impact patient-provider relationships in healthcare programmes.[16] There is a need to support individuals with a person centred approach whilst considering the wider determinants of health and communicating with each individual to find out the best way to support them to provide equitable care. However, as shown from the results in this paper there are difficulties in communication at times, especially where English is not the first language.

South Asian coaches in this study described the benefit of cultural matching in which HCP and participant are matched based on ethnicity. Whilst cultural matching between HCPs and participants has previously been described as helpful to allow for shared understanding, it may not always be feasible given lack of funding in healthcare, the diverse range of cultural backgrounds amongst participants and the need for competent and experienced HCPs.[55] Therefore focusing on cultural competency through training and resource provision for those delivering the programme would support in providing tailored advice which is equitable. Such training can equip healthcare professionals with the skills and insights necessary to give culturally sensitive advice to support those from diverse ethnic backgrounds. However, it is important to note an exclusive focus on culture or ethnicity can lead potentially to assumed judgements and stereotypes. This was highlighted by coaches in the lack of understanding of ethnicities, cultural differences and the need for cultural considerations in health programmes on the basis of ethnicity.[56] Instead, a person centred approach which considers social, environmental and contextual factors is required.[10]

<u>Strengths and limitations</u>

To our knowledge this is the first study of this kind, providing insights into coaches' experience on an LCD programme an area of research which is underrepresented within T2D.

A limitation of this study is that despite intending to do so, there was a lack of uptake and thus a lack of representation of service providers with only two out of six being represented.

For peer review only

For peer review only - http://bmjopen.bmj.com/site/about/guidelines.xhtml

A further limitation includes the collective analysis of coaches, overlooking their diverse characteristics and varying backgrounds, which could impact on their experiences of delivery as there are differences in heritage, cultural practices, generational and regional differences. However, not all background information could not be collected to maintain the anonymity of participants.

Additionally, the duration of coaches' experience in delivering the programme was unspecified and there is a lack of detail regarding the ratio of coaches to the total patient ethnicity or the percentage of different ethnicities managed by each coach which could impact on their experiences, therefore collecting this information in future research would be useful. Another limitation is the lack of background information available about the coaches and service providers, raising questions about the representativeness of the sample in relation to the broader target population. Furthermore, a limitation of this research study is the insufficient exploration of the substantial variations in ethnicities and cultures, the focus was on the coaches, however, is it a limitation of the research that it was not possible to consider the nuanced, diverse needs within and between ethnic and cultural groups. The absence of in-depth discussions or data collection regarding these diverse subcategories highlights the need for further research and considerations to ensure a comprehensive understanding of how cultural nuances may impact the implementation and effectiveness of the low calorie diet.

<u>Implications for policy and practice</u>

The findings from this study have implications for NHS LCD programme delivery and future policy and practice within the healthcare sector, particularly in the context of supporting service providers in providing culturally tailored and effective approaches to diverse ethnic populations. To support more equitable healthcare interventions, the following actions are recommended:

- Cultural competency training: Incorporate comprehensive cultural competency training into the education and professional development of coaches, supporting better understanding of cultural nuances, dietary preferences, and the influence of culture on health behaviours. Coaches need to able to deliver the programme to

For peer review only - http://bmjopen.bmj.com/site/about/guidelines.xhtml

those from different background and ethnicities, to effectively do this an understanding of cultural nuances is required.

- Culturally-tailored resources: Development and utilisation of culturally-tailored resources and materials for healthcare interventions, aligning with the dietary preferences and cultural backgrounds of participants. For example, signposting to existing resources and literature such as the South Asian and African and Caribbean Eatwell guide.[57]
- Language-specific groups: Develop language-specific support groups and resources within healthcare programmes to cater to participants where English is not a first language.
- Diverse workforce: Within the contextual setting the programme is being delivered to promote diversity within the workforce. Having colleagues from diverse backgrounds facilitates mutual support, encouraging the exchange of knowledge and fostering interpersonal collaboration. This diversity can enable team members to ask questions, share insights, and collectively enhance their cultural competence, ultimately ensuring more effective support for the ethnically diverse participant population.
- Further research: Is needed to examine cultural and contextual influences, the long-term effects of cultural tailoring on patient outcomes, the most effective methods for cultural competency training, and the scalability of culturally sensitive interventions within broader healthcare systems. Through exploring this in future research, programmes and practices within healthcare can be improved and a more equitable service provided.
- Future research: Should detail the ratio of coaches to the total patient ethnicity or reflect the percentage of different ethnicities managed by each coach, which would provide valuable insights for a more nuanced understanding of programme effectiveness and impact across diverse populations.

Conclusion

In healthcare programmes, care should be person-centred, with lifestyle interventions involving culturally tailored approaches, particular in relation to dietary and lifestyle advice. There is a need to develop an understanding of different cultures and ethnicities and the

For peer review only - http://bmjopen.bmj.com/site/about/guidelines.xhtml

factors that may contribute to inequity for different populations to support the delivery of future health programmes. By acknowledging the current limitations in training, these findings advocate for a more culturally competent approach in the education and support for health coaches, to foster more inclusive healthcare practices. Among other recommendations including culturally tailored resources and language-specific groups, coaches delivering the NHS LCD Programme would benefit from enhanced training and support to equip them with the knowledge and skills necessary to tailor delivery of the programme to individuals from diverse ethnic backgrounds: reducing barriers and optimising inclusivity and effectiveness of the programme across ethnic groups.

Author statement

PD, MM, CH, KD and LE provided substantial contributions to the design of the work. PD, MM and KD provided substantial contributions to the interpretation of data for the work. All authors PD, MM, KD, CH, LE and CB drafted the work and provided revisions. All authors provided final approval of the version to be published and provided agreement to be accountable for all aspects of the work in ensuring that questions related to the accuracy or integrity of any part of the work are appropriately investigated and resolved.

Declarations

The views expressed in this paper are those of the authors and not necessarily those of the NHS or the National Institute for Health Research.

Availability of data and materials

The datasets generated during this current study are not publicly available due to reasons of privacy and confidentiality, and because of the inability to de-identify the data. Additional knowledge of the data can be available from the corresponding author on reasonable request.

Competing interests

All authors confirm that they have no conflicts of interest to declare. LE has received funding from NIHR, MRC, Leeds Council and OHID/PHE in the last 3 years, and has had an honorary

For peer review only - http://bmjopen.bmj.com/site/about/guidelines.xhtml

contract with OHID. CB is a primary care advisor to the national diabetes programme for NHS England.

Funding

This work was supported by the National Institute for Health Research, Health Services and Delivery Research [NIHR 132075]. The NHS England Low-Calorie Diet Pilot is funded by NHS England.

Ethics approval

Ethical approval was gained from Leeds Beckett University (LBU 102077), written informed consent was obtained from participants. The Re:Mission study was approved by Health Research Authority on 5 July 2021, REC ref: 21/WM/0136.

References

1. NHS. Long Term Plan. Updated 19/09/2023. Accessed 03rd July, 2023. https://www.longtermplan.nhs.uk/
2. Organisation WH. Obesity and overweight. World Health Organisation. Accessed 10th December, 2022. https://www.who.int/news-room/fact-sheets/detail/obesity-and-overweight
3. Leung G, Stanner S. Diets of minority ethnic groups in the UK: influence on chronic disease risk and implications for prevention. *Nutrition Bulletin*. 2011;36(2):161-198. doi:10.1111/j.1467-3010.2011.01889.x
4. Organisation WH. Health Equity. Updated 11/10/2023. Accessed 10th November, 2022. https://www.who.int/health-topics/health-equity#tab=tab_1
5. Bleich SN, Jarlenski MP, Bell CN, LaVeist TA. Health inequalities: trends, progress, and policy. *Annu Rev Public Health*. Apr 2012;33:7-40. doi:10.1146/annurev-publhealth-031811-124658
6. Dharmi Kapadia JZ, Sarah Salway, James Nazroo, Andrew Booth, Nazmy Villarroel-Williams, Laia Bécares & Aneez Esmail. *Ethnic Inequalities in Healthcare: A Rapid Evidence Review*. 2022. *NHS Race and Health Observatory*. https://www.nhsrho.org/wp-content/uploads/2022/02/RHO-Rapid-Review-Final-Report_v.7.pdf
7. Schouten BC, Cox A, Duran G, et al. Mitigating language and cultural barriers in healthcare communication: Toward a holistic approach. *Patient Educ Couns*. May 8 2020;doi:10.1016/j.pec.2020.05.001
8. McGregor B, Belton A, Henry TL, Wrenn G, Holden KB. Improving Behavioral Health Equity through Cultural Competence Training of Health Care Providers. *Ethn Dis*. 2019;29(Suppl 2):359-364. doi:10.18865/ed.29.S2.359

For peer review only - http://bmjopen.bmj.com/site/about/guidelines.xhtml

9. Shepherd SM, Willis-Esqueda C, Newton D, Sivasubramaniam D, Paradies Y. The challenge of cultural competence in the workplace: perspectives of healthcare providers. *BMC Health Services Research*. 2019;19(1)doi:10.1186/s12913-019-3959-7

10. Jongen C, McCalman J, Bainbridge R. Health workforce cultural competency interventions: a systematic scoping review. *BMC Health Services Research*. 2018;18(1)doi:10.1186/s12913-018-3001-5

11. Kaihlanen A-M, Hietapakka L, Heponiemi T. Increasing cultural awareness: qualitative study of nurses' perceptions about cultural competence training. *BMC Nursing*. 2019;18(1)doi:10.1186/s12912-019-0363-x

12. Saha S, Beach MC, Cooper LA. Patient Centeredness, Cultural Competence and Healthcare Quality. *Journal of the National Medical Association*. 2008;100(11):1275-1285. doi:10.1016/s0027-9684(15)31505-4

13. Harrison R, Walton M, Chauhan A, et al. What is the role of cultural competence in ethnic minority consumer engagement? An analysis in community healthcare. *International Journal for Equity in Health*. 2019;18(1)doi:10.1186/s12939-019-1104-1

14. Ojo AS, Nnyanzi LA, Giles EL, et al. "I am not really into the government telling me what I need to eat": exploring dietary beliefs, knowledge, and practices among ethnically diverse communities in England. *BMC Public Health*. 2023;23(1):800. doi:10.1186/s12889-023-15689-6

15. Ofosu NN, Luig T, Mumtaz N, et al. Health care providers' perspectives on challenges and opportunities of intercultural health care in diabetes and obesity management: a qualitative study. *CMAJ Open*. 2023;11(4):E765-E773. doi:10.9778/cmajo.20220222

16. Thom DH, Wolf J, Gardner H, et al. A Qualitative Study of How Health Coaches Support Patients in Making Health-Related Decisions and Behavioral Changes. *The Annals of Family Medicine*. 2016;14(6):509-516. doi:10.1370/afm.1988

17. Debesay J, Kartzow AH, Fougner M. Healthcare professionals' encounters with ethnic minority patients: The critical incident approach. *Nursing Inquiry*. 2022;29(1)doi:10.1111/nin.12421

18. Dhir P, Maynard M, Drew KJ, Homer CV, Bakhai C, Ells LJ. South Asian individuals' experiences on the NHS low-calorie diet programme: a qualitative study in community settings in England. *BMJ Open*. 2023;13(12):e079939. doi:10.1136/bmjopen-2023-079939

19. Yasui M, Pottick KJ, Chen Y. Conceptualizing Culturally Infused Engagement and Its Measurement for Ethnic Minority and Immigrant Children and Families. *Clin Child Fam Psychol Rev*. Sep 2017;20(3):250-332. doi:10.1007/s10567-017-0229-2

20. Jones S, Brown TJ, Watson P, et al. Commercial provider staff experiences of the NHS low calorie diet programme pilot: a qualitative exploration of key barriers and facilitators. *BMC Health Services Research*. 2024;24(1)doi:10.1186/s12913-023-10501-y

21. Homer C KK, Drew KJ, et al. A qualitative evaluation of experiences of the Total Diet Replacement phase of the NHS Low Calorie Diet Programme pilot. *British Journal of Diabetes*. 2024;24doi:10.15277/bjd.2024.435

22. NHS. Low calorie diets to treat obesity and Type 2 diabetes. Accessed 7th March 2022, https://www.england.nhs.uk/diabetes/treatment-care/low-calorie-diets/

23. Drew KJ HC, Radley D, Freeman C, Kinsella K, Maynard M, Bakhai, C and Ells, L. . Equity and Local Health Systems – a qualitative evaluation of the experiences of Local Health Service Leads during the first two years of the NHS Low Calorie Diet Programme pilot. . *The British Journal of Diabetes*. 2023;Forthcoming.

24. Lean ME, Leslie WS, Barnes AC, et al. Primary care-led weight management for remission of type 2 diabetes (DiRECT): an open-label, cluster-randomised trial. *The Lancet*. 2018;391(10120):541-551. doi:10.1016/s0140-6736(17)33102-1

25. Astbury NM, Aveyard P, Nickless A, et al. Doctor Referral of Overweight People to Low Energy total diet replacement Treatment (DROPLET): pragmatic randomised controlled trial. *BMJ*. 2018:k3760. doi:10.1136/bmj.k3760

26. England N. Service Specification No. 1: NHS Low Calorie Diet Programme [Version 01]. 2021

For peer review only - http://bmjopen.bmj.com/site/about/guidelines.xhtml

27. Evans TS, Dhir P, Radley D, et al. Does the design of the NHS Low-Calorie Diet Programme have fidelity to the programme specification? A documentary review of service parameters and behaviour change content in a type 2 diabetes intervention. *Diabetic Medicine*. 2023;40(4)doi:10.1111/dme.15022

28. C Bakhai EB, T Gorton, S Safazadeh, S Jebb, P Aveyard, J Wilding, E Robertson, R Taylor, J Valabhji. The ambition for remission – the NHS Type 2 Diabetes Path to Remission Programme. DPC 20232023.

29. C Bakhai EB, T Gorton, S Safazadeh, S Jebb, P Aveyard, J Wilding, E Robertson, R Taylor, J Valabhji. EARLY FINDINGS FROM THE NHS TYPE 2 DIABETES PATH TO REMISSION PROGRAME (formerly NHS Low Calorie Diet Programme) 2023.

30. NIHR. A coproduced mixed method evaluation of the NHS England low calorie diet implementation pilot. Accessed 16 January, 2022. https://fundingawards.nihr.ac.uk/award/NIHR132075

31. Fletcher AJ. Applying critical realism in qualitative research: Methodology meets method. *International Journal of Social Research Methodology: Theory & Practice*. 2017;20(2):181-194. doi:10.1080/13645579.2016.1144401

32. Statistics OfN. (2011a). 2011 rural/urban classification. Accessed 03/10/2023, https://www.ons.gov.uk/methodology/geography/geographicalproducts/ruralurbanclassifications/2011ruralurbanclassification

33. Clarke VBaV. *Thematic Analysis: A practical guide*. SAGE; 2022.

34. Malterud K, Siersma VD, Guassora AD. Sample Size in Qualitative Interview Studies: Guided by Information Power. *Qual Health Res*. Nov 2016;26(13):1753-1760. doi:10.1177/1049732315617444

35. Braun V, Clarke V. To saturate or not to saturate? Questioning data saturation as a useful concept for thematic analysis and sample-size rationales. *Qualitative Research in Sport, Exercise and Health*. 2021/03/04 2021;13(2):201-216. doi:10.1080/2159676X.2019.1704846

36. Netto G, Bhopal R, Lederle N, Khatoon J, Jackson A. How can health promotion interventions be adapted for minority ethnic communities? Five principles for guiding the development of behavioural interventions. *Health Promotion International*. 2010;25(2):248-257. doi:10.1093/heapro/daq012

37. Resnicow K, Baranowski, T., Ahluwalia, J.S. and Braithwaite, R.L. Cultural Sensitivity in Public Health: Defined and Demystified. *Ethnicity and Disease*. 1999;9:10-21.

38. Joo JY, Liu MF. Culturally tailored interventions for ethnic minorities: A scoping review. *Nursing Open*. 2021;8(5):2078-2090. doi:10.1002/nop2.733

39. Litchfield I, Barrett T, Hamilton-Shield J, et al. Current evidence for designing self-management support for underserved populations: an integrative review using the example of diabetes. *International Journal for Equity in Health*. 2023/09/11 2023;22(1):188. doi:10.1186/s12939-023-01976-6

40. Bentley P, Jovanovic A, Sharma P. Cultural diversity training for UK healthcare professionals: a comprehensive nationwide cross-sectional survey. *Clin Med (Lond)*. Oct 2008;8(5):493-7. doi:10.7861/clinmedicine.8-5-493

41. Brach C, Fraser I. Can cultural competency reduce racial and ethnic health disparities? A review and conceptual model. *Med Care Res Rev*. 2000;57 Suppl 1(Suppl 1):181-217. doi:10.1177/1077558700057001s09

42. Memon A, Taylor K, Mohebati LM, et al. Perceived barriers to accessing mental health services among black and minority ethnic (BME) communities: a qualitative study in Southeast England. *BMJ Open*. 2016;6(11):e012337. doi:10.1136/bmjopen-2016-012337

43. Goff LM. Ethnicity and Type 2 diabetes in the UK. *Diabetic Medicine*. 2019;36(8):927-938. doi:https://doi.org/10.1111/dme.13895

For peer review only - http://bmjopen.bmj.com/site/about/guidelines.xhtml

44. Creamer J, Attridge M, Ramsden M, Cannings-John R, Hawthorne K. Culturally appropriate health education for Type 2 diabetes in ethnic minority groups: an updated Cochrane Review of randomized controlled trials. *Diabet Med*. Feb 2016;33(2):169-83. doi:10.1111/dme.12865

45. Wallia S, Bhopal RS, Douglas A, et al. Culturally adapting the prevention of diabetes and obesity in South Asians (PODOSA) trial. *Health Promot Int*. Dec 2014;29(4):768-79. doi:10.1093/heapro/dat015

46. Goff LM, Moore AP, Harding S, Rivas C. Development of Healthy Eating and Active Lifestyles for Diabetes, a culturally tailored diabetes self-management education and support programme for Black-British adults: A participatory research approach. *Diabet Med*. Nov 2021;38(11):e14594. doi:10.1111/dme.14594

47. Betancourt JR, Green AR, Carrillo JE, Ananeh-Firempong O, 2nd. Defining cultural competence: a practical framework for addressing racial/ethnic disparities in health and health care. *Public Health Rep*. Jul-Aug 2003;118(4):293-302. doi:10.1093/phr/118.4.293

48. Truong M, Yeganeh L, Cook O, Crawford K, Wong P, Allen J. Using telehealth consultations for healthcare provision to patients from non-Indigenous racial/ethnic minorities: a systematic review. *Journal of the American Medical Informatics Association*. 2022;29(5):970-982. doi:10.1093/jamia/ocac015

49. Dragomanovich HM, Shubrook JH. Improving Cultural Humility and Competency in Diabetes Care for Primary Care Providers. *Clin Diabetes*. Apr 2021;39(2):220-224. doi:10.2337/cd20-0063

50. Pettersson S, Holstein J, Jirwe M, Jaarsma T, Klompstra L. Cultural competence in healthcare professionals, specialised in diabetes, working in primary healthcare—A descriptive study. *Health & Social Care in the Community*. 2022;30(3)doi:10.1111/hsc.13442

51. Hamed S, Bradby H, Ahlberg BM, Thapar-Björkert S. Racism in healthcare: a scoping review. *BMC Public Health*. May 16 2022;22(1):988. doi:10.1186/s12889-022-13122-y

52. Danso A, Danso Y. The complexities of race and health. *Future Healthc J*. Mar 2021;8(1):22-27. doi:10.7861/fhj.2020-0225

53. Napier D, Depledge M, Knipper M, et al. *Culture matters: using a cultural contexts of health approach to enhance policy-making*. 2017.

54. Tee S, Üzar-Özçetin YS, Trenoweth S. Achieving culturally competent mental health care: A mixed-methods study drawing on the perspectives of UK nursing students. *Perspect Psychiatr Care*. Oct 2022;58(4):1267-1280. doi:10.1111/ppc.12926

55. Handtke O, Schilgen B, Mösko M. Culturally competent healthcare - A scoping review of strategies implemented in healthcare organizations and a model of culturally competent healthcare provision. *PLoS One*. 2019;14(7):e0219971. doi:10.1371/journal.pone.0219971

56. FitzGerald C, Hurst S. Implicit bias in healthcare professionals: a systematic review. *BMC Med Ethics*. Mar 1 2017;18(1):19. doi:10.1186/s12910-017-0179-8

57. Jay F. The South Asian Eatwell Guide. Accessed 11 November, 2023. https://mynutriweb.com/the-south-asian-eatwell-guide/

For peer review only

For peer review only - http://bmjopen.bmj.com/site/about/guidelines.xhtml

Consolidated criteria for reporting qualitative studies (COREQ): 32-item checklist

No. Item	Guide questions/description	Reported on Page #
**Domain 1: Research team and reflexivity**		
Personal Characteristics		
1. Interviewer/facilitator	Which author/s conducted the interview or focus group?	Page 7
2. Credentials	What were the researcher's credentials? E.g. PhD, MD	Page 1
3. Occupation	What was their occupation at the time of the study?	Page 1
4. Gender	Was the researcher male or female?	Page 7
5. Experience and training	What experience or training did the researcher have?	Page 7
Relationship with participants		
6. Relationship established	Was a relationship established prior to study commencement?	Page 6-8
7. Participant knowledge of the interviewer	What did the participants know about the researcher? e.g. personal goals, reasons for doing the research	Page 7
8. Interviewer characteristics	What characteristics were reported about the interviewer/facilitator? e.g. Bias, assumptions, reasons and interests in the research topic	Page 7

For peer review only

**Domain 2: study design**		
Theoretical framework		
9. Methodological orientation and Theory	What methodological orientation was stated to underpin the study? e.g. grounded theory, discourse analysis, ethnography, phenomenology, content analysis	Page 6
Participant selection		
10. Sampling	How were participants selected? e.g. purposive, convenience, consecutive, snowball	Page 7
11. Method of approach	How were participants approached? E.g. face-to-face, telephone, mail, email	Page 7
12. Sample size	How many participants were in the study?	Page 7
13. Non-participation	How many people refused to participate or dropped out? Reasons?	None
Setting		
14. Setting of data collection	Where was the data collected? e.g. home, clinic, workplace	Page 7
15. Presence of non-participants	Was anyone else present besides the participants and researchers?	Page 7
16. Description of sample	What are the important characteristics of the sample? E.g. demographic data, date	Page 6-7.
Data collection		
17. Interview guide	Were questions, prompts, guides provided by the authors? Was it pilot tested?	Page 7 and supplementary file 2.
18. Repeat interviews	Were repeat interviews carried out? If yes, how many?	No
19. Audio/visual recording	Did the research use audio or visual recording to collect the data?	Page 8
20. Field notes	Were field notes made during and/or after the interview or focus group?	Page 8
21. Duration	What was the duration of the interviews or focus group?	Page 8
22. Data saturation	Was data saturation discussed?	N/A
23. Transcripts returned	Were transcripts returned to participants for comment and/or correction?	No
**Domain 3: analysis and**		

For peer review only

**findings**		
Data analysis		
24. Number of data coders	How many data coders coded the data?	Page 8
25. Description of the coding tree	Did authors provide a description of the coding tree?	Page 8. Supplementary file 3 and 4.
26. Derivation of themes	Were themes identified in advance or derived from the data?	Derived from the data.
27. Software	What software, if applicable, was used to manage the data?	Page 8
28. Participant checking	Did participants provide feedback on the findings?	No
Reporting		
29. Quotations presented	Were participant quotations presented to illustrate the themes/findings? Was each quotation identified? e.g. participant number	Page 9-12
30. Data and findings consistent	Was there consistency between the data presented and the findings?	Page 9-15
31. Clarity of major themes	Were major themes clearly presented in the findings?	Page 9-12
32. Clarity of minor themes	Is there a description of diverse cases or discussion of minor themes?	N/A

Developed from:
Tong A, Sainsbury P, Craig J. Consolidated criteria for reporting qualitative research (COREQ): a 32-item checklist for interviews and focus groups. *International Journal for Quality in Health Care*. 2007. Volume 19, Number 6: pp. 349 – 357

Personal Characteristics:

Pooja Dhir (Female, British Indian). PhD student of 2 years and registered dietitian for 7 years.

Professor Maria Maynard (Female, Black Caribbean). Professor of Health Inequalities with extensive experience of ethnicity and health research.

Dr Kevin J Drew PhD (Male, White British). Post-doctoral Research Fellow with 7 years' experience of conducting qualitative evaluations of health-based interventions.

Dr Catherine Homer PhD (Female, White British). Senior Research Fellow with experience working in academia and extensive experience working in public health.

Dr Chirag Bakhai (Male, Indian). General Practitioner in primary care with a special interest in diabetes management. Chirag is a clinical lead on the Oversight group for the Re:Mission Study

For peer review only

and a primary care advisor to the national diabetes programme for NHS England and NHS Improvement.

Dr Louisa Ells (Female, White British). Professor of Obesity with a specialist interest in multi-disciplinary, cross-sector applied obesity research, with extensive experience of leading programme evaluations.

For peer review only

Topic 1: Closed Background questions

What programme provider are they part of and what is your role with the provider ?

About their ethnicity?

Topic 2

1. Can you tell me how well you think the programme works for minoritised populations?
   i. Can you describe your role on the programme?
   ii. Can you give any examples of what you think works well?
   iii. Is there anything that doesn't work well?
   iv. What are your thoughts on the uptake and adherence of individuals from these groups on the programme?
   v. Do people from ethnic minority populations attend the sessions you have delivered?
   vi. If not what do they perceive are the barriers to being in the programme?
2. What training have you received to help you deliver to ethnic populations?
   i. How did you find the support that was provided?
   vii. Can you tell me about any training you have had to work with people from ethnic populations?
3. How is the programme tailored to support people from ethnic groups to access, engage and adhere?
   i. What impacts have you seen for individuals from the specific tailoring?
   ii. Does the overall programme work for ethnic populations why / why not?
4. Can you describe what have been your experiences of delivering the programme to individuals from ethnic backgrounds?
   i. Can you describe what has worked well?
   ii. Can you describe any challenges?
   iii. Can you describe what changes were made to support this population?
   iv. How has the programme or the delivery been tailored for ethnic populations?
   v. Were you able to resolve the barriers, and if so how?
   vi. How have the challenges you faced impacted your delivery of the programme?
5. How do you think the programme and delivery could be improved for ethnic populations?
   i. Can you describe your experiences of being a coach and delivering to ethnic populations - what works well / challenges / additional support needs?
6. What considerations, if any do you think have been made to support ethnic populations in completing the programme?

i. Can you describe what further considerations you think need to be made?
ii. Can you describe what you think of the considerations that have already been made?
iii. Can you tell me what improvements you think are needed to the programme to support those from ethnic populations?

Any questions?

Population living with type 2 diabetes.

In this section demographic information on the population living with diabetes is provided, following an estimated population that meet the eligibility criteria of the Low Calorie Diet programme. Figures in Table 1 are taken from the pilot site prospectuses compiled in mid-2019. Birmingham and Solihull, and North London Partners in Health and Care referenced the national Diabetes Audit (NDA) 17-18 as the source of their figures. Gloucestershire reference a Local Diabetes Audit 17-18, while Derbyshire referenced RAIDR 2019. The other six pilot sites do not provide a reference for where this information has been taken from.

Table 1. Population living with type 2 diabetes

Pilot Sites	People with Type 2 Diabetes	% Of Total Population
South Yorkshire and Bassetlaw	93,090	6.2%
Frimley Health and Care	29,950	3.7%
Greater Manchester HSCP	156,625	5.6%
Joined Up Care Derbyshire	50,273	5.0%
Birmingham and Solihull	74,250	5.7%
East London Health and Care Partnership	116,096	5.3%
Humber Coast and Vale	82,426	5.9%
Gloucestershire	27,309	4.2%
Bedfordshire, Luton and Milton Keynes	48,445	4.8%
North London Partners in Health and Care	69,530	4.3%
**Total:**	**747,994**	

For peer review only

Table 2: Overview of the LCD programme

Time	Three Phases of the Low Calorie Diet Programme
1-12 Weeks	**LCD:** with total diet replacement products (TDR) up to 900 calories per day. During this time patients will replace all normal meals with these products.
12-18 Weeks	**Food re-introduction:** during this time patients will be supported to re-introduce food, with a stepped phasing out of TDR products.
18-52 Weeks	**Maintenance**, during this time patients will be supported to maintain their initial weight loss.

During the TDR phase patients are expected to replace all normal meals with TDR products, which are provided by the provider for the duration of the service. Each service provider provides different TDR products such as soups, shakes, bars, and meal replacements such as porridge and daal, which all come in different flavours.

Ethnicity

The distribution of type 2 diabetes amongst different ethnic groups varies. The data in Table 3 are taken from the NDA, and so are based on the registers of patients collected and held at GP practices rather than directly from census data. Percentages may not total 100 because ethnicity has not been recorded in all cases.

Table 3: Ethnicity at pilot site level

Pilot sites	CCGs (2019/2020)	% with Type 2 diabetes by:	
		White	***Minority Ethnic***
South Yorkshire and Bassetlaw	NHS Barnsley	97.0	1.7
	NHS Bassetlaw	80.9	2.8
	NHS Doncaster	88.8	5.9
	NHS Rotherham	83.9	8.0
	NHS Sheffield	73.3	18.8
Frimley Health and Care	NHS East Berkshire	50.6	43.4
	NHS NE Hampshire and Farnham	67.6	17.6
	NHS Surrey Heath	67.5	16.2

For peer review only

Pilot sites	CCGs (2019/2020)	% with Type 2 diabetes by:	
		White	***Minority Ethnic***
Greater Manchester	NHS Bolton	62.9	29.4
	NHS Bury	56.5	12.7
	NHS Heywood, Middleton, Rochdale	66.3	30.6
	NHS Manchester	49.8	44.1
	NHS Oldham	60.7	35.1
	NHS Salford	75.4	10.0
	NHS Stockport	66.0	10.9
	NHS Tameside and Glossop	78.2	13.8
	NHS Trafford	64.8	20.7
	NHS Wigan Borough	86.2	4.1
Joined Up Care Derbyshire	NHS Derby and Derbyshire	79.5	9.9
Birmingham and Solihull	NHS Birmingham and Solihull	50.8	41.2
East London Health and Care Partnership	NHS Barking and Dagenham	41.8	54.9
	NHS City and Hackney	25.2	74.4
	NHS Havering	70.0	22.0
	NHS Newham	16.2	83.1
	NHS Redbridge	25.1	72.9
	NHS Tower Hamlets	20.1	79.0
	NHS Waltham Forest	31.3	63.5
Humber Coast and Vale	NHS East Riding of Yorkshire	74.7	3.9
	NHS Hull	83.5	5.5
	NHS North East Lincolnshire	84.6	11.1
	NHS North Lincolnshire	81.1	11.7
	NHS North Yorkshire	74.0	2.4
	NHS Vale of York	75.1	2.7
Gloucestershire	NHS Gloucestershire	75.5	5.6
Bedfordshire, Luton, Milton Keynes	NHS Bedfordshire	64.4	17.7
	NHS Luton	35.4	58.2
	NHS Milton Keynes	55.5	27.2
North London Partners in Health and Care	NHS North Central London	41.4	53.2

References

1. NHS Digital. (2020a) [MI] Ethnic Category Coverage. Available from <https://digital.nhs.uk/data-and-information/publications/statistical/mi-ethnic-category-coverage/current > [Accessed on 17th May 2021].
2. Office for National Statistics. (2011b). 2011 rural/urban classification. Available from <https://www.ons.gov.uk/peoplepopulationandcommunity/culturalidentity/ethnicity> [Accessed via SHAPE on 22nd June 2021].

For peer review only

Theme: Experiences of cultural tailoring.

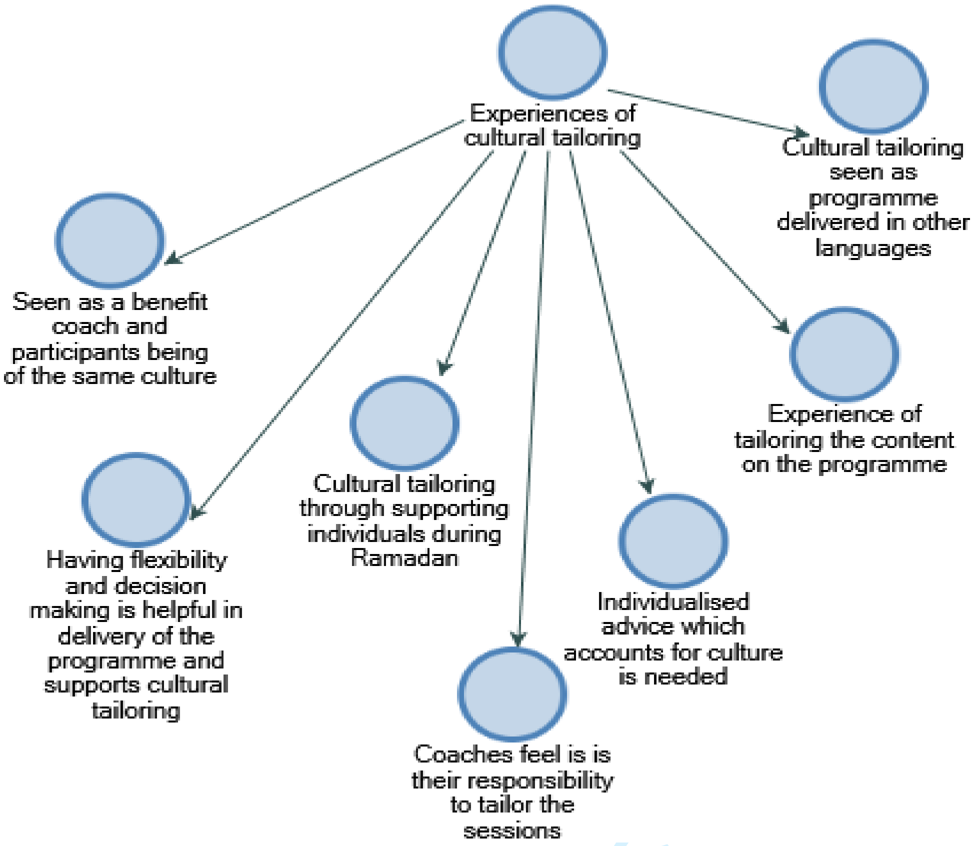

Theme: Needing to understand the impact of culture and ethnicity.

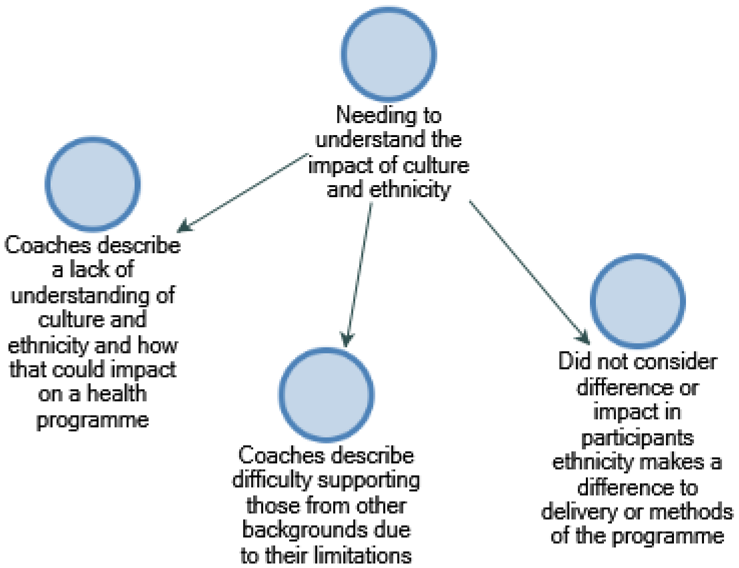

Theme: The impact of language.

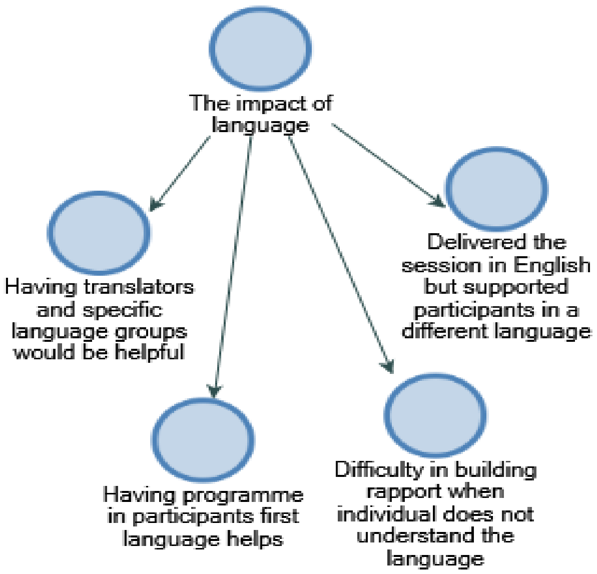

Theme: The use of resources in providing dietary advice.

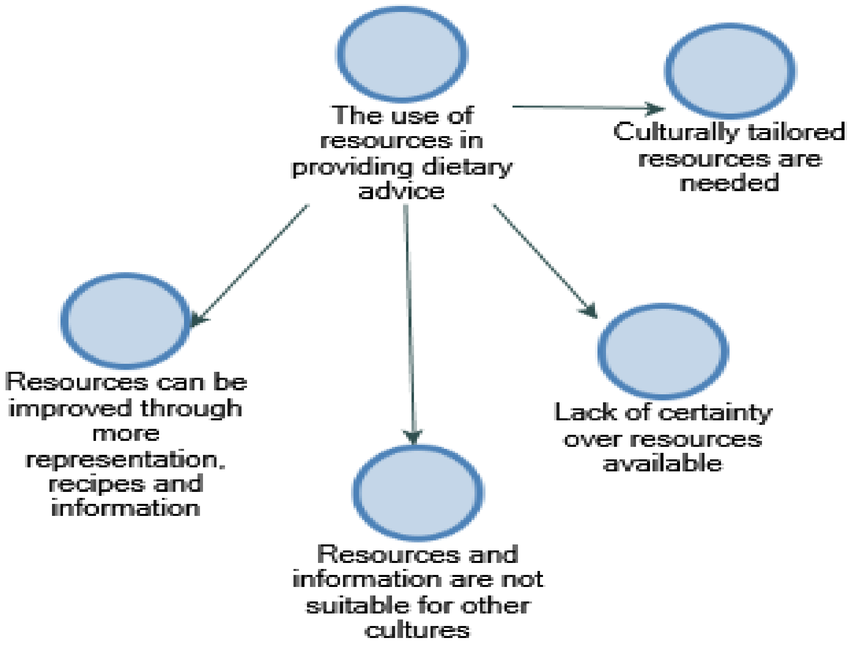

Theme: Training and support needs.

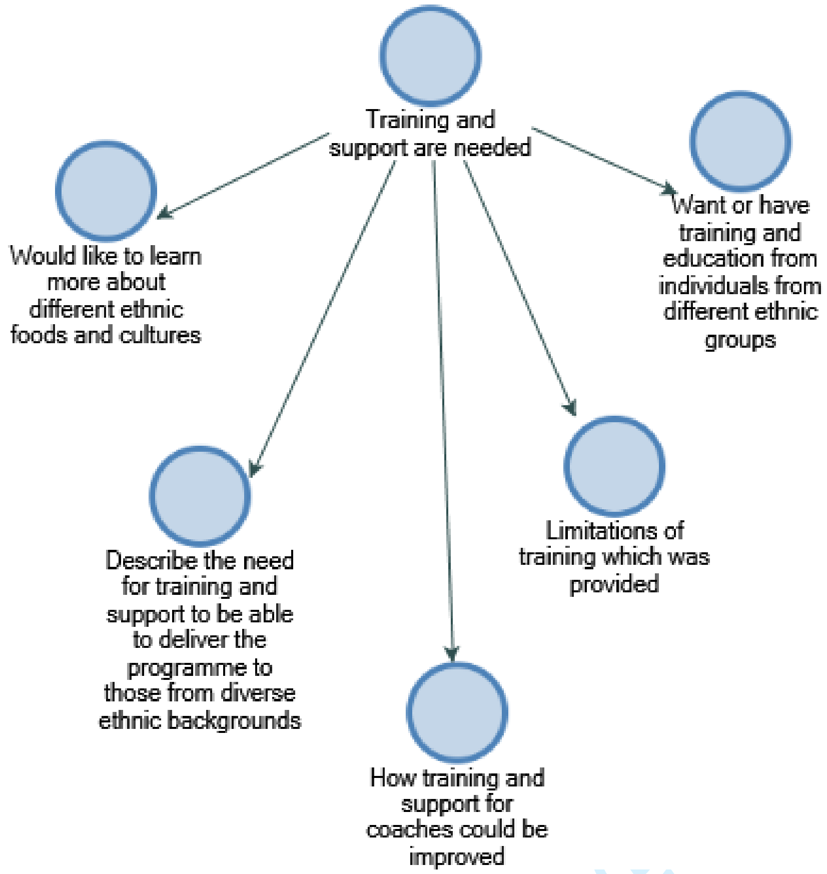

BMJ Open

A qualitative evaluation in community settings in England exploring the experiences of coaches delivering the NHS Low Calorie Diet Programme Pilot to ethnically diverse participants.

Journal:	*BMJ Open*
Manuscript ID	bmjopen-2024-085200.R2
Article Type:	Original research
Date Submitted by the Author:	30-Apr-2024

Complete List of Authors:	Dhir, Pooja; Leeds Beckett University<br>Maynard, Maria; Leeds Beckett University, School of Health; Leeds Beckett University, Obesity Institute<br>Drew, Kevin J; Leeds Beckett University<br>Homer, Catherine; Sheffield Hallam University, Centre for HEalth and Social Care Research<br>Bakhai, Chirag; NHS Bedfordshire Luton and Milton Keynes STP<br>Ells, Louisa; Leeds Beckett University, Obesity Institute

**Primary Subject Heading**:	Nutrition and metabolism
Secondary Subject Heading:	Diabetes and endocrinology, Public health, Health policy
Keywords:	Obesity, PUBLIC HEALTH, DIABETES & ENDOCRINOLOGY, NUTRITION & DIETETICS, General diabetes < DIABETES & ENDOCRINOLOGY, Health Education

SCHOLARONE™
Manuscripts

I, the Submitting Author has the right to grant and does grant on behalf of all authors of the Work (as defined in the below author licence), an exclusive licence and/or a non-exclusive licence for contributions from authors who are: i) UK Crown employees; ii) where BMJ has agreed a CC-BY licence shall apply, and/or iii) in accordance with the terms applicable for US Federal Government officers or employees acting as part of their official duties; on a worldwide, perpetual, irrevocable, royalty-free basis to BMJ Publishing Group Ltd ("BMJ") its licensees and where the relevant Journal is co-owned by BMJ to the co-owners of the Journal, to publish the Work in this journal and any other BMJ products and to exploit all rights, as set out in our licence.

The Submitting Author accepts and understands that any supply made under these terms is made by BMJ to the Submitting Author unless you are acting as an employee on behalf of your employer or a postgraduate student of an affiliated institution which is paying any applicable article publishing charge ("APC") for Open Access articles. Where the Submitting Author wishes to make the Work available on an Open Access basis (and intends to pay the relevant APC), the terms of reuse of such Open Access shall be governed by a Creative Commons licence – details of these licences and which Creative Commons licence will apply to this Work are set out in our licence referred to above.

Other than as permitted in any relevant BMJ Author's Self Archiving Policies, I confirm this Work has not been accepted for publication elsewhere, is not being considered for publication elsewhere and does not duplicate material already published. I confirm all authors consent to publication of this Work and authorise the granting of this licence.

For peer review only

A qualitative evaluation in community settings in England exploring the experiences of coaches delivering the NHS Low Calorie Diet Programme Pilot to ethnically diverse participants.

Authors: Pooja Dhir[1] (0000-0001-9225-0442), Maria Maynard[1] (0000-0002-0011-752X), Kevin J Drew[1] (0000-0003-0149-2521), Catherine Homer[2] (0000-0003-2571-6008), Chirag Bakhai[3] (0009-0008-4708-3305), Louisa J. Ells[1] (0000-0003-0559-4832)

Affiliations:

1 Obesity Institute, School of Health, Leeds Beckett University, Leeds, LS1 3HB, UK

2 Sport and Physical Activity Research Centre, Sheffield Hallam University, Olympic Legacy Park, 2 Old Hall Road, Sheffield S9 3TU, UK

3 NHS Bedfordshire, Luton and Milton Keynes Integrated Care Board, Arndale House, Luton, LU1 2LJ

Corresponding author: Pooja Dhir, Obesity Institute, School of Health, Leeds Beckett University CL10, Calverley Building, City Campus, Leeds, LS1 3HE.

p.dhir2675@student.leedsbeckett.ac.uk

Abstract 269 words. Word count: 5431

Conflicts of Interest: All authors confirm that they have no conflicts of interest to declare. LE has received funding from NIHR, MRC, Leeds Council and OHID/PHE in the last 3 years and has had an honorary contract with OHID.

CB is a primary care advisor to the national diabetes programme for NHS England.

Funding information: This work was supported by the National Institute for Health Research, Health Services and Delivery Research [NIHR 132075]. The NHS England Low Calorie Diet Pilot is funded by NHS England.

<u>Contributor and guarantor information</u>

"The corresponding author attests that all listed authors meet authorship criteria and that no others meeting the criteria have been omitted."

For peer review only - http://bmjopen.bmj.com/site/about/guidelines.xhtml

Transparency statement: The lead author affirms that this manuscript is an honest, accurate, and transparent account of the study being reported; that no important aspects of the study have been omitted; and that any discrepancies from the study as planned (and, if relevant, registered) have been explained.

Abstract

Background: The management of type 2 diabetes within diverse ethnic populations requires a culturally tailored approach. However, little is known about the experiences of coaches delivering interventions for type 2 diabetes (T2D), such as the NHS Low Calorie Diet (LCD) programme, to people from diverse ethnic backgrounds.

Objective: To explore the experiences of coaches delivering an NHS programme using total diet replacement approaches to individuals from diverse ethnic backgrounds, to inform the effective tailoring and equitable delivery of future interventions.

Design: Qualitative study.

Setting: Individuals delivering the NHS LCD programme.

Participants: One-to-one semi-structured interviews were conducted with seven health coaches delivering the NHS LCD programme. Inclusion criteria included participants delivering the NHS LCD programme being either from a minoritised ethnic background or delivering the programme to those from ethnic minority and White British backgrounds.

Main outcome measures: Qualitative semi-structured interviews conducted through different stages of the programme. Reflexive thematic analysis was used to analyse the transcripts.

Results: Key themes highlighted the following experiences of delivering the LCD programme: 1. Training and support needs; 2. Needing to understand culture and ethnicity; 3. The impact of language; 4. The use of resources in providing dietary advice; and 5. Experiences of cultural tailoring. The themes highlight the need to prioritise person-centered care, to integrate culturally tailored approaches and for provision of education and training to those delivering health programmes.

For peer review only - http://bmjopen.bmj.com/site/about/guidelines.xhtml

Conclusion: These findings describe the experiences of health coaches in tailoring delivery and emphasise the role of cultural competence in ensuring equitable and effective healthcare interventions for diverse populations. This learning can inform future programmes and policies aimed at promoting inclusive healthcare practices.

Strengths and limitations of the study

- The sample did not include representation from all of the service providers delivering the NHS LCD programme as some providers did not engage with the study.
- A potential limitation lies in the small sample size of seven participants; however, it is important to recognise that in qualitative research, the emphasis is placed on the depth and richness of the data rather than on statistical power.
- A strength of this study is its utilisation of one-to-one semi-structured interviews, allowing for an in-depth exploration of the experiences of health coaches delivering the NHS LCD programme to individuals from diverse ethnic backgrounds.

Keywords: Type 2 diabetes, obesity, South Asian, qualitative, public health, thematic analysis, ethnicity, health care professionals, coaches

Abbreviations

T2D- Type 2 diabetes

UK- United Kingdom

HCPs- Health Care Professionals

US- United States

NHS- National Health Service

LCD- low calorie diet

NHSE- NHS England

Introduction

For peer review only - http://bmjopen.bmj.com/site/about/guidelines.xhtml

The prevalence of type 2 diabetes (T2D) remains a significant global public health concern [1,2], and its management requires a multifaceted lifestyle approach that encompasses dietary modifications.[1-3] However, food is culturally significant, where it often serves as a cornerstone of traditions, identity, and social cohesion.[3] Thus, lifestyle change programmes are advised to consider the impact of culture and ethnicity, among other social factors when attempting to modify the dietary habits of individuals. With an increasingly diverse population in the UK, issues of culture and ethnicity are central to concerns over the equitable provision of healthcare services. [1, 4]

Health equity has been defined as the fair and just opportunity, irrespective of social position, to attain full health and well-being from social conditions that seek to promote and support good health.[4] The issue of equity is important because it is healthcare inequities that create, perpetuate, and exacerbate health inequalities.[5] Indeed, inequalities in health outcomes and access to healthcare are commonplace and addressing them is a public health priority.[5] For example, in response to the recognition that patients from different ethnic backgrounds may experience disparities in healthcare access and quality[6], the notion of culturally competent, person-centredness and standardised practices in the delivery of services has been developed to try and meet the need for equitable delivery of healthcare services.[7,8,9]

Cultural competence in healthcare is described as the ability of healthcare systems to deliver interventions that encompass diversity in cultures, beliefs and behaviours while tailoring the service to individuals environmental, cultural and social needs.[10] Initially seen as a set of skills and knowledge that healthcare professionals (HCPs) possess to effectively engage with patients from diverse backgrounds[11], cultural competence has evolved to encompass HCPs' ability to deliver interventions. This delivery should not only accommodate diversity in beliefs and behaviours, but also the ability to tailor services to individuals' environmental, cultural, and social needs, including different food preferences.[11] Healthcare professionals need to possess cultural competences in order to address the needs of the diverse patient populations they deliver to, and thus, deliver healthcare services in a more equitable way.

Research underscores the significance of tailoring health programme advice not only in terms of language but also in culturally appropriate contexts, encompassing written

For peer review only - http://bmjopen.bmj.com/site/about/guidelines.xhtml

support materials and verbal interactions.[12] Studies emphasise the role of culturally competent communication in healthcare settings, highlighting its positive impact on patient outcomes and satisfaction and indicate that culturally tailored interventions lead to improved health outcomes among diverse populations.[12,13] Therefore, incorporating culturally appropriate language and materials into health programmes is essential for promoting effective engagement and understanding, which can ultimately contribute to better health outcomes and patient experiences.[13]Existing research from the perspectives of patients often underscores the need for culturally relevant support, such as through cultural competency of HCPs and culturally tailored resources such as dietary resources.[14] For example, In the United States (US), health coaches have been reported to value shared characteristics with their clients which has helped to build a trusting relationship and support with decision making.[15] More generally, previous research has explored the experiences of HCP-participant interactions and highlighted the significance of coach-participant relationships in enhancing health behaviours and outcomes.[16,17] Similarities between coaches and patients, coupled with the time spent together, facilitated the establishment of a strong coach-patient relationship.[16] Within this relationship, four key coaching activities emerged: education, personal support, practical assistance, and serving as a liaison between patients and clinicians.[16] Furthermore, we have previously explored the experiences of participants from a South Asian background, who were undertaking the NHS Low Calorie Diet (LCD) programme.)[18]

This study highlighted the importance of adopting a person-centered approach in designing interventions, recognising the influence of culture, motivation, and community engagement on health behaviours and outcomes and tailored and culturally appropriate interventions for managing T2D.[18]

However, limited research is available on the experiences of the healthcare service providers in the context of supporting minoritised ethnic participants within T2D management, or within low calorie diet, total diet replacement interventions in the United Kingdom (UK).[9,16,17] Furthermore, there is a dearth of evidence relating to the practical challenges, insights, and experiences of HCPs or coaches who are working to an agenda that advocates for culturally relevant support.[19] In this context, in England, the National Health Service (NHS) recently piloted a low calorie diet programme to promote remission of T2D.

For peer review only - http://bmjopen.bmj.com/site/about/guidelines.xhtml

This paper on coaches' perspectives accompanies our previous paper on participant experiences[18], building on previous findings from the wider re:mission study[20,21] to gain greater understanding of the ethnic inequalities identified.

Time	Three Phases of the Low Calorie Diet Programme
1-12 Weeks	**LCD:** with total diet replacement products (TDR)c up to 900 calories per day. During this time patients will replace all normal meals with these products.
12-18 Weeks	**Food re-introduction:** during this time patients will be supported to re-introduce food, with a stepped phasing out of TDR products.
18-52 Weeks	**Maintenance**, during this time patients will be supported to maintain their initial weight loss.

The NHS Low Calorie Diet Programme

The NHS Low Calorie Diet (LCD) programme pilot was launched in 2020 (renamed the Type 2 Diabetes Path to Remission programme when rolled out nationally in June 2023). Available to adults (18-65 years) living with a BMI ≥27kg/m² (adjusted to ≥25kg/m² for Black, Asian and other ethnic groups) and T2D diagnosis within the last 6 years, the NHS LCD programme pilot was initially mobilised in 10 integrated care systems[1] (referred to hereon in as localities)[22,23] and expanded to a further 11 localities in 2022. The 52-week programme piloted across a total of 21 socio-demographically diverse localities in England, included a 12-week TDR phase which consisted of micronutrient-complete foods such as bars, shakes and soups of various flavours (estimated 800- 900kcals/day) for the first 12 weeks, followed by a 6-week stepped food reintroduction phase and weight maintenance support until programme end[24,25] with behaviour support provided through one of the three models: 1:1, group or digital. Commissioned by NHS England (NHSE), the programme was delivered by six commercial providers, utilising a predominantly non-medical workforce such as health coaches to deliver the sessions to service users.

Table 1: Overview of the LCD programme

[1] Integrated care systems are partnerships between NHS bodies, local authorities, and local organisations which work together on health and care services to improve the lives of people locally.

For peer review only - http://bmjopen.bmj.com/site/about/guidelines.xhtml

The service specification mandates the promotion of equal access and the tailoring of support through the application of equity based policy (further description of the programme has been provided elsewhere).[26,27] Preliminary (unpublished) data from the NHS LCD Programme highlights that 18% out of estimated 21% eligible people with Asian ethnicity, 8% out of 8% of those with Black ethnicity, vs 66% of those with White ethnicity were referred to the programme.[28] The preliminary data shows that individuals from a South Asian and Black ethnicity have lower programme uptake than those of White ethnicity (61% and 67% vs 72%) and lower 12-month weight loss (6.8% vs 10.3%).[28] The overall dropout rate at 12 months was 55% for those from Asian ethnicity, there was no significant differences by ethnicity in the univariate analyses and on the multivariable logistic regression.[29]

This paper, therefore, aims to explore the experiences of health coaches who deliver the NHS LCD programme to ethnically diverse populations, while identifying the presence or not, of equitable and culturally tailored care. Given the disproportionate impact of T2D on certain ethnic groups, the study bridges the significant knowledge gap regarding the challenges, successes, and nuances faced by coaches in culturally tailored diabetes management interventions, in order to inform future service development.

<u>Methods</u>

This study formed part of the large Re:Mission study evaluation of the NHS LCD programme[30]. The study received ethical approval from Leeds Beckett University (LBU 106559) and is reported using COREQ guidelines (see supplementary file 1). Employing a qualitative research design, this study centres on the subjective views of the coaches whilst recognising that these experiences will be shaped by underlying structural, cultural, and contextual factors.[31] The full research process was conducted over a period of 12 months which included recruitment, interviews, analysis and write up, with interviews being conducted in March 2023, in order to allow engagement with participants across the three stages of the programme. Data collection and data analysis were carried out by PD who identifies as a female of Indian ethnicity (details on co-authors are reported in supplementary file 1).

<u>Materials and procedures</u>

For peer review only - http://bmjopen.bmj.com/site/about/guidelines.xhtml

The interview questions broadly covered participants' experience of the programme, such as their training, challenges faced, successful strategies employed, and suggestions for programme improvement and cultural tailoring. (See Supplementary file 2 for the interview guide). Written informed consent through a completed consent form and socio-demographic information were obtained prior to the interviews, consent was re confirmed verbally at the beginning of the interview. All of the semi-structured interviews were conducted by PD via Microsoft Teams which lasted 30-60 minutes. All the interviews were audio-recorded (with permission); the interviews were transcribed verbatim by an external transcriber. There was no Patient and Public Involvement in this study.

Patient and Public involvement

There was no patient and public involvement in this study, however PPI were involved in the overall Re:Mission project.

Participants

The recruitment process involved engaging four service providers authorised by the National Health Service England (NHSE) to deliver the programme. Subsequently, service providers disseminated an email containing the participation information sheet and details about the research to health coaches. These coaches then extended invitations to potential participants, resulting in the purposive recruitment of seven health coaches through two NHSE-authorised service providers. No financial incentive was provided. Ten coaches consented to be interviewed however three had to withdraw due to time constraints, the remaining were all interviewed.

The coaches worked in diverse geographical areas including Frimley, Greater Manchester, Derbyshire, Birmingham and East and North London and delivered the programme to those from ethnic minority backgrounds.[32] One of these recruited participants conducted the programme in a language spoken by South Asian minority groups. Supplementary file 3 provides background information on the programme and demographic data.

Inclusion criteria included participants delivering the NHS LCD programme being either from a minoritised ethnic background or delivering the programme to those from ethnic minority and White British backgrounds. The study proceeded with interviewing all remaining coaches who contacted us and were available to partake in the interview process. Among those

For peer review only - http://bmjopen.bmj.com/site/about/guidelines.xhtml

recruited, self-reported ethnicity included Pakistani (n=2), Indian (n=1) and White British (n=4). Supplementary file 3 provides data on how many individuals with T2D were eligible for the programme from different demographic areas in which the pilot programme was based and also the percentage of individuals with T2D from White and minority ethnic backgrounds.

Table 2: Participant Characteristics

Coach number	Programme delivery	Programme provider	Self-reported ethnicity	Gender
1	Online group	1	Asian Pakistani	Female
4	Online group	2	Pakistani	Female
6	Online 1:1	2	British Indian	Male
2	Online 1:1	2	White British	Female
3	Online group	2	White British	Female
5	Online 1:1	2	White British	Male
7	Online group	2	White British	Male

Data analysis

Transcripts were transcribed by an external transcriber and checked for accuracy by one researcher (PD). NVivo (version 12) software was used to facilitate data management.

A reflexive thematic analysis, as proposed by Braun and Clarke[33], was conducted on anonymised interview data used to explore the challenges, successes and personal reflections of coaches in delivering the programme. PD re-read interview transcripts multiple times to gain familiarity with the data, whilst annotating relevant extracts and noting broader ideas that could aid the coding in subsequent stages. Themes were then generated and revised iteratively and discussed with MM who as suggested by Sparkes and Smith acted as a critical friend after each of the stages of analysis to enhance the reflexive

For peer review only - http://bmjopen.bmj.com/site/about/guidelines.xhtml

self- awareness of interpretations. Field notes were used to enhance this reflective process, at each of the six stages of analysis. Supplementary file 4 outlines the thematic map.

This methodology enables a flexible process to look at contextually embedded phenomena, such as cultural competence, communication barriers and the impact of coaches' backgrounds on interactions. The study is grounded in a critical realist perspective, which recognises the existence of objective realities while emphasising the importance of centering the subjective experiences and perceptions of underserved populations. This approach acknowledges that while objective structures and conditions shape individuals' lived experiences, these experiences are also influenced by subjective interpretations and perspectives. Further detail on the reflexive process is highlighted in the COREQ checklist (see supplementary file 1). Malterud, Siersma, and Guassora's (2016) concept of information power can be used within reflexive thematic analysis as an alternative to data saturation as described by Braun and Clarke (2021).[34,35] This approach allows an interpretive judgement regarding study size related to the purpose and goals of the analysis.

<u>Results</u>

Five themes were identified, which had a focus on the effectiveness and inclusivity of the programme, these are: training and support needs, the impact of language, the use of resources in providing dietary advice, needing to understand the impact of culture and ethnicity and experiences of cultural tailoring.

1. Training and support needs.

Coaches highlighted the challenges experienced when supporting participants with different ethnicities to their own, in relation to cultural food preferences and considerations associated with their ethnic backgrounds. They discussed limitations in the training they received, including a lack of specific training for working with service users from different ethnic backgrounds and cultures to theirs, and a lack of education about different cultures, multi ethnic foods and their impacts on health:

'So I wouldn't say that I had training for ethnic populations.' (Asian-Pakistani, female, C1)

For peer review only

‘*there's not, there's not been much training on the diverse cultures.*’ (White- British, female, C2)

Coaches reported several areas where training and support could be improved, which included a deeper understanding of culturally specific foods, understanding the cultural influences that affect dietary choices, and any additional considerations linked to ethnicity such as festivities and social support. Practical suggestions for training included learning about food groups and common foods within cultures, training days for cultural competencies, education on barriers and influences within specific cultures, and guidance on adapting the programme content to suit participants’ cultures.

‘*the challenges will still be there unless I get some training on foods or how cultures might be affected by the programme.’* (White-British, female, C2)

They also wanted education and external training from dietitians and health professionals from diverse ethnic backgrounds with an understanding and experience of relevant cultures and lifestyles, to support appreciation and understanding of the nuances within ethnicities. Furthermore, coaches highlighted a desire for support from a workforce that was also ethnically diverse, which would enable peer support between colleagues from different backgrounds to respond to the needs of participants.

‘*I think yeah just having good training is a good thing. I would say training from people of that background. So maybe dieticians who work, you know, are from that ethnicity or that background I think is better…*’ (Asian-Pakistani, female, C1)

2. Needing to understand the impact of culture and ethnicity.

Coaches described a limited understanding of culture and ethnicity and how these factors could influence the effectiveness of health programmes. They described their knowledge gaps, with one participant reporting uncertainty about what is meant by ethnicity. Furthermore, there was discussion on a lack of awareness of different religions and ethnicities with one participant reporting confusion on what was meant by ‘South Asian ethnicity’. Some coaches from a White British background reported difficulty in supporting participants from backgrounds different from their own, due to their limited knowledge of dietary practices and cultural nuances.

For peer review only - http://bmjopen.bmj.com/site/about/guidelines.xhtml

'…it is affecting my participants if I'm not fully understanding food, culture, or anything about their background really.' (White- British, Female, C2)

'African backgrounds because they tend to have a lot of flour-based foods in their diet and the advice is not really sort of geared to give them practical examples of how they could adjust their diet to make that work. And I, you know I would, I would have to look that up myself, you know, if I was asked that.' (White -British, Female, C3)

The gap in their understanding led to some coaches resorting to using a generic approach, with one coach believing that treating every participant the same was better and more ethical, rather than providing tailored advice regarding culture and ethnicity. However, this was regarded at times as being due to the lack of understanding on how to tailor advice and a lack of understanding of the importance of tailoring. Furthermore, some coaches highlighted that before being asked about ethnicity as part of the current study, they had not considered how participants' ethnicity could impact the delivery and methods of the programme.

'…you know I'm still ignorant to a good portion of cultures from around the world.' (White- British, male, C7)

'I've never until I was invited for this interview thought about making a distinction between people's backgrounds and their results. So, I'll find that really difficult to answer because I never sort of looked at it in that way…' (White- British, Female, C3)

Other coaches acknowledged that a deeper understanding of how food and culture intersect could enhance the quality of the service they provide, with some coaches reporting they consider the difference in cultures when delivering the programme and adapt the delivery according to this.

'it is affecting my participants if I'm not fully understanding food, culture, or anything about their background really.' (White British, female, C2)

3. The impact of language.

Coaches from a White British ethnicity described difficulty in communicating with participants due to language differences, creating a challenge in building rapport. It was mentioned that, at times, family members were involved as translators to overcome language barriers, and without this the participants would have faced challenges in understanding the programme's content. In general, coaches' views were that incorporating

For peer review only - http://bmjopen.bmj.com/site/about/guidelines.xhtml

interpreters and specific language groups within the programme could increase inclusivity and improve adherence. In turn this would support an environment where individuals with a different preferred language could fully engage and benefit from the programme.

'We need to consider other language groups so that we make it inclusive for everybody.' (White British, female, C2)

'…an Urdu specific group to help tailor, to help sort of support those, so I feel like that is something that that, that's good, that helps and can help sort of like reach those that might not be able to join the programme, if because they won't, might not be able to follow it or understand what's going on.' (White-British, male, C5)

4. The use of resources in providing dietary advice.

Experiences of inadequate resources and uncertainty of resources available such as culturally tailored resources were described, particularly from coaches of White British ethnicity. This was described as leading to difficulty in providing culturally tailored guidance. Coaches of both White British and South Asian ethnicity described the resources and information as being centred around a Western diet, which created a barrier when supporting those from other cultural backgrounds.

'a lot of the information is based on a typical Western diet.' (White British, female, C3)

'having like maybe different resources so you could go into a conversation with a specific resource open to know what to be talking about in certain areas might be useful.' (Pakistani, female, C4)

Coaches highlighted that for resources to be effective, they needed to reflect the cultural background of the programme's participants. They suggested resources can be improved through incorporating culture-specific recipes and further representation in the information and guidance.

'The programme could benefit from a lot more diverse and wide range of recipes' (White-British, female, C2)

'Maybe making some recipes that would be just tailored towards them. That could be something that we could look at, you know, do. Maybe even and digging into how we can actually break that barrier of you know getting these service users onto the programme

For peer review only

without having them to think, oh, these are not the foods that we regularly have so how am I going to have that while I'm sitting with my family?' (Pakistani, female, C4)

5. Experiences of cultural tailoring.

Coaches described a sense of responsibility for adapting programme sessions to align with the cultural backgrounds of participants. Commonly, cultural tailoring was perceived as delivering the content in other languages and providing support and specific resources during Ramadan. Therefore, there was a limited view of cultural tailoring and limited discussions on how different religions may affect the delivery of the NHS LCD programme. Cultural tailoring was at times perceived as providing advice to reduce the quantities of cultural foods such as chapatis.

'…it's cutting down on rice and cutting down on chapattis and things.' (White-British, male, C7)

Coaches of South Asian ethnicity reported further cultural tailoring through individualised advice and customising the content of the session, and also reported the positive impact of sharing the same ethnicity as the participants. These coaches highlighted the benefit of speaking the same language as participants and that their personal experiences and cultural understanding enabled them to relate to participants and build rapport. Furthermore, coaches discussed the importance of flexibility around decision-making as it allowed them to effectively tailor the programme content to the cultural preferences and needs of their participants. An example of this is a coach who reported removing a session about alcohol because it was not relevant to her group of Muslim women.

'…explaining things in a language that other people understood potentially it was quite helpful…' (British-Indian, female, C6)

Table 3: Participant quotes and themes

Quote	Theme	Participant details

For peer review only - http://bmjopen.bmj.com/site/about/guidelines.xhtml

So maybe just having another coach who you can kind of talk to every month whenever you have a session to discuss your group so that they kind of have an insight as well, and someone just to come and watch your group as well.	Training and support needs	C1 (Asian Pakistani, Female)
I think even just maybe having some more exposure on the food groups, I think that would be something that we could look into, you know which would help the service users.	Training and support needs	C4 (Pakistani, Female)
We took, we've got topics on Ramadan that so we send out like leaflets and information on that, but it's not a topic that I would say I'm fully competent in when you ask me the questions on that. So I don't think we've had enough training, to be honest.	Needing to understand the impact of culture and ethnicity.	C2 (White British, Female)
I'm very experienced in giving people suggestions, meal suggestions that could be, you know, better choices for diabetes management than the diet that they were having, and not so familiar with, you know, looking for alternatives in, in Asian food.	Needing to understand the impact of culture and ethnicity.	C3 (White British, Female)
What would be better is having, if people are not fluent in English, having more language, language options on the app or the recipes or the, the use of certain tools in the app.	The impact of language.	C2 (White British, Female)
providing them with that language, you know, language content, you know, a coach that could speak that language or, you know, somebody that could check in with them even if the coach, if they don't have a coach that's available for their language, just to see how things are. If there is anyone in the office that could, you know speak Urdu, Hindi, Punjabi, what other ethnic, you know Asian ethnic languages are, just to you know see the service user is in comfort when they are joining the session, they don't have any questions, they're not lost.	The impact of language.	C4 (Pakistani, Female)

For peer review only - http://bmjopen.bmj.com/site/about/guidelines.xhtml

Because then they might, they will feel rather than it just all being around maybe focused around sort of like a Western diet, they can then feel like it's more directed for them and they're sort of getting that knowledge that it's going to be more beneficial to them rather than, rather than something that they're not gonna really have much interest in and there only being like, slight, slight add-ons about their own diet.	The use of resources in providing dietary advice.	C5 (White British, Male)
So we have specific ideas for different cuisines and it wouldn't just be for the South Asian, but it would also have to be for African, Afro Caribbean and those are sort of the main groups that we, yeah that we are aware of.	The use of resources in providing dietary advice.	C3 (White British, Female)
So you have to convey all of the concepts and ideas and kind of all of that, of course, but it's just how you do it. So, but even things like you know, recipe ideas, we had a lot that were relevant to my group. And also kind of Indian populations as well.	Experiences of cultural tailoring	C1 (Asian Pakistani, Female)
I think people do appreciate it when you just put the time in to sort of take on what they're saying and take on, like especially cultural things because people are quite proud of, of their culture a lot of the times. So just, you know, knowing that it's being appreciated, it's being considered, you know, it's not just being brushed off and going OK, well we have this way of doing things so you're gonna abandon your culture basically to eat how we wanna tell you to eat, and that's just not gonna work for anyone.	Experiences of cultural tailoring	C7 (White British, Male)

Discussion

For peer review only - http://bmjopen.bmj.com/site/about/guidelines.xhtml

The themes highlight the varying cultural competency of coaches in their understanding of cultures and ethnicities and the potential impact of knowledge gaps such as tailoring on programme delivery. The study provides insight into delivering the LCD programme, emphasising the need for comprehensive training, addressing language barriers, utilising culturally tailored resources, understanding diverse cultures, and implementing effective cultural tailoring strategies. Despite the geographical diversity and two providers involved, consistent themes emerged across locations and among the various service providers interviewed.

Culturally relevant support

The multifaceted concept of cultural tailoring encompasses linguistic adaptations, cultural understanding, and the development of tailored resources, through the use of guiding principles for cultural tailoring and cultural competency to support this.[36] Whilst this research reveals the presence of important 'surface-level' adaptations within the NHS LCD programme such as the Urdu language group and some ethnic-specific resources such as for Ramadan, it also underscores the need for 'deeper' level adaptation, to cultivate a thorough comprehension of the cultural contexts, beliefs, and values that influence individuals' health behaviours.[37(p2-3)] 'Deep structure' adaptations are described as those which go beyond superficial adjustments and incorporate understandings of cultural underpinnings, and are more likely to result in behaviour change through aligning with frameworks for cultural tailoring such as through creating culturally relevant educational materials and incorporating traditional dietary practices which incorporate understanding of core cultural values and how a range of social, ethnic, environmental and historical beliefs can shape health beliefs and behaviours.[36-38]

The coaches' uncertainty about available resources and the cultural misalignment of existing materials highlights areas where improvements are required. Through enhancing representation, diversifying recipes, and providing culturally tailored information and resources, programmes can better meet the needs of ethnically diverse populations. The coaches described the potential benefits of incorporating professional interpreters and establishing specific language-based support groups, ultimately working toward a more inclusive and effective programme for participants from diverse backgrounds. This finding aligns with the theme on the benefit of peer support from the participant paper.[18]

For peer review only - http://bmjopen.bmj.com/site/about/guidelines.xhtml

Similarly previous research has highlighted challenges of cultural competency in healthcare and that education, training, interpreter services and diversification of staff would improve their ability to support ethnically diverse individuals.[9] Furthermore, studies exploring the impact of cultural tailoring on diabetes management among ethnic minority populations found that tailored interventions, including language-appropriate resources and culturally sensitive advice, are needed to promote uptake and adherence.[39] The lack of cultural competency in healthcare can exacerbate existing health inequalities within ethnically diverse populations, reflecting a fundamental inequity in access and quality of care.[40] Studies have demonstrated disparities in health outcomes among minority groups, partly due to cultural insensitivity in interventions and healthcare delivery.[41-44]

Addressing this issue necessitates enhancing the cultural competency of healthcare providers, especially coaches involved in delivering health programmes. Culturally competent interventions have been shown to improve engagement, adherence, and outcomes, effectively reducing health disparities.[43,44] By tailoring the NHS LCD programme to be culturally sensitive and equipping coaches with cultural competence, healthcare interventions can become more equitable, ensuring that individuals from diverse ethnic backgrounds receive the same quality of care and just opportunities, as has been demonstrated in previous T2D interventions.[45,46] Theoretically, equity in programme delivery would positively impact outcomes, such as weight loss, promoting fairness and reducing the health disparities prevalent among ethnically diverse populations.[47]

Whilst the coaches did not extensively discuss the format of delivery in interviews, research suggests that despite the convenience and accessibility benefits of online healthcare programmes, ethnically diverse populations may face challenges with this delivery approach.[48] Therefore, providing culturally tailored telehealth services in patients' preferred languages or through bilingual health providers should be considered for more favourable outcomes.[48]

The challenges articulated by the coaches in this research suggest the need for changes at the provider level. Addressing these challenges requires support and training, empowering coaches with the necessary skills and insights to effectively support diverse ethnic populations. Training is required for coaches to enhance their cultural competency to provide the skills to effectively engage with individuals from diverse cultural backgrounds

For peer review only - http://bmjopen.bmj.com/site/about/guidelines.xhtml

through being able to tailor resources and delivery of the programme to provide culturally relevant support. This is supported by previous studies which emphasised the significance of cultural competence for healthcare professionals[38,49,50] and reported that culturally tailored dietary advice and resources were associated with increased participant satisfaction and greater adherence to dietary recommendations.[38,50]

Impact on coach delivery

Coaches described their lack of awareness regarding the impact of cultural competency and tailoring of delivery. Research suggests a variety of healthcare staff particularly in the US from a White ethnic background may hold unconscious biases towards racial and ethnic minorities,[51] and may have preconceptions in regard to a person's background. This can be shaped by their personal experiences and the institutional cultures within healthcare organisations, some of which may have an element of institutional racism.[52] There is a need for enhancing cultural competence which goes beyond typical judgements of ethnicities or cultures. Rather, practitioners need an understanding not only of their own perceptions and attributions of others but also a critical self-reflection of how these perceptions are created.[52,53] This would support coaches in exploring how factors impact patients whose values and priorities may differ from their own.[53,54]

Therefore, the pressing need for training becomes evident, as highlighted by coaches who have identified a lack of self-knowledge concerning cultural nuances and dietary preferences, yet find they need to tailor the programme to meet the needs of the diverse population group without relevant skills and knowledge of cultural differences. The need for cultural tailoring in healthcare interventions is underscored by previous research with diverse ethnic participants undertaking the NHS LCD which emphasised the necessity for individualised advice and tailored support to address the unique needs of diverse populations, ensuring that healthcare services are effective and equitable for all individuals.[18] The study highlighted the important and complex role of the coach-participant relationship: while many praised the supportive role of coaches, acknowledging their empathy, effective communication skills, and provision of resources, others encountered challenges stemming from a lack of cultural understanding.[18]

For peer review only - http://bmjopen.bmj.com/site/about/guidelines.xhtml

Research shows that health coaches' attitudes, communication styles and ability to understand and implement cultural nuances impact patient-provider relationships in healthcare programmes.[16] There is a need to support individuals with a person centred approach whilst considering the wider determinants of health and communicating with each individual to find out the best way to support them to provide equitable care. However, as shown from the results in this paper there are difficulties in communication at times, especially where English is not the first language.

South Asian coaches in this study described the benefit of cultural matching in which HCP and participant are matched based on ethnicity. Whilst cultural matching between HCPs and participants has previously been described as helpful to allow for shared understanding, it may not always be feasible given lack of funding in healthcare, the diverse range of cultural backgrounds amongst participants and the need for competent and experienced HCPs.[55] Therefore focusing on cultural competency through training and resource provision for those delivering the programme would support in providing tailored advice which is equitable. Such training can equip healthcare professionals with the skills and insights necessary to give culturally sensitive advice to support those from diverse ethnic backgrounds. However, it is important to note an exclusive focus on culture or ethnicity can lead potentially to assumed judgements and stereotypes. This was highlighted by coaches in the lack of understanding of ethnicities, cultural differences and the need for cultural considerations in health programmes on the basis of ethnicity.[56] Instead, a person centred approach which considers social, environmental and contextual factors is required.[10]

Strengths and limitations

To our knowledge this is the first study of this kind, providing insights into coaches' experience on an LCD programme an area of research which is underrepresented within T2D.

A limitation of this study is that despite intending to do so, there was a lack of uptake and thus a lack of representation of service providers with only two out of six being represented. A further limitation includes the collective analysis of coaches, overlooking their diverse characteristics and varying backgrounds, which could impact on their experiences of delivery as there are differences in heritage, cultural practices, generational and regional

For peer review only - http://bmjopen.bmj.com/site/about/guidelines.xhtml

differences. However, not all background information could not be collected to maintain the anonymity of participants.

Additionally, the duration of coaches' experience in delivering the programme was unspecified and there is a lack of detail regarding the ratio of coaches to the total patient ethnicity or the percentage of different ethnicities managed by each coach which could impact on their experiences, therefore collecting this information in future research would be useful. Another limitation is the lack of background information available about the coaches and service providers, raising questions about the representativeness of the sample in relation to the broader target population. Furthermore, a limitation of this research study is the insufficient exploration of the substantial variations in ethnicities and cultures, the focus was on the coaches, however, is it a limitation of the research that it was not possible to consider the nuanced, diverse needs within and between ethnic and cultural groups. The absence of in-depth discussions or data collection regarding these diverse subcategories highlights the need for further research and considerations to ensure a comprehensive understanding of how cultural nuances may impact the implementation and effectiveness of the low calorie diet.

Implications for policy and practice

The findings from this study have implications for NHS LCD programme delivery and future policy and practice within the healthcare sector, particularly in the context of supporting service providers in providing culturally tailored and effective approaches to diverse ethnic populations. To support more equitable healthcare interventions, the following actions are recommended:

- Cultural competency training: Incorporate comprehensive cultural competency training into the education and professional development of coaches, supporting better understanding of cultural nuances, dietary preferences, and the influence of culture on health behaviours. Coaches need to able to deliver the programme to those from different background and ethnicities, to effectively do this an understanding of cultural nuances is required.
- Culturally-tailored resources: Development and utilisation of culturally-tailored resources and materials for healthcare interventions, aligning with the dietary

For peer review only - http://bmjopen.bmj.com/site/about/guidelines.xhtml

preferences and cultural backgrounds of participants. For example, signposting to existing resources and literature such as the South Asian and African and Caribbean Eatwell guide.[57]

- Language-specific groups: Develop language-specific support groups and resources within healthcare programmes to cater to participants where English is not a first language.
- Diverse workforce: Within the contextual setting the programme is being delivered to promote diversity within the workforce. Having colleagues from diverse backgrounds facilitates mutual support, encouraging the exchange of knowledge and fostering interpersonal collaboration. This diversity can enable team members to ask questions, share insights, and collectively enhance their cultural competence, ultimately ensuring more effective support for the ethnically diverse participant population.
- Further research: Is needed to examine cultural and contextual influences, the long-term effects of cultural tailoring on patient outcomes, the most effective methods for cultural competency training, and the scalability of culturally sensitive interventions within broader healthcare systems. Through exploring this in future research, programmes and practices within healthcare can be improved and a more equitable service provided.
- Future research: Should detail the ratio of coaches to the total patient ethnicity or reflect the percentage of different ethnicities managed by each coach, which would provide valuable insights for a more nuanced understanding of programme effectiveness and impact across diverse populations.

For peer review only

Conclusion

In healthcare programmes, care should be person-centred, with lifestyle interventions involving culturally tailored approaches, particular in relation to dietary and lifestyle advice. There is a need to develop an understanding of different cultures and ethnicities and the factors that may contribute to inequity for different populations to support the delivery of future health programmes. By acknowledging the current limitations in training, these findings advocate for a more culturally competent approach in the education and support for health coaches, to foster more inclusive healthcare practices. Among other

For peer review only - http://bmjopen.bmj.com/site/about/guidelines.xhtml

recommendations including culturally tailored resources and language-specific groups, coaches delivering the NHS LCD Programme would benefit from enhanced training and support to equip them with the knowledge and skills necessary to tailor delivery of the programme to individuals from diverse ethnic backgrounds: reducing barriers and optimising inclusivity and effectiveness of the programme across ethnic groups.

Author statement

PD, MM, CH, KD and LE provided substantial contributions to the design of the work. PD, MM and KD provided substantial contributions to the interpretation of data for the work. All authors PD, MM, KD, CH, LE and CB drafted the work and provided revisions. All authors provided final approval of the version to be published and provided agreement to be accountable for all aspects of the work in ensuring that questions related to the accuracy or integrity of any part of the work are appropriately investigated and resolved.

Declarations

The views expressed in this paper are those of the authors and not necessarily those of the NHS or the National Institute for Health Research.

Availability of data and materials

The datasets generated during this current study are not publicly available due to reasons of privacy and confidentiality, and because of the inability to de-identify the data. Additional knowledge of the data can be available from the corresponding author on reasonable request.

Competing interests

All authors confirm that they have no conflicts of interest to declare. LE has received funding from NIHR, MRC, Leeds Council and OHID/PHE in the last 3 years, and has had an honorary contract with OHID. CB is a primary care advisor to the national diabetes programme for NHS England.

Funding

For peer review only - http://bmjopen.bmj.com/site/about/guidelines.xhtml

For peer review only

This work was supported by the National Institute for Health Research, Health Services and Delivery Research [NIHR 132075]. The NHS England Low-Calorie Diet Pilot is funded by NHS England.

Ethics approval

Ethical approval was gained from Leeds Beckett University (LBU 102077), written informed consent was obtained from participants. The Re:Mission study was approved by Health Research Authority on 5 July 2021, REC ref: 21/WM/0136.

References

1. NHS. Long Term Plan. Updated 19/09/2023. Accessed 03rd July, 2023. https://www.longtermplan.nhs.uk/
2. Organisation WH. Obesity and overweight. World Health Organisation. Accessed 10th December, 2022. https://www.who.int/news-room/fact-sheets/detail/obesity-and-overweight
3. Leung G, Stanner S. Diets of minority ethnic groups in the UK: influence on chronic disease risk and implications for prevention. *Nutrition Bulletin*. 2011;36(2):161-198. doi:10.1111/j.1467-3010.2011.01889.x
4. Organisation WH. Health Equity. Updated 11/10/2023. Accessed 10th November, 2022. https://www.who.int/health-topics/health-equity#tab=tab_1
5. Bleich SN, Jarlenski MP, Bell CN, LaVeist TA. Health inequalities: trends, progress, and policy. *Annu Rev Public Health*. Apr 2012;33:7-40. doi:10.1146/annurev-publhealth-031811-124658
6. Dharmi Kapadia JZ, Sarah Salway, James Nazroo, Andrew Booth, Nazmy Villarroel-Williams, Laia Bécares & Aneez Esmail. *Ethnic Inequalities in Healthcare: A Rapid Evidence Review*. 2022. *NHS Race and Health Observatory*. https://www.nhsrho.org/wp-content/uploads/2022/02/RHO-Rapid-Review-Final-Report_v.7.pdf
7. Schouten BC, Cox A, Duran G, et al. Mitigating language and cultural barriers in healthcare communication: Toward a holistic approach. *Patient Educ Couns*. May 8 2020;doi:10.1016/j.pec.2020.05.001
8. McGregor B, Belton A, Henry TL, Wrenn G, Holden KB. Improving Behavioral Health Equity through Cultural Competence Training of Health Care Providers. *Ethn Dis*. 2019;29(Suppl 2):359-364. doi:10.18865/ed.29.S2.359
9. Shepherd SM, Willis-Esqueda C, Newton D, Sivasubramaniam D, Paradies Y. The challenge of cultural competence in the workplace: perspectives of healthcare providers. *BMC Health Services Research*. 2019;19(1)doi:10.1186/s12913-019-3959-7
10. Jongen C, McCalman J, Bainbridge R. Health workforce cultural competency interventions: a systematic scoping review. *BMC Health Services Research*. 2018;18(1)doi:10.1186/s12913-018-3001-5
11. Kaihlanen A-M, Hietapakka L, Heponiemi T. Increasing cultural awareness: qualitative study of nurses' perceptions about cultural competence training. *BMC Nursing*. 2019;18(1)doi:10.1186/s12912-019-0363-x

For peer review only - http://bmjopen.bmj.com/site/about/guidelines.xhtml

12. Saha S, Beach MC, Cooper LA. Patient Centeredness, Cultural Competence and Healthcare Quality. *Journal of the National Medical Association*. 2008;100(11):1275-1285. doi:10.1016/s0027-9684(15)31505-4
13. Harrison R, Walton M, Chauhan A, et al. What is the role of cultural competence in ethnic minority consumer engagement? An analysis in community healthcare. *International Journal for Equity in Health*. 2019;18(1)doi:10.1186/s12939-019-1104-1
14. Ojo AS, Nnyanzi LA, Giles EL, et al. "I am not really into the government telling me what I need to eat": exploring dietary beliefs, knowledge, and practices among ethnically diverse communities in England. *BMC Public Health*. 2023;23(1):800. doi:10.1186/s12889-023-15689-6
15. Ofosu NN, Luig T, Mumtaz N, et al. Health care providers' perspectives on challenges and opportunities of intercultural health care in diabetes and obesity management: a qualitative study. *CMAJ Open*. 2023;11(4):E765-E773. doi:10.9778/cmajo.20220222
16. Thom DH, Wolf J, Gardner H, et al. A Qualitative Study of How Health Coaches Support Patients in Making Health-Related Decisions and Behavioral Changes. *The Annals of Family Medicine*. 2016;14(6):509-516. doi:10.1370/afm.1988
17. Debesay J, Kartzow AH, Fougner M. Healthcare professionals' encounters with ethnic minority patients: The critical incident approach. *Nursing Inquiry*. 2022;29(1)doi:10.1111/nin.12421
18. Dhir P, Maynard M, Drew KJ, Homer CV, Bakhai C, Ells LJ. South Asian individuals' experiences on the NHS low-calorie diet programme: a qualitative study in community settings in England. *BMJ Open*. 2023;13(12):e079939. doi:10.1136/bmjopen-2023-079939
19. Yasui M, Pottick KJ, Chen Y. Conceptualizing Culturally Infused Engagement and Its Measurement for Ethnic Minority and Immigrant Children and Families. *Clin Child Fam Psychol Rev*. Sep 2017;20(3):250-332. doi:10.1007/s10567-017-0229-2
20. Jones S, Brown TJ, Watson P, et al. Commercial provider staff experiences of the NHS low calorie diet programme pilot: a qualitative exploration of key barriers and facilitators. *BMC Health Services Research*. 2024;24(1)doi:10.1186/s12913-023-10501-y
21. Homer C KK, Drew KJ, et al. A qualitative evaluation of experiences of the Total Diet Replacement phase of the NHS Low Calorie Diet Programme pilot. *British Journal of Diabetes*. 2024;24doi:10.15277/bjd.2024.435
22. NHS. Low calorie diets to treat obesity and Type 2 diabetes. Accessed 7th March 2022, https://www.england.nhs.uk/diabetes/treatment-care/low-calorie-diets/
23. Drew KJ HC, Radley D, Freeman C, Kinsella K, Maynard M, Bakhai, C and Ells, L. . Equity and Local Health Systems – a qualitative evaluation of the experiences of Local Health Service Leads during the first two years of the NHS Low Calorie Diet Programme pilot. . *The British Journal of Diabetes*. 2023;Forthcoming.
24. Lean ME, Leslie WS, Barnes AC, et al. Primary care-led weight management for remission of type 2 diabetes (DiRECT): an open-label, cluster-randomised trial. *The Lancet*. 2018;391(10120):541-551. doi:10.1016/s0140-6736(17)33102-1
25. Astbury NM, Aveyard P, Nickless A, et al. Doctor Referral of Overweight People to Low Energy total diet replacement Treatment (DROPLET): pragmatic randomised controlled trial. *BMJ*. 2018:k3760. doi:10.1136/bmj.k3760
26. England N. Service Specification No. 1: NHS Low Calorie Diet Programme [Version 01]. 2021
27. Evans TS, Dhir P, Radley D, et al. Does the design of the NHS Low-Calorie Diet Programme have fidelity to the programme specification? A documentary review of service parameters and behaviour change content in a type 2 diabetes intervention. *Diabetic Medicine*. 2023;40(4)doi:10.1111/dme.15022
28. C Bakhai EB, T Gorton, S Safazadeh, S Jebb, P Aveyard, J Wilding, E Robertson, R Taylor, J Valabhji. The ambition for remission – the NHS Type 2 Diabetes Path to Remission Programme. DPC 20232023.

For peer review only - http://bmjopen.bmj.com/site/about/guidelines.xhtml

29. C Bakhai EB, T Gorton, S Safazadeh, S Jebb, P Aveyard, J Wilding, E Robertson, R Taylor, J Valabhji. EARLY FINDINGS FROM THE NHS TYPE 2 DIABETES PATH TO REMISSION PROGRAME (formerly NHS Low Calorie Diet Programme) 2023.

30. NIHR. A coproduced mixed method evaluation of the NHS England low calorie diet implementation pilot. Accessed 16 January, 2022. https://fundingawards.nihr.ac.uk/award/NIHR132075

31. Fletcher AJ. Applying critical realism in qualitative research: Methodology meets method. *International Journal of Social Research Methodology: Theory & Practice*. 2017;20(2):181-194. doi:10.1080/13645579.2016.1144401

32. Statistics OfN. (2011a). 2011 rural/urban classification. Accessed 03/10/2023, https://www.ons.gov.uk/methodology/geography/geographicalproducts/ruralurbanclassifications/2011ruralurbanclassification

33. Clarke VBaV. *Thematic Analysis: A practical guide*. SAGE; 2022.

34. Malterud K, Siersma VD, Guassora AD. Sample Size in Qualitative Interview Studies: Guided by Information Power. *Qual Health Res*. Nov 2016;26(13):1753-1760. doi:10.1177/1049732315617444

35. Braun V, Clarke V. To saturate or not to saturate? Questioning data saturation as a useful concept for thematic analysis and sample-size rationales. *Qualitative Research in Sport, Exercise and Health*. 2021/03/04 2021;13(2):201-216. doi:10.1080/2159676X.2019.1704846

36. Netto G, Bhopal R, Lederle N, Khatoon J, Jackson A. How can health promotion interventions be adapted for minority ethnic communities? Five principles for guiding the development of behavioural interventions. *Health Promotion International*. 2010;25(2):248-257. doi:10.1093/heapro/daq012

37. Resnicow K, Baranowski, T., Ahluwalia, J.S. and Braithwaite, R.L. Cultural Sensitivity in Public Health: Defined and Demystified. *Ethnicity and Disease*. 1999;9:10-21.

38. Joo JY, Liu MF. Culturally tailored interventions for ethnic minorities: A scoping review. *Nursing Open*. 2021;8(5):2078-2090. doi:10.1002/nop2.733

39. Litchfield I, Barrett T, Hamilton-Shield J, et al. Current evidence for designing self-management support for underserved populations: an integrative review using the example of diabetes. *International Journal for Equity in Health*. 2023/09/11 2023;22(1):188. doi:10.1186/s12939-023-01976-6

40. Bentley P, Jovanovic A, Sharma P. Cultural diversity training for UK healthcare professionals: a comprehensive nationwide cross-sectional survey. *Clin Med (Lond)*. Oct 2008;8(5):493-7. doi:10.7861/clinmedicine.8-5-493

41. Brach C, Fraser I. Can cultural competency reduce racial and ethnic health disparities? A review and conceptual model. *Med Care Res Rev*. 2000;57 Suppl 1(Suppl 1):181-217. doi:10.1177/1077558700057001s09

42. Memon A, Taylor K, Mohebati LM, et al. Perceived barriers to accessing mental health services among black and minority ethnic (BME) communities: a qualitative study in Southeast England. *BMJ Open*. 2016;6(11):e012337. doi:10.1136/bmjopen-2016-012337

43. Goff LM. Ethnicity and Type 2 diabetes in the UK. *Diabetic Medicine*. 2019;36(8):927-938. doi:https://doi.org/10.1111/dme.13895

44. Creamer J, Attridge M, Ramsden M, Cannings-John R, Hawthorne K. Culturally appropriate health education for Type 2 diabetes in ethnic minority groups: an updated Cochrane Review of randomized controlled trials. *Diabet Med*. Feb 2016;33(2):169-83. doi:10.1111/dme.12865

45. Wallia S, Bhopal RS, Douglas A, et al. Culturally adapting the prevention of diabetes and obesity in South Asians (PODOSA) trial. *Health Promot Int*. Dec 2014;29(4):768-79. doi:10.1093/heapro/dat015

46. Goff LM, Moore AP, Harding S, Rivas C. Development of Healthy Eating and Active Lifestyles for Diabetes, a culturally tailored diabetes self-management education and support programme for

For peer review only - http://bmjopen.bmj.com/site/about/guidelines.xhtml

Black-British adults: A participatory research approach. *Diabet Med*. Nov 2021;38(11):e14594. doi:10.1111/dme.14594

47. Betancourt JR, Green AR, Carrillo JE, Ananeh-Firempong O, 2nd. Defining cultural competence: a practical framework for addressing racial/ethnic disparities in health and health care. *Public Health Rep*. Jul-Aug 2003;118(4):293-302. doi:10.1093/phr/118.4.293

48. Truong M, Yeganeh L, Cook O, Crawford K, Wong P, Allen J. Using telehealth consultations for healthcare provision to patients from non-Indigenous racial/ethnic minorities: a systematic review. *Journal of the American Medical Informatics Association*. 2022;29(5):970-982. doi:10.1093/jamia/ocac015

49. Dragomanovich HM, Shubrook JH. Improving Cultural Humility and Competency in Diabetes Care for Primary Care Providers. *Clin Diabetes*. Apr 2021;39(2):220-224. doi:10.2337/cd20-0063

50. Pettersson S, Holstein J, Jirwe M, Jaarsma T, Klompstra L. Cultural competence in healthcare professionals, specialised in diabetes, working in primary healthcare—A descriptive study. *Health & Social Care in the Community*. 2022;30(3)doi:10.1111/hsc.13442

51. Hamed S, Bradby H, Ahlberg BM, Thapar-Björkert S. Racism in healthcare: a scoping review. *BMC Public Health*. May 16 2022;22(1):988. doi:10.1186/s12889-022-13122-y

52. Danso A, Danso Y. The complexities of race and health. *Future Healthc J*. Mar 2021;8(1):22-27. doi:10.7861/fhj.2020-0225

53. Napier D, Depledge M, Knipper M, et al. *Culture matters: using a cultural contexts of health approach to enhance policy-making*. 2017.

54. Tee S, Üzar-Özçetin YS, Trenoweth S. Achieving culturally competent mental health care: A mixed-methods study drawing on the perspectives of UK nursing students. *Perspect Psychiatr Care*. Oct 2022;58(4):1267-1280. doi:10.1111/ppc.12926

55. Handtke O, Schilgen B, Mösko M. Culturally competent healthcare - A scoping review of strategies implemented in healthcare organizations and a model of culturally competent healthcare provision. *PLoS One*. 2019;14(7):e0219971. doi:10.1371/journal.pone.0219971

56. FitzGerald C, Hurst S. Implicit bias in healthcare professionals: a systematic review. *BMC Med Ethics*. Mar 1 2017;18(1):19. doi:10.1186/s12910-017-0179-8

57. Jay F. The South Asian Eatwell Guide. Accessed 11 November, 2023. https://mynutriweb.com/the-south-asian-eatwell-guide/

For peer review only

For peer review only - http://bmjopen.bmj.com/site/about/guidelines.xhtml

Consolidated criteria for reporting qualitative studies (COREQ): 32-item checklist

No. Item	Guide questions/description	Reported on Page #
**Domain 1: Research team and reflexivity**		
Personal Characteristics		
1. Interviewer/facilitator	Which author/s conducted the interview or focus group?	Page 7
2. Credentials	What were the researcher's credentials? E.g. PhD, MD	Page 1
3. Occupation	What was their occupation at the time of the study?	Page 1
4. Gender	Was the researcher male or female?	Page 7
5. Experience and training	What experience or training did the researcher have?	Page 7
Relationship with participants		
6. Relationship established	Was a relationship established prior to study commencement?	Page 6-8
7. Participant knowledge of the interviewer	What did the participants know about the researcher? e.g. personal goals, reasons for doing the research	Page 7
8. Interviewer characteristics	What characteristics were reported about the interviewer/facilitator? e.g. Bias, assumptions, reasons and interests in the research topic	Page 7

For peer review only

**Domain 2: study design**		
Theoretical framework		
9. Methodological orientation and Theory	What methodological orientation was stated to underpin the study? e.g. grounded theory, discourse analysis, ethnography, phenomenology, content analysis	Page 6
Participant selection		
10. Sampling	How were participants selected? e.g. purposive, convenience, consecutive, snowball	Page 7
11. Method of approach	How were participants approached? E.g. face-to-face, telephone, mail, email	Page 7
12. Sample size	How many participants were in the study?	Page 7
13. Non-participation	How many people refused to participate or dropped out? Reasons?	None
Setting		
14. Setting of data collection	Where was the data collected? e.g. home, clinic, workplace	Page 7
15. Presence of non-participants	Was anyone else present besides the participants and researchers?	Page 7
16. Description of sample	What are the important characteristics of the sample? E.g. demographic data, date	Page 6-7.
Data collection		
17. Interview guide	Were questions, prompts, guides provided by the authors? Was it pilot tested?	Page 7 and supplementary file 2.
18. Repeat interviews	Were repeat interviews carried out? If yes, how many?	No
19. Audio/visual recording	Did the research use audio or visual recording to collect the data?	Page 8
20. Field notes	Were field notes made during and/or after the interview or focus group?	Page 8
21. Duration	What was the duration of the interviews or focus group?	Page 8
22. Data saturation	Was data saturation discussed?	N/A
23. Transcripts returned	Were transcripts returned to participants for comment and/or correction?	No
**Domain 3: analysis and**		

For peer review only

**findings**		
Data analysis		
24. Number of data coders	How many data coders coded the data?	Page 8
25. Description of the coding tree	Did authors provide a description of the coding tree?	Page 8. Supplementary file 3 and 4.
26. Derivation of themes	Were themes identified in advance or derived from the data?	Derived from the data.
27. Software	What software, if applicable, was used to manage the data?	Page 8
28. Participant checking	Did participants provide feedback on the findings?	No
Reporting		
29. Quotations presented	Were participant quotations presented to illustrate the themes/findings? Was each quotation identified? e.g. participant number	Page 9-12
30. Data and findings consistent	Was there consistency between the data presented and the findings?	Page 9-15
31. Clarity of major themes	Were major themes clearly presented in the findings?	Page 9-12
32. Clarity of minor themes	Is there a description of diverse cases or discussion of minor themes?	N/A

Developed from:
Tong A, Sainsbury P, Craig J. Consolidated criteria for reporting qualitative research (COREQ): a 32-item checklist for interviews and focus groups. *International Journal for Quality in Health Care*. 2007. Volume 19, Number 6: pp. 349 – 357

Personal Characteristics:

Pooja Dhir (Female, British Indian). PhD student of 2 years and registered dietitian for 7 years.

Professor Maria Maynard (Female, Black Caribbean). Professor of Health Inequalities with extensive experience of ethnicity and health research.

Dr Kevin J Drew PhD (Male, White British). Post-doctoral Research Fellow with 7 years' experience of conducting qualitative evaluations of health-based interventions.

Dr Catherine Homer PhD (Female, White British). Senior Research Fellow with experience working in academia and extensive experience working in public health.

Dr Chirag Bakhai (Male, Indian). General Practitioner in primary care with a special interest in diabetes management. Chirag is a clinical lead on the Oversight group for the Re:Mission Study

For peer review only

and a primary care advisor to the national diabetes programme for NHS England and NHS Improvement.

Dr Louisa Ells (Female, White British). Professor of Obesity with a specialist interest in multi-disciplinary, cross-sector applied obesity research, with extensive experience of leading programme evaluations.

For peer review only

Topic 1: Closed Background questions

What programme provider are they part of and what is your role with the provider ?

About their ethnicity?

Topic 2

1. Can you tell me how well you think the programme works for minoritised populations?
   - i. Can you describe your role on the programme?
   - ii. Can you give any examples of what you think works well?
   - iii. Is there anything that doesn't work well?
   - iv. What are your thoughts on the uptake and adherence of individuals from these groups on the programme?
   - v. Do people from ethnic minority populations attend the sessions you have delivered?
   - vi. If not what do they perceive are the barriers to being in the programme?
2. What training have you received to help you deliver to ethnic populations?
   - i. How did you find the support that was provided?
   - vii. Can you tell me about any training you have had to work with people from ethnic populations?
3. How is the programme tailored to support people from ethnic groups to access, engage and adhere?
   - i. What impacts have you seen for individuals from the specific tailoring?
   - ii. Does the overall programme work for ethnic populations why / why not?
4. Can you describe what have been your experiences of delivering the programme to individuals from ethnic backgrounds?
   - i. Can you describe what has worked well?
   - ii. Can you describe any challenges?
   - iii. Can you describe what changes were made to support this population?
   - iv. How has the programme or the delivery been tailored for ethnic populations?
   - v. Were you able to resolve the barriers, and if so how?
   - vi. How have the challenges you faced impacted your delivery of the programme?
5. How do you think the programme and delivery could be improved for ethnic populations?
   - i. Can you describe your experiences of being a coach and delivering to ethnic populations - what works well / challenges / additional support needs?
6. What considerations, if any do you think have been made to support ethnic populations in completing the programme?

i. Can you describe what further considerations you think need to be made?

ii. Can you describe what you think of the considerations that have already been made?

iii. Can you tell me what improvements you think are needed to the programme to support those from ethnic populations?

Any questions?

For peer review only - http://bmjopen.bmj.com/site/about/guidelines.xhtml

Population living with type 2 diabetes.

In this section demographic information on the population living with diabetes is provided, following an estimated population that meet the eligibility criteria of the Low Calorie Diet programme. Figures in Table 1 are taken from the pilot site prospectuses compiled in mid-2019. Birmingham and Solihull, and North London Partners in Health and Care referenced the national Diabetes Audit (NDA) 17-18 as the source of their figures. Gloucestershire reference a Local Diabetes Audit 17-18, while Derbyshire referenced RAIDR 2019. The other six pilot sites do not provide a reference for where this information has been taken from.

Table 1. Population living with type 2 diabetes

Pilot Sites	People with Type 2 Diabetes	% Of Total Population
South Yorkshire and Bassetlaw	93,090	6.2%
Frimley Health and Care	29,950	3.7%
Greater Manchester HSCP	156,625	5.6%
Joined Up Care Derbyshire	50,273	5.0%
Birmingham and Solihull	74,250	5.7%
East London Health and Care Partnership	116,096	5.3%
Humber Coast and Vale	82,426	5.9%
Gloucestershire	27,309	4.2%
Bedfordshire, Luton and Milton Keynes	48,445	4.8%
North London Partners in Health and Care	69,530	4.3%
**Total:**	**747,994**	

For peer review only

Table 2: Overview of the LCD programme

Time	Three Phases of the Low Calorie Diet Programme
1-12 Weeks	**LCD:** with total diet replacement products (TDR) up to 900 calories per day. During this time patients will replace all normal meals with these products.
12-18 Weeks	**Food re-introduction:** during this time patients will be supported to re-introduce food, with a stepped phasing out of TDR products.
18-52 Weeks	**Maintenance**, during this time patients will be supported to maintain their initial weight loss.

During the TDR phase patients are expected to replace all normal meals with TDR products, which are provided by the provider for the duration of the service. Each service provider provides different TDR products such as soups, shakes, bars, and meal replacements such as porridge and daal, which all come in different flavours.

Ethnicity

The distribution of type 2 diabetes amongst different ethnic groups varies. The data in Table 3 are taken from the NDA, and so are based on the registers of patients collected and held at GP practices rather than directly from census data. Percentages may not total 100 because ethnicity has not been recorded in all cases.

Table 3: Ethnicity at pilot site level

Pilot sites	CCGs (2019/2020)	% with Type 2 diabetes by:	
		White	***Minority Ethnic***
South Yorkshire and Bassetlaw	NHS Barnsley	97.0	1.7
	NHS Bassetlaw	80.9	2.8
	NHS Doncaster	88.8	5.9
	NHS Rotherham	83.9	8.0
	NHS Sheffield	73.3	18.8
Frimley Health and Care	NHS East Berkshire	50.6	43.4
	NHS NE Hampshire and Farnham	67.6	17.6
	NHS Surrey Heath	67.5	16.2

For peer review only

Pilot sites	CCGs (2019/2020)	% with Type 2 diabetes by:	
		White	*Minority Ethnic*
Greater Manchester	NHS Bolton	62.9	29.4
	NHS Bury	56.5	12.7
	NHS Heywood, Middleton, Rochdale	66.3	30.6
	NHS Manchester	49.8	44.1
	NHS Oldham	60.7	35.1
	NHS Salford	75.4	10.0
	NHS Stockport	66.0	10.9
	NHS Tameside and Glossop	78.2	13.8
	NHS Trafford	64.8	20.7
	NHS Wigan Borough	86.2	4.1
Joined Up Care Derbyshire	NHS Derby and Derbyshire	79.5	9.9
Birmingham and Solihull	NHS Birmingham and Solihull	50.8	41.2
East London Health and Care Partnership	NHS Barking and Dagenham	41.8	54.9
	NHS City and Hackney	25.2	74.4
	NHS Havering	70.0	22.0
	NHS Newham	16.2	83.1
	NHS Redbridge	25.1	72.9
	NHS Tower Hamlets	20.1	79.0
	NHS Waltham Forest	31.3	63.5
Humber Coast and Vale	NHS East Riding of Yorkshire	74.7	3.9
	NHS Hull	83.5	5.5
	NHS North East Lincolnshire	84.6	11.1
	NHS North Lincolnshire	81.1	11.7
	NHS North Yorkshire	74.0	2.4
	NHS Vale of York	75.1	2.7
Gloucestershire	NHS Gloucestershire	75.5	5.6
Bedfordshire, Luton, Milton Keynes	NHS Bedfordshire	64.4	17.7
	NHS Luton	35.4	58.2
	NHS Milton Keynes	55.5	27.2
North London Partners in Health and Care	NHS North Central London	41.4	53.2

References

1. NHS Digital. (2020a) [MI] Ethnic Category Coverage. Available from <https://digital.nhs.uk/data-and-information/publications/statistical/mi-ethnic-category-coverage/current > [Accessed on 17th May 2021].
2. Office for National Statistics. (2011b). 2011 rural/urban classification. Available from <https://www.ons.gov.uk/peoplepopulationandcommunity/culturalidentity/ethnicity> [Accessed via SHAPE on 22nd June 2021].

For peer review only

Theme: Experiences of cultural tailoring.

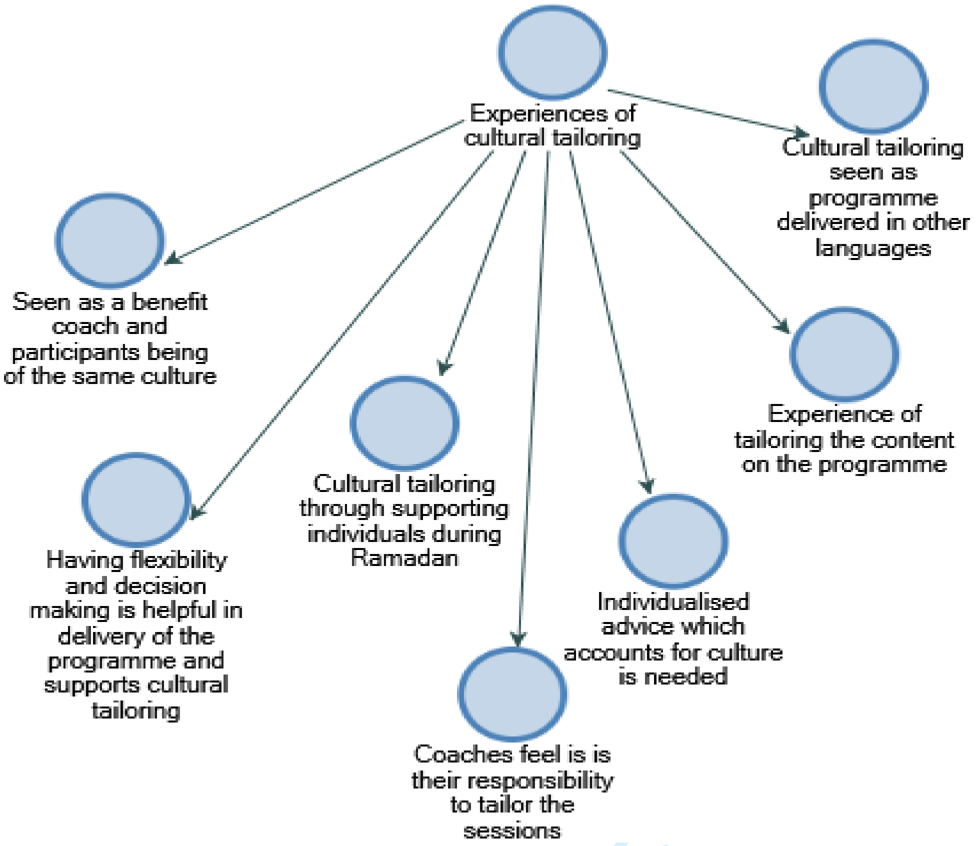

Theme: Needing to understand the impact of culture and ethnicity.

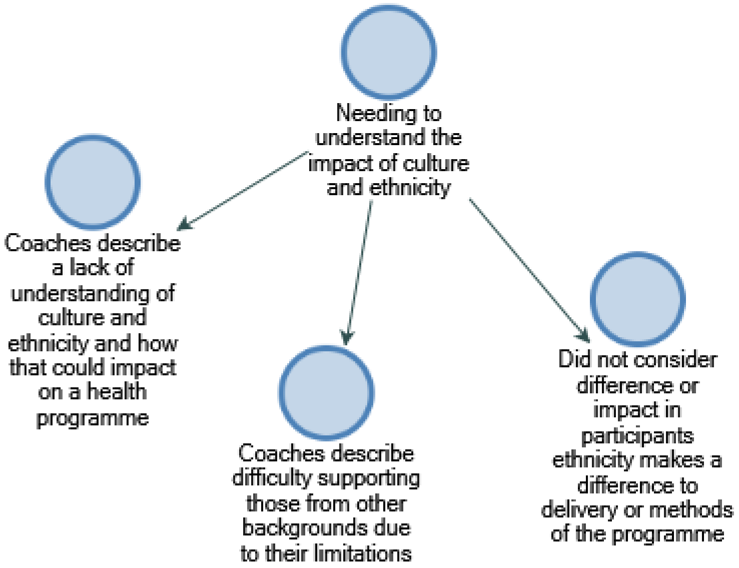

Theme: The impact of language.

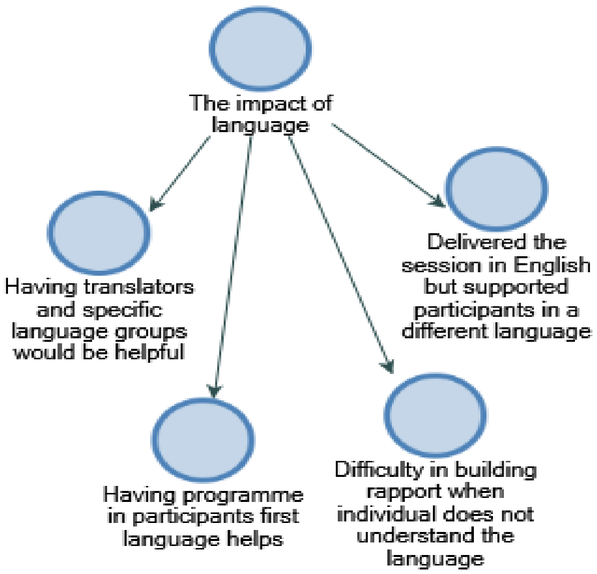

Theme: The use of resources in providing dietary advice.

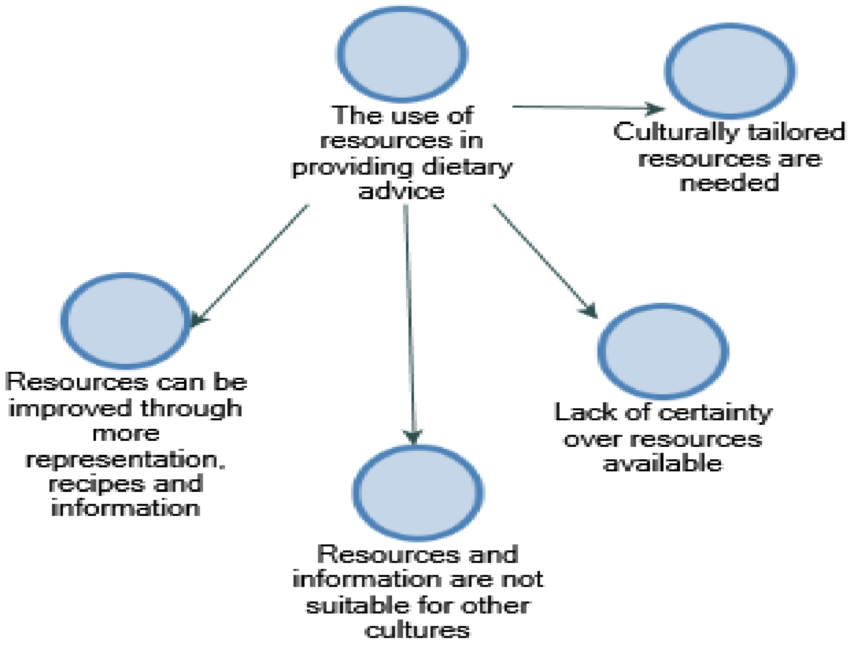

Theme: Training and support needs.

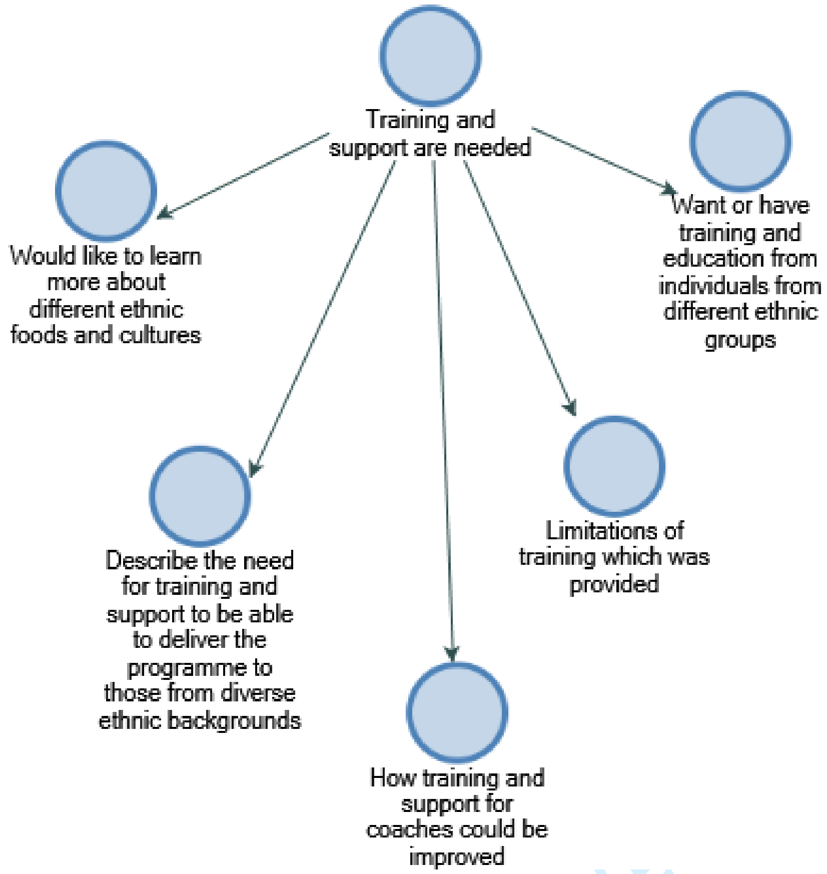

Supplement: Author's manuscript [file bmjopen-2024-085200.draft_revisions.pdf]
